# Supplementary figures and images for: CaMKII autophosphorylation can occur between holoenzymes without subunit exchange (part 1 of 2)
Source: eLife. 2023 Aug 11;12:e86090. doi: 10.7554/eLife.86090 (PMC10468207; doi:10.7554/eLife.86090)

Figure 1E

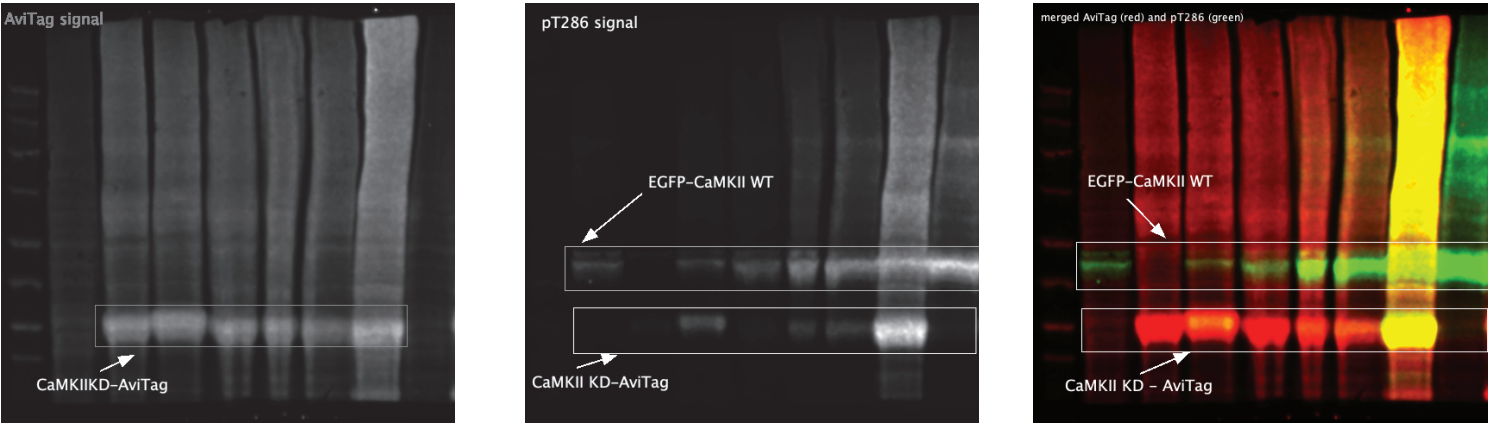

Supplement: Figure 1—source data 1. [file elife-86090-fig1-data1.zip › Figure 1-source data 1/Figure 1-source data 1.pdf]

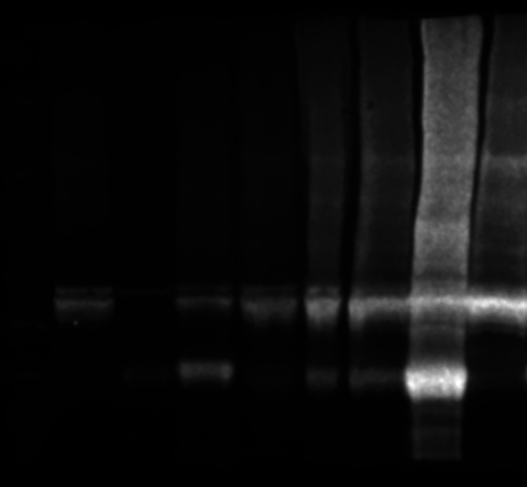

Supplement: Figure 1—source data 1. [file elife-86090-fig1-data1.zip › Figure 1-source data 1/Figure 1E pT286 blot.tif]

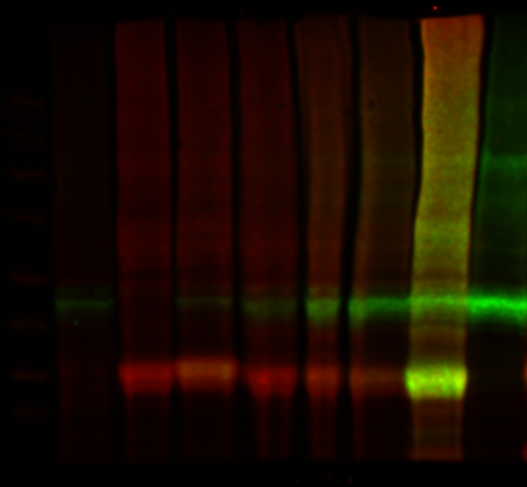

Supplement: Figure 1—source data 1. [file elife-86090-fig1-data1.zip › Figure 1-source data 1/Figure 1E merged signals.tif]

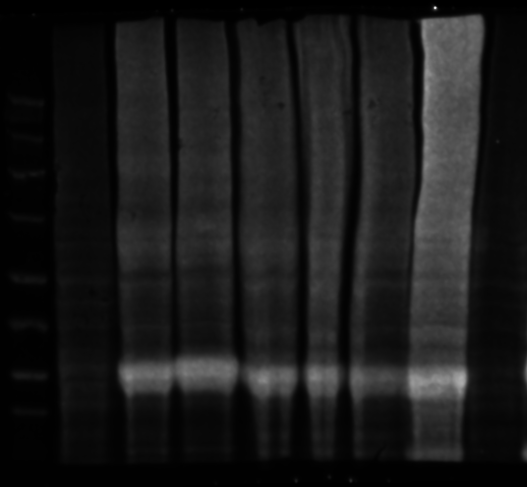

Supplement: Figure 1—source data 1. [file elife-86090-fig1-data1.zip › Figure 1-source data 1/Figure 1E AviTag blot.tif]

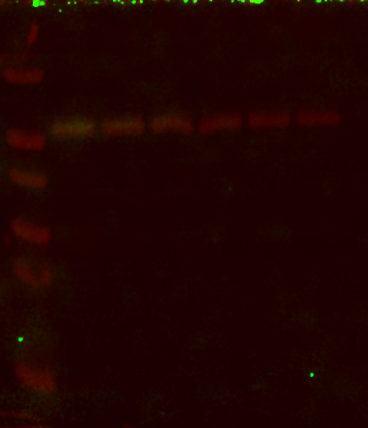

Supplement: Figure 1—figure supplement 1—source data 1. [file elife-86090-fig1-figsupp1-data1.zip › Figure 1-figure supplement 1-source data 1/Figure 1 - Figure supplement 1 C merged signals.tif]

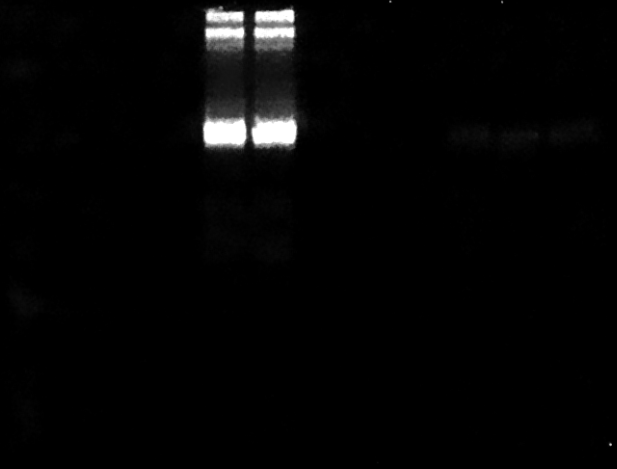

Supplement: Figure 1—figure supplement 1—source data 1. [file elife-86090-fig1-figsupp1-data1.zip › Figure 1-figure supplement 1-source data 1/Figure 1 - Figure supplement 1 D pT286 signal.tif]

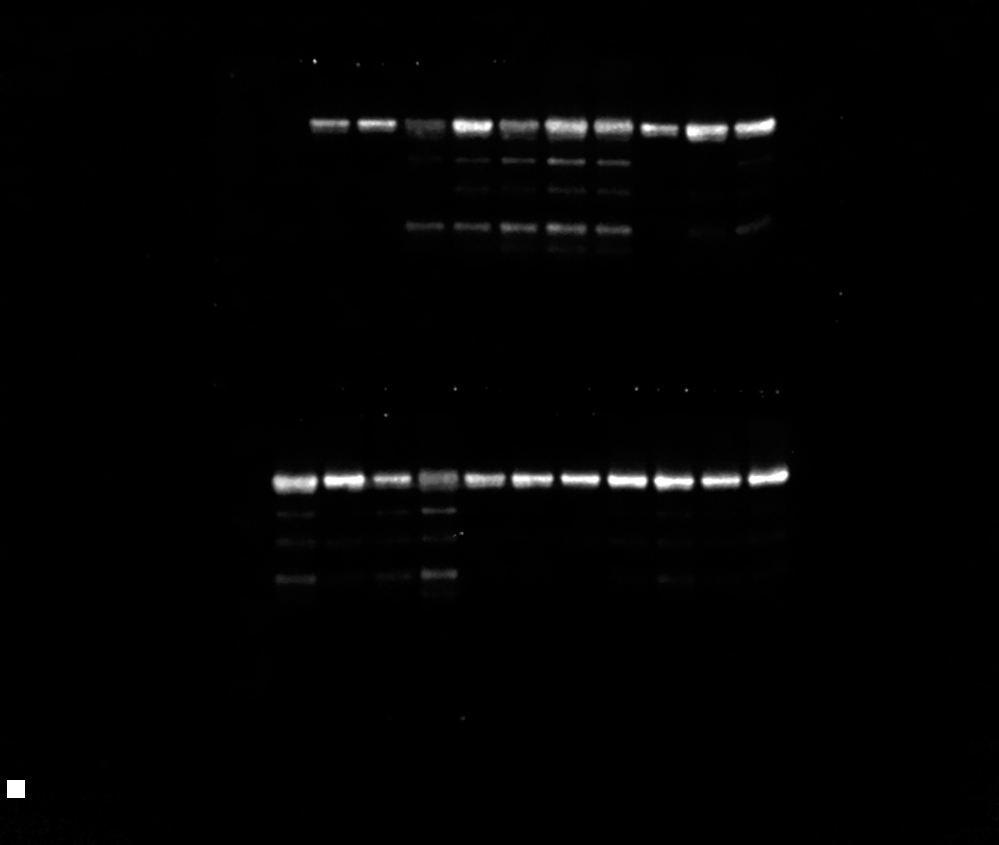

Supplement: Figure 1—figure supplement 1—source data 1. [file elife-86090-fig1-figsupp1-data1.zip › Figure 1-figure supplement 1-source data 1/Figure 1 - Figure supplement 1 A pT286 signal.tif]

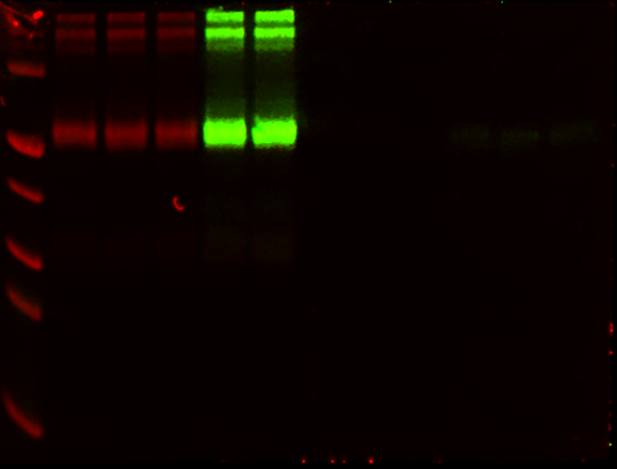

Supplement: Figure 1—figure supplement 1—source data 1. [file elife-86090-fig1-figsupp1-data1.zip › Figure 1-figure supplement 1-source data 1/Figure 1- Figure supplement 1 D merged signal.tif]

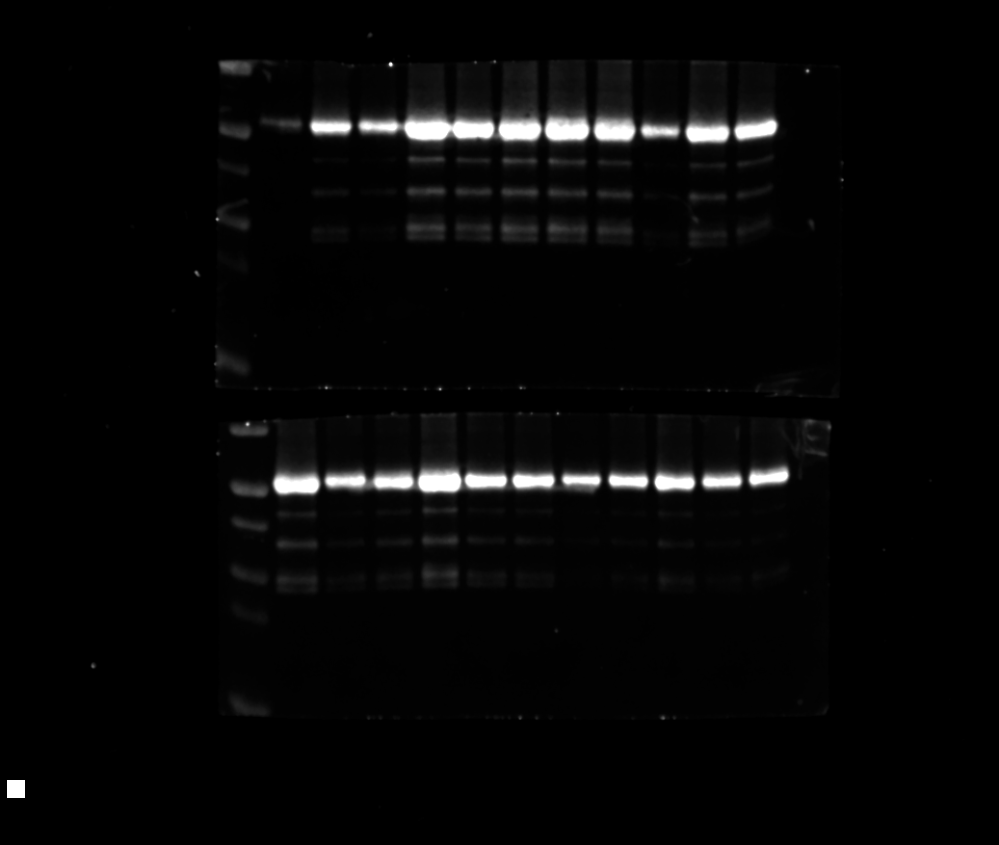

Supplement: Figure 1—figure supplement 1—source data 1. [file elife-86090-fig1-figsupp1-data1.zip › Figure 1-figure supplement 1-source data 1/Figure 1 - Figure supplement 1 A pan CaMKII.tif]

Figure 1 - figure supplement 1 blots

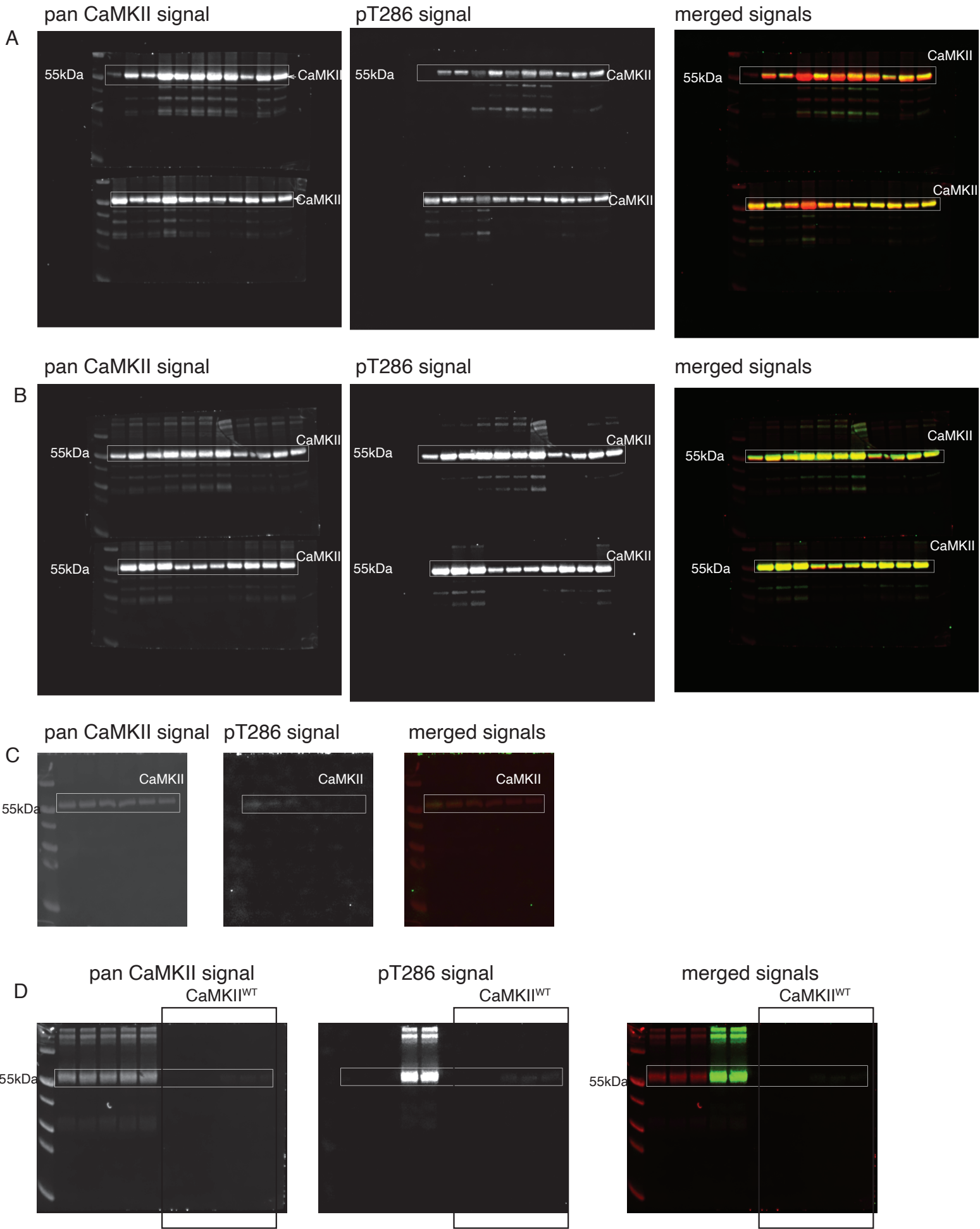

Supplement: Figure 1—figure supplement 1—source data 1. [file elife-86090-fig1-figsupp1-data1.zip › Figure 1-figure supplement 1-source data 1/Figure 1 - figure supplement 1-source data 1.pdf]

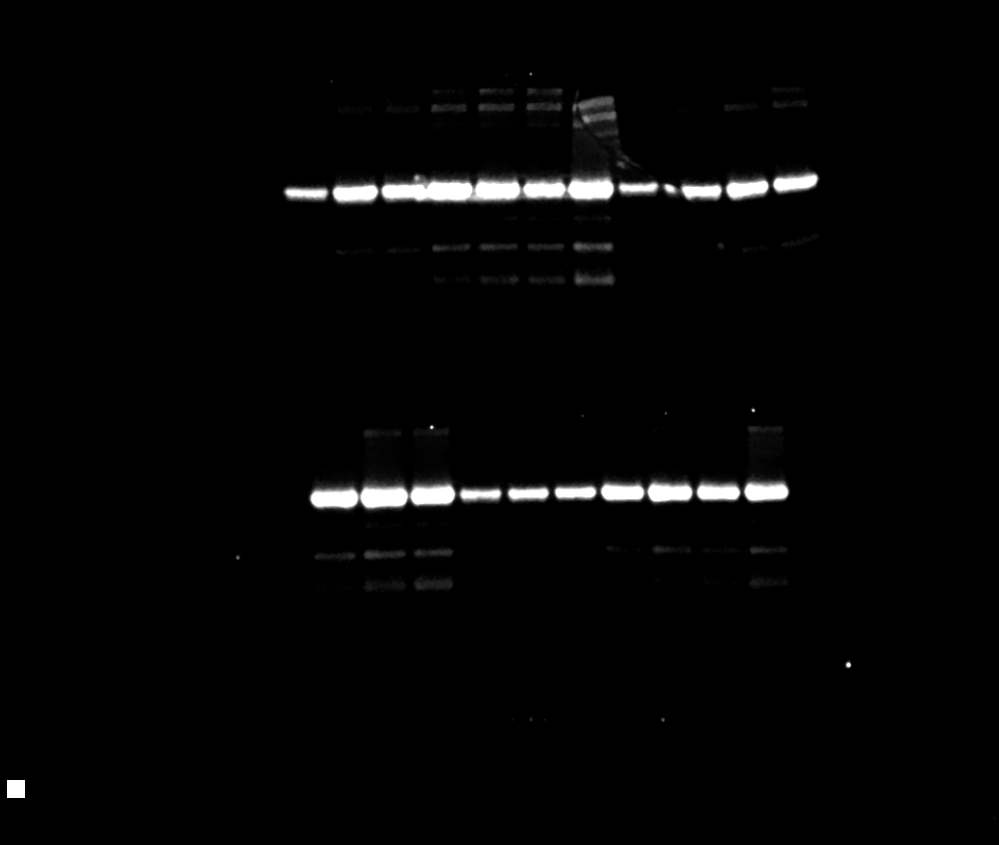

Supplement: Figure 1—figure supplement 1—source data 1. [file elife-86090-fig1-figsupp1-data1.zip › Figure 1-figure supplement 1-source data 1/Figure 1 - Figure supplement 1 B pT286 signal.tif]

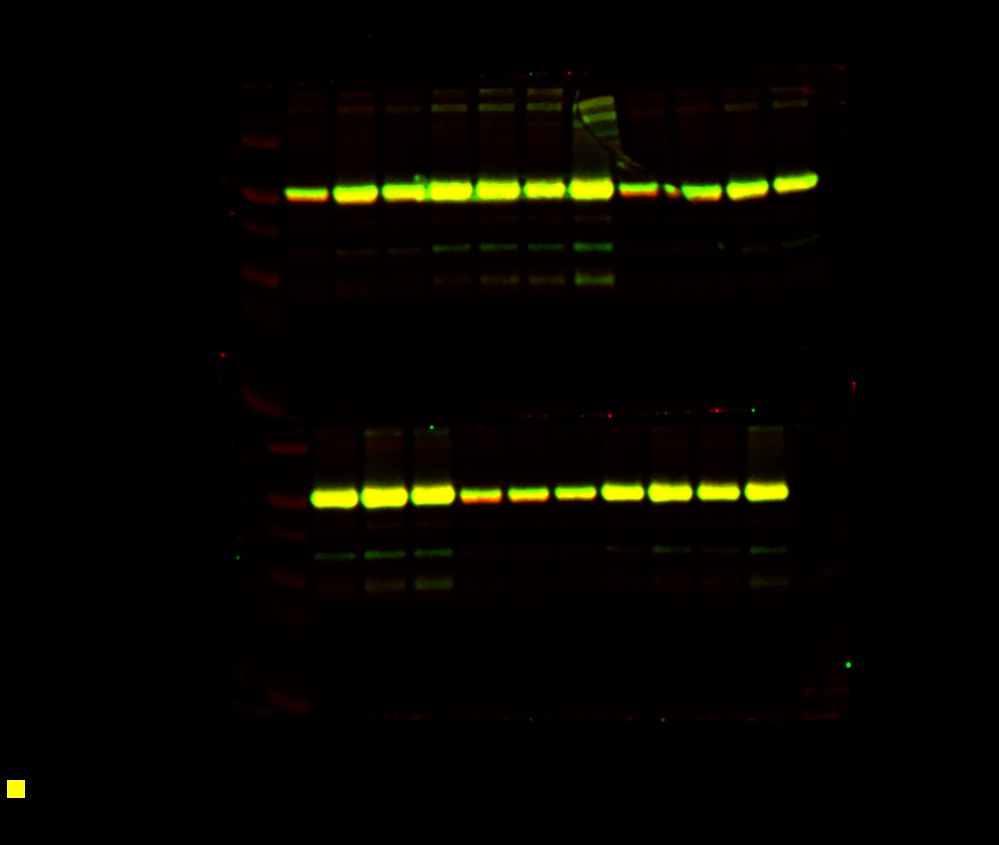

Supplement: Figure 1—figure supplement 1—source data 1. [file elife-86090-fig1-figsupp1-data1.zip › Figure 1-figure supplement 1-source data 1/Figure 1 - Figure supplement 1 B merged signals.tif]

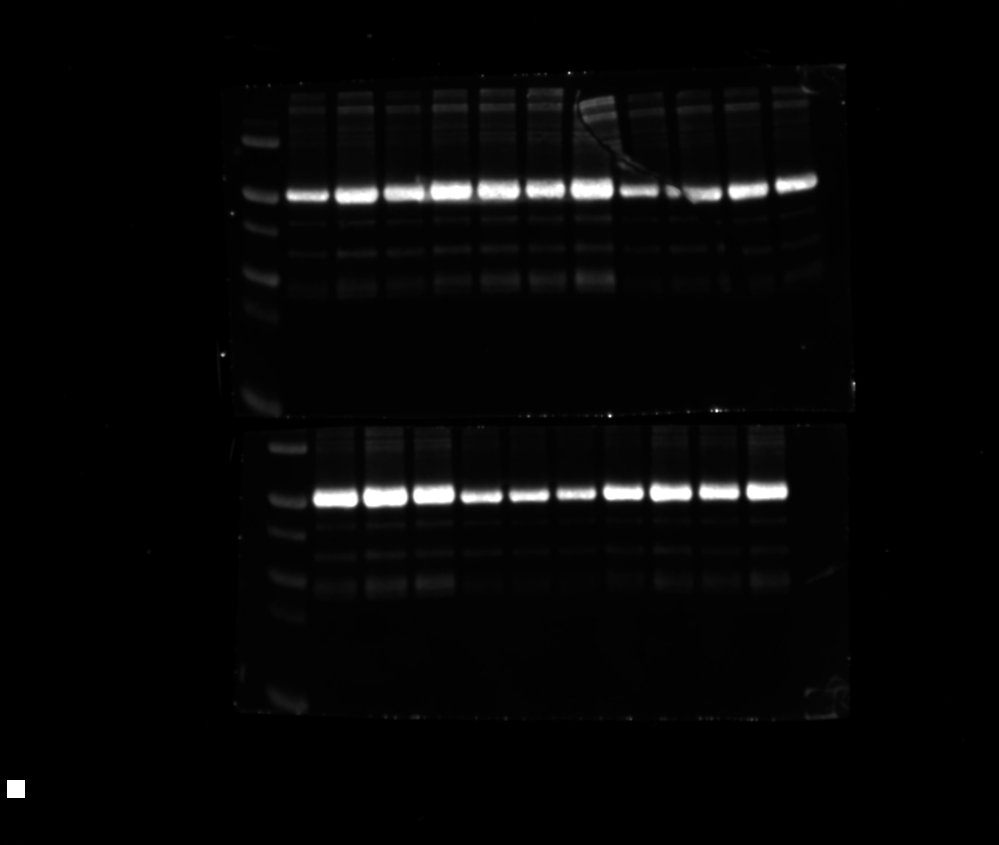

Supplement: Figure 1—figure supplement 1—source data 1. [file elife-86090-fig1-figsupp1-data1.zip › Figure 1-figure supplement 1-source data 1/Figure 1 - Figure supplement 1 B pan CaMKII.tif]

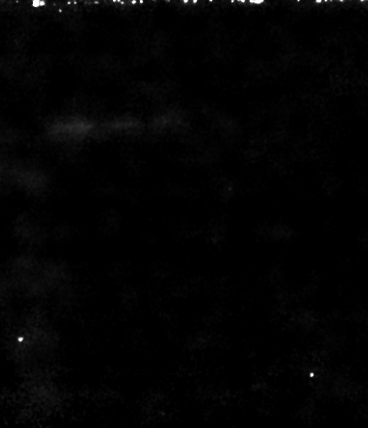

Supplement: Figure 1—figure supplement 1—source data 1. [file elife-86090-fig1-figsupp1-data1.zip › Figure 1-figure supplement 1-source data 1/Figure 1 - Figure supplement 1 C pT286 signal.tif]

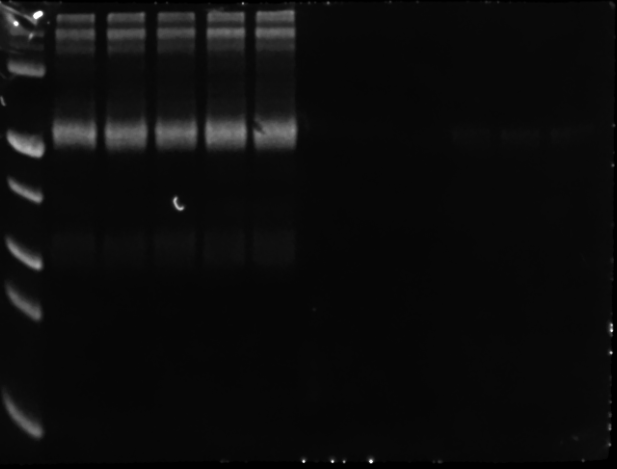

Supplement: Figure 1—figure supplement 1—source data 1. [file elife-86090-fig1-figsupp1-data1.zip › Figure 1-figure supplement 1-source data 1/Figure 1 - Figure supplement 1 D pan CaMKII.tif]

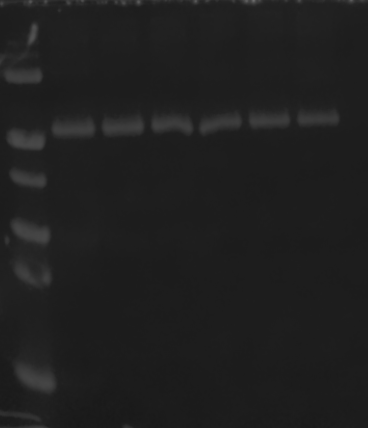

Supplement: Figure 1—figure supplement 1—source data 1. [file elife-86090-fig1-figsupp1-data1.zip › Figure 1-figure supplement 1-source data 1/Figure 1 - Figure supplement 1 C pan CaMKII.tif]

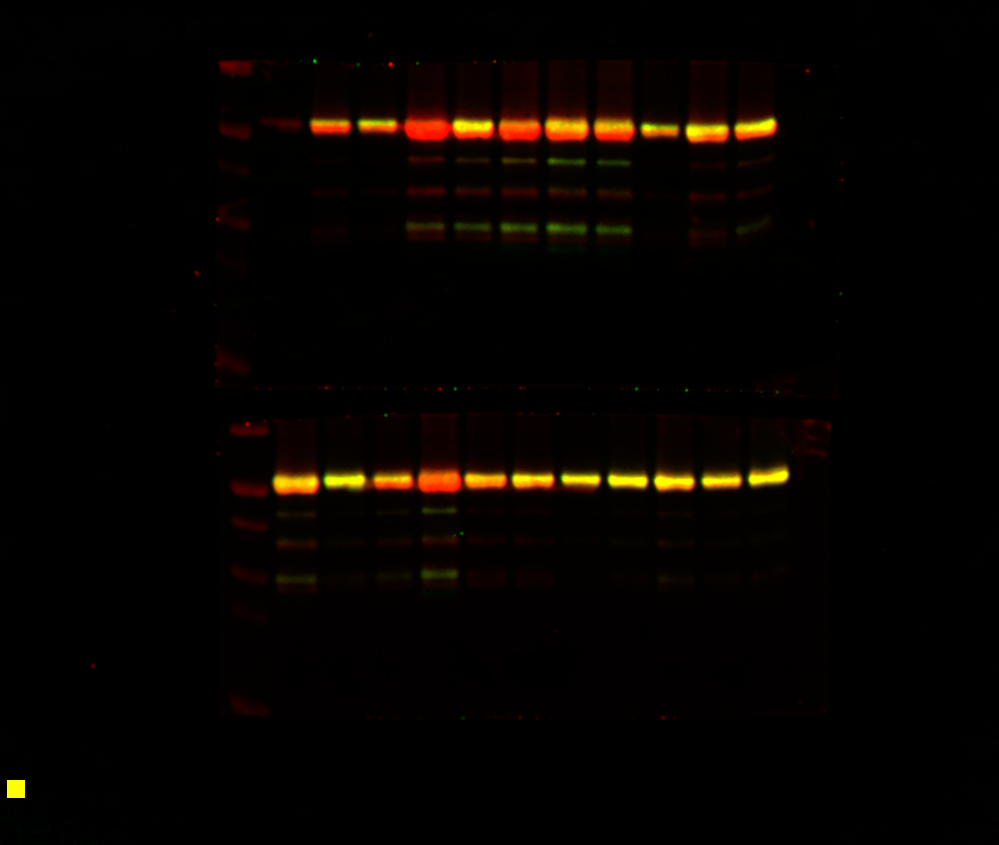

Supplement: Figure 1—figure supplement 1—source data 1. [file elife-86090-fig1-figsupp1-data1.zip › Figure 1-figure supplement 1-source data 1/Figure 1 - Figure supplement 1 A merged signals.tif]

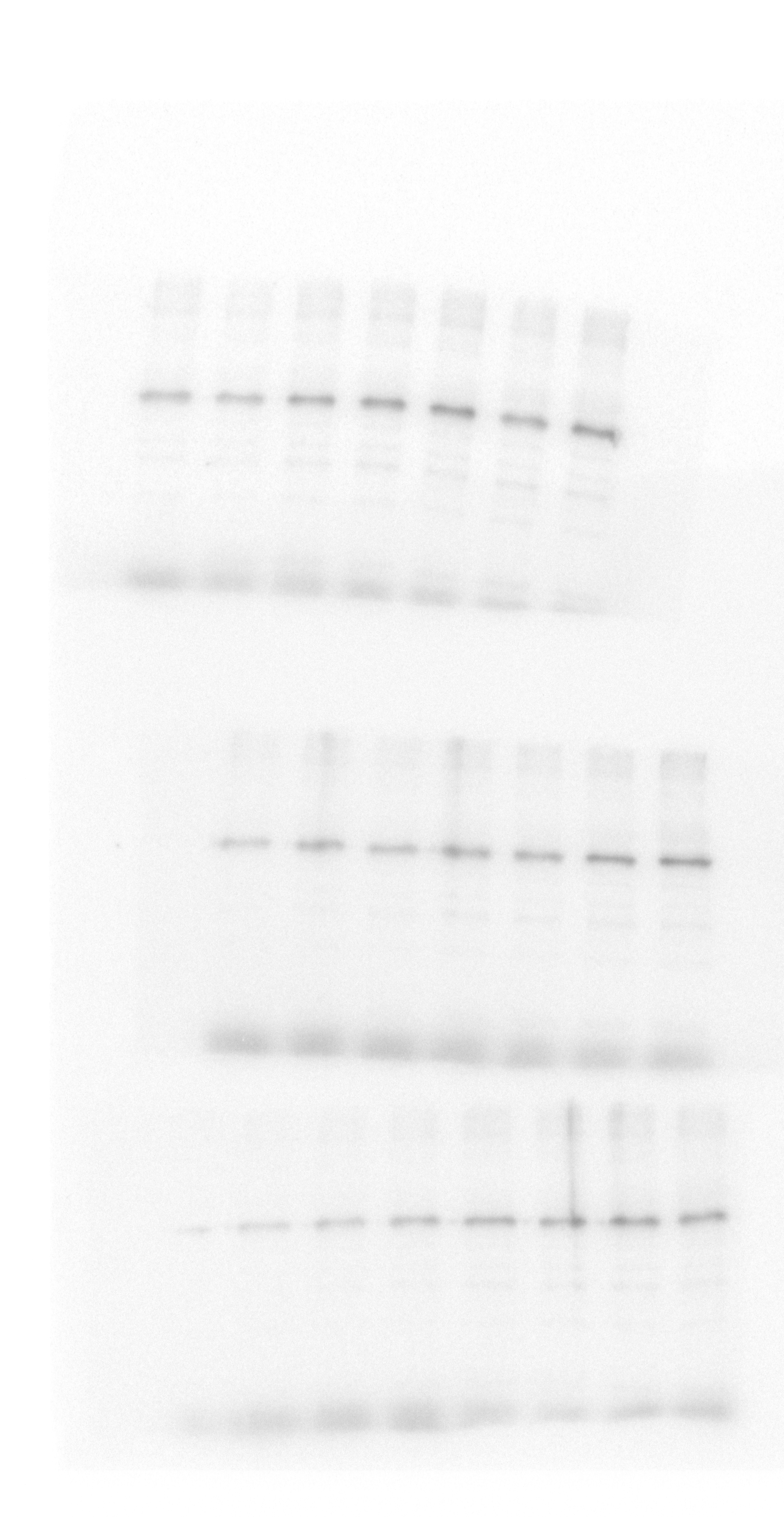

Supplement: Figure 1—figure supplement 2—source data 1. [file elife-86090-fig1-figsupp2-data1.zip › Figure 1-figure supplement 2-source data 1/Figure 1 - figure supplement 2 A gels.tif]

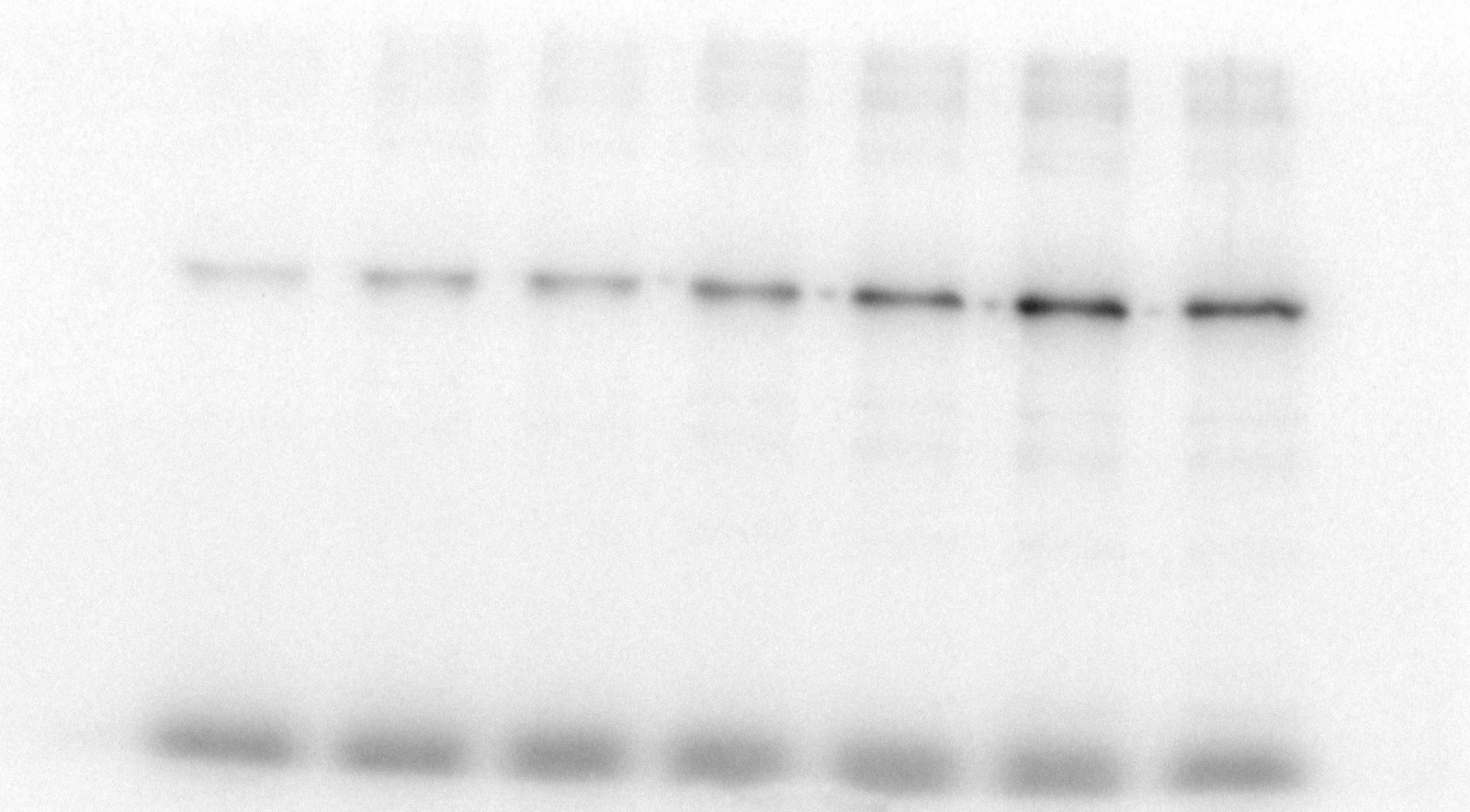

Supplement: Figure 1—figure supplement 2—source data 1. [file elife-86090-fig1-figsupp2-data1.zip › Figure 1-figure supplement 2-source data 1/Figure 1 - figure supplement 2 B_Replicate 1_gel.tif]

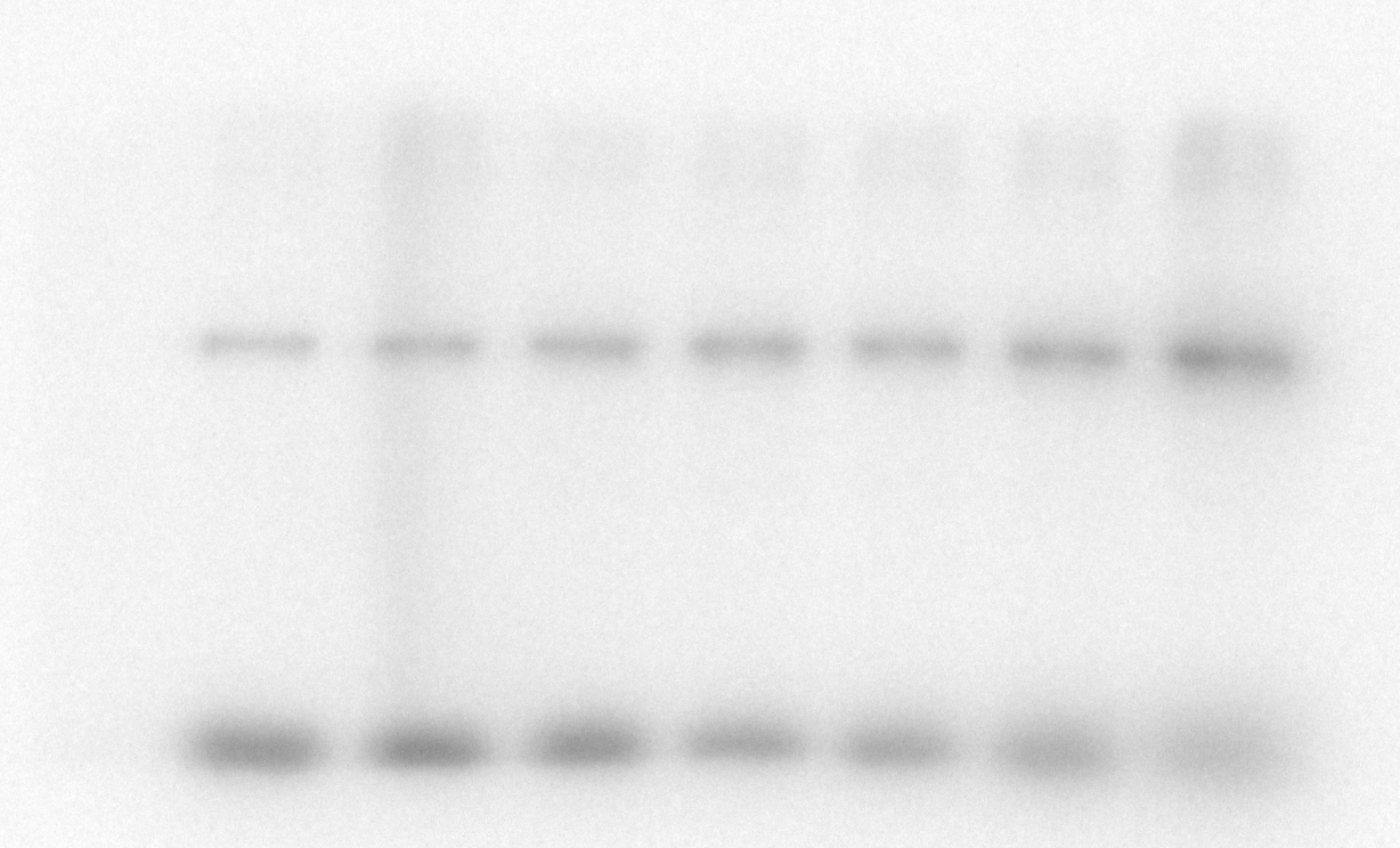

Supplement: Figure 1—figure supplement 2—source data 1. [file elife-86090-fig1-figsupp2-data1.zip › Figure 1-figure supplement 2-source data 1/Figure 1 - figure supplement 2 B_Replicate 3_gel.tif]

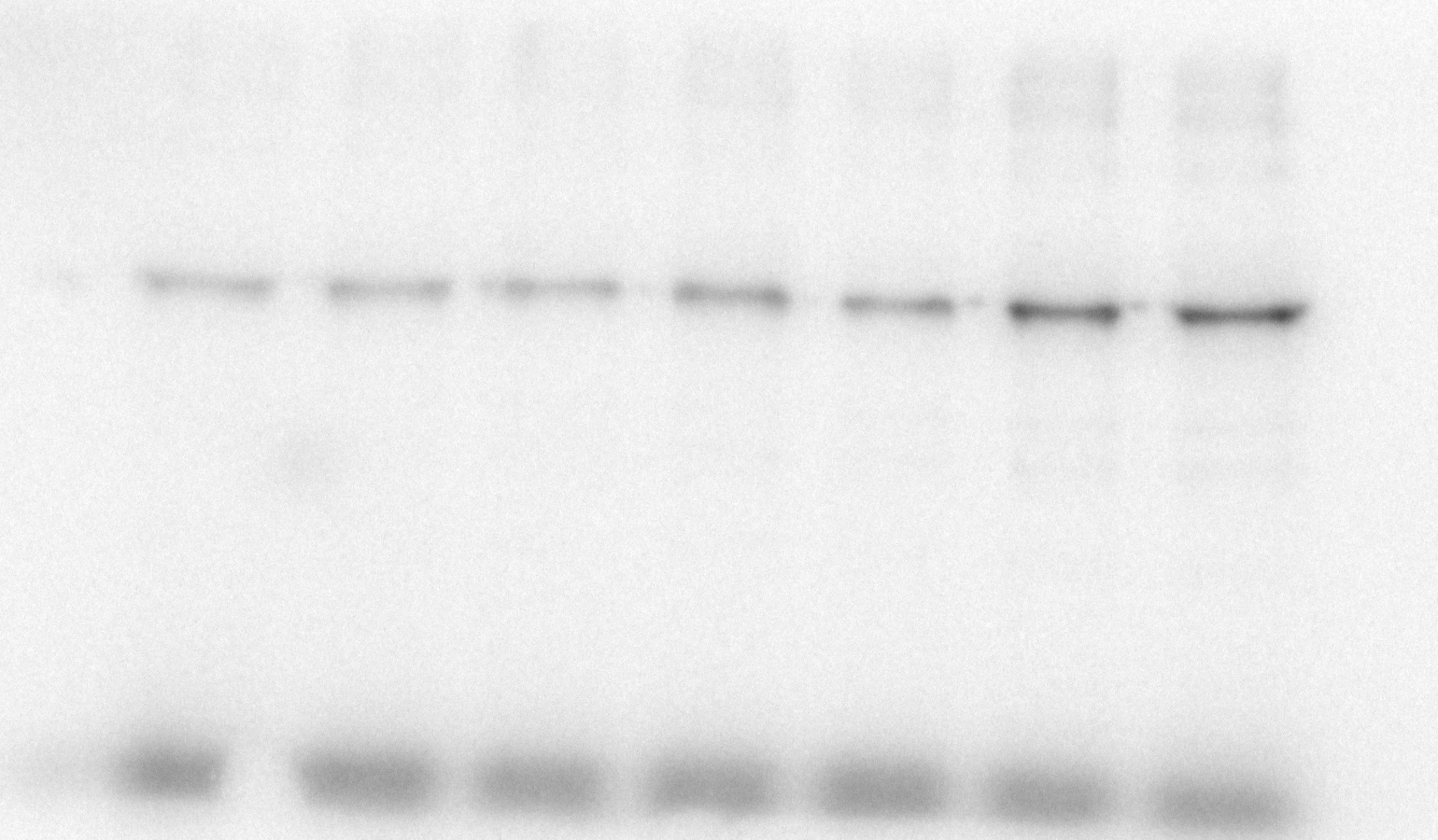

Supplement: Figure 1—figure supplement 2—source data 1. [file elife-86090-fig1-figsupp2-data1.zip › Figure 1-figure supplement 2-source data 1/Figure 1 - figure supplement 2 B_Replicate 2_gel.tif]

Figure 1 - figure supplemet 2 gels

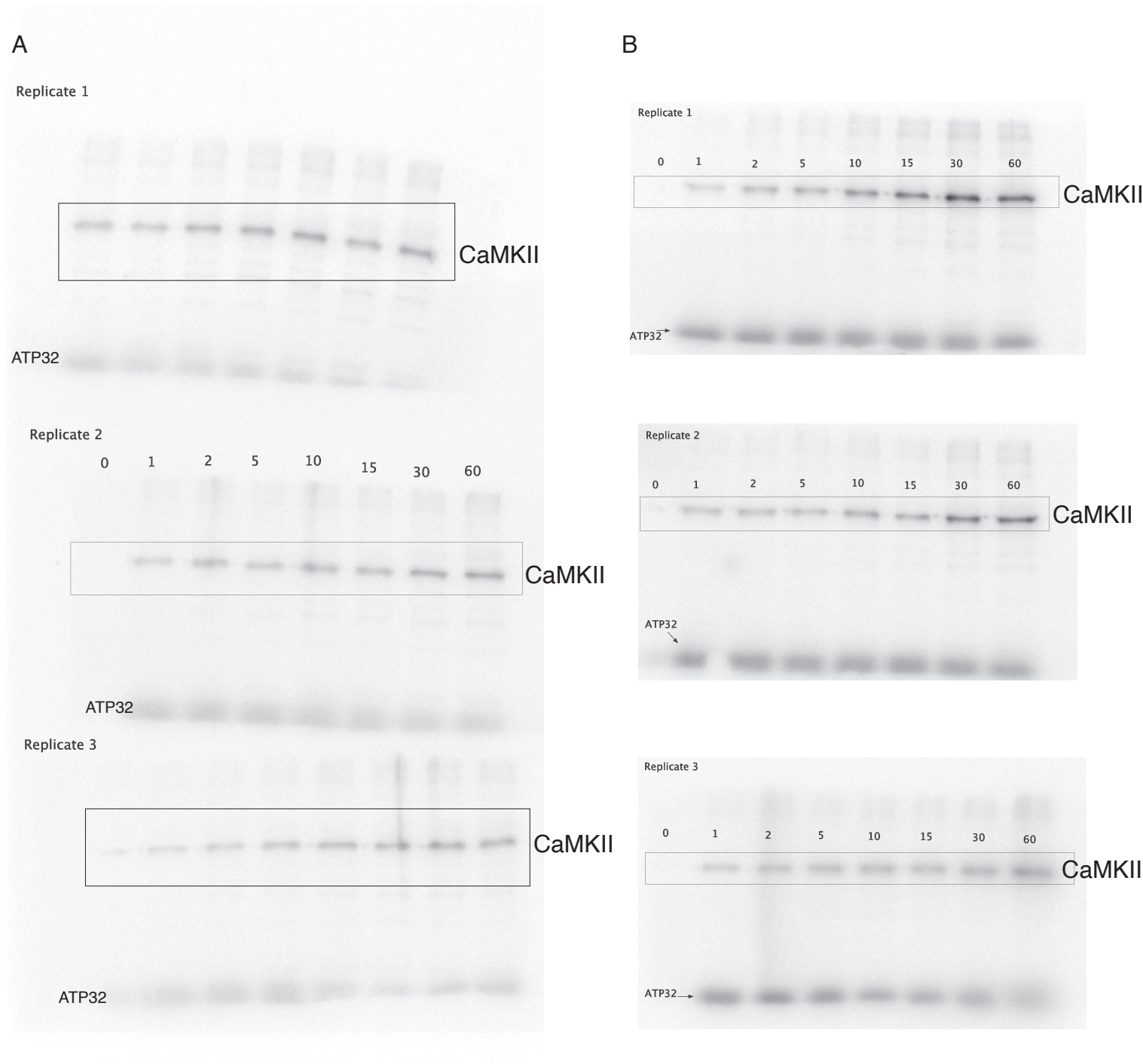

Supplement: Figure 1—figure supplement 2—source data 1. [file elife-86090-fig1-figsupp2-data1.zip › Figure 1-figure supplement 2-source data 1/Figure 1 - figure supplement 2-source data 1.pdf]

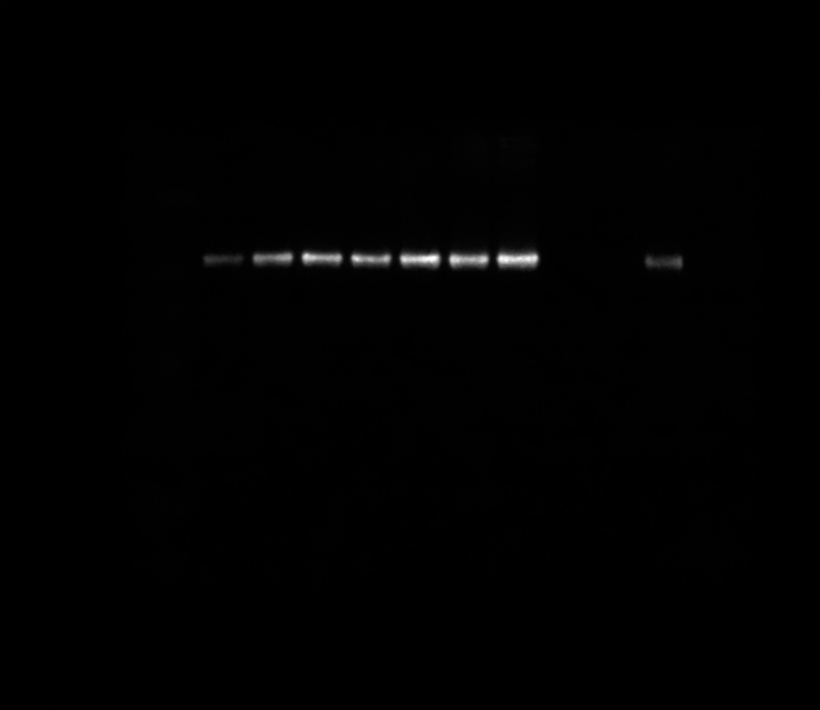

Supplement: Figure 1—figure supplement 3—source data 1. [file elife-86090-fig1-figsupp3-data1.zip › Figure 1-figure supplement 3-source data 1/Figure 1 - figure supplement 3_2 nM pT286 blot.tif]

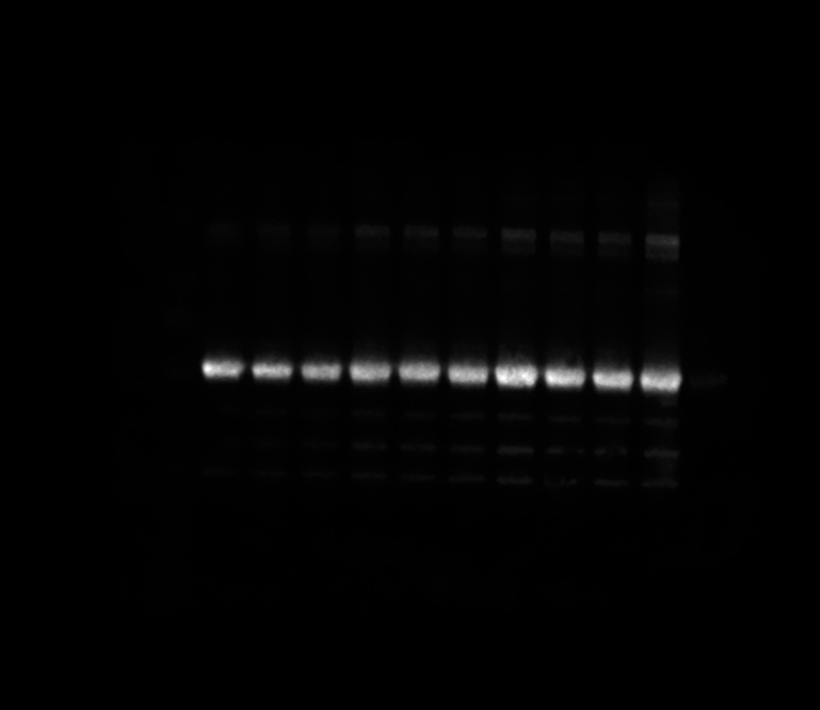

Supplement: Figure 1—figure supplement 3—source data 1. [file elife-86090-fig1-figsupp3-data1.zip › Figure 1-figure supplement 3-source data 1/Figure 1 - figure supplement 3_100 nM pT286_blot.tif]

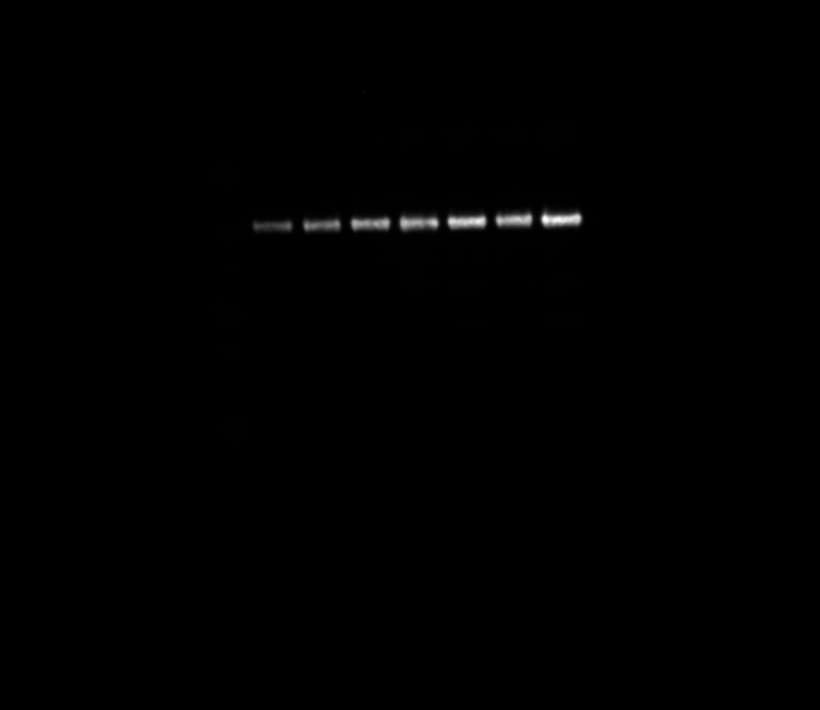

Supplement: Figure 1—figure supplement 3—source data 1. [file elife-86090-fig1-figsupp3-data1.zip › Figure 1-figure supplement 3-source data 1/Figure 1 - figure supplement 3_10 nM pT286_blot.tif]

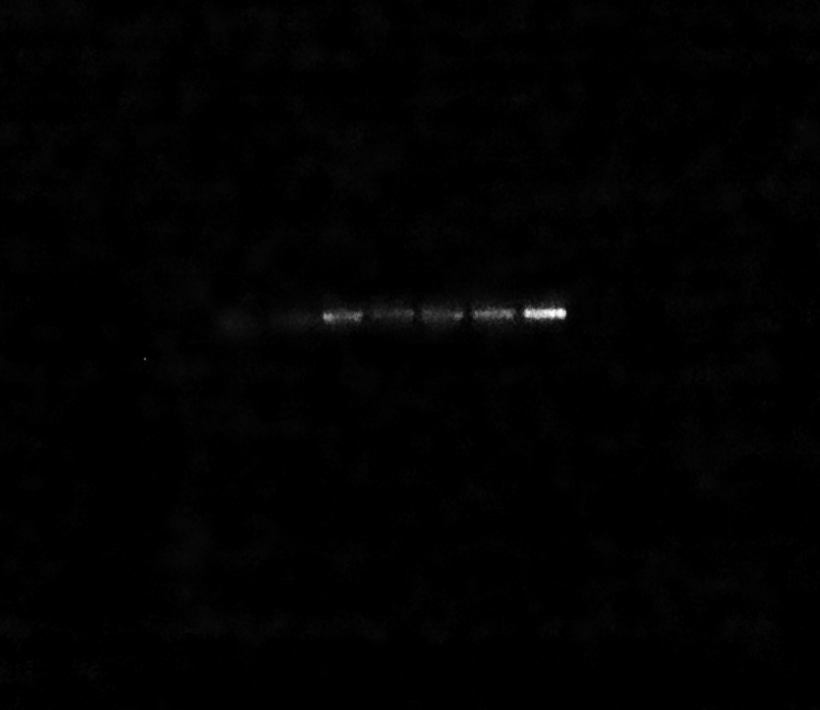

Supplement: Figure 1—figure supplement 3—source data 1. [file elife-86090-fig1-figsupp3-data1.zip › Figure 1-figure supplement 3-source data 1/Figure 1 - figure supplement 3_0.5nM pT286 blot.tif]

Figure 1 - figure supplement 3 blots

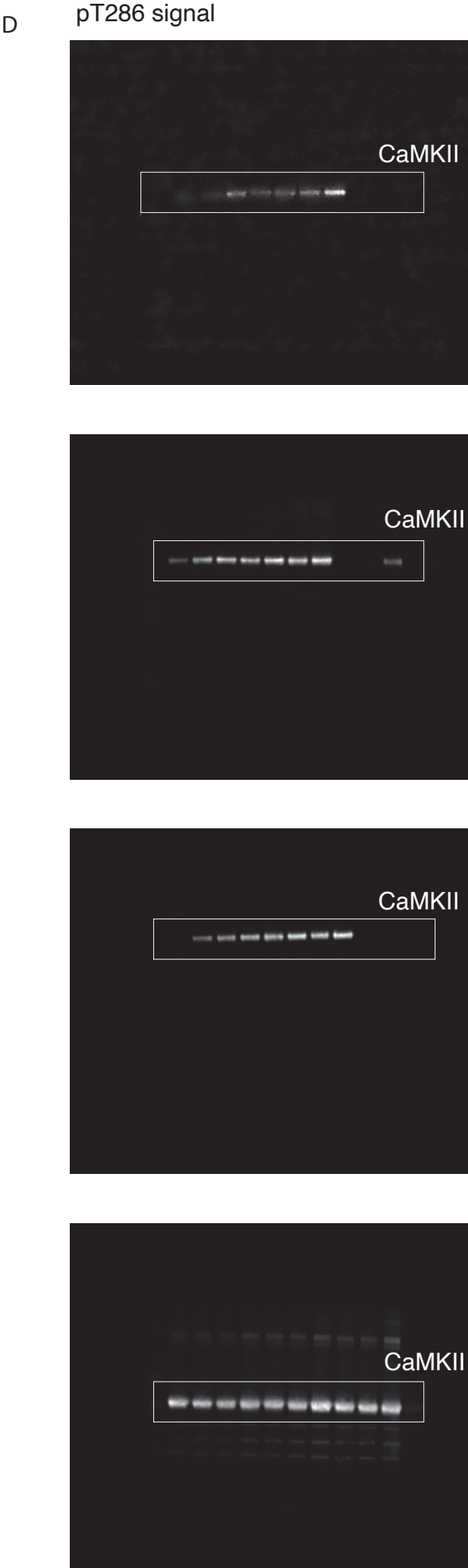

Supplement: Figure 1—figure supplement 3—source data 1. [file elife-86090-fig1-figsupp3-data1.zip › Figure 1-figure supplement 3-source data 1/Figure 1 - figure supplement 3-source data 1.pdf]

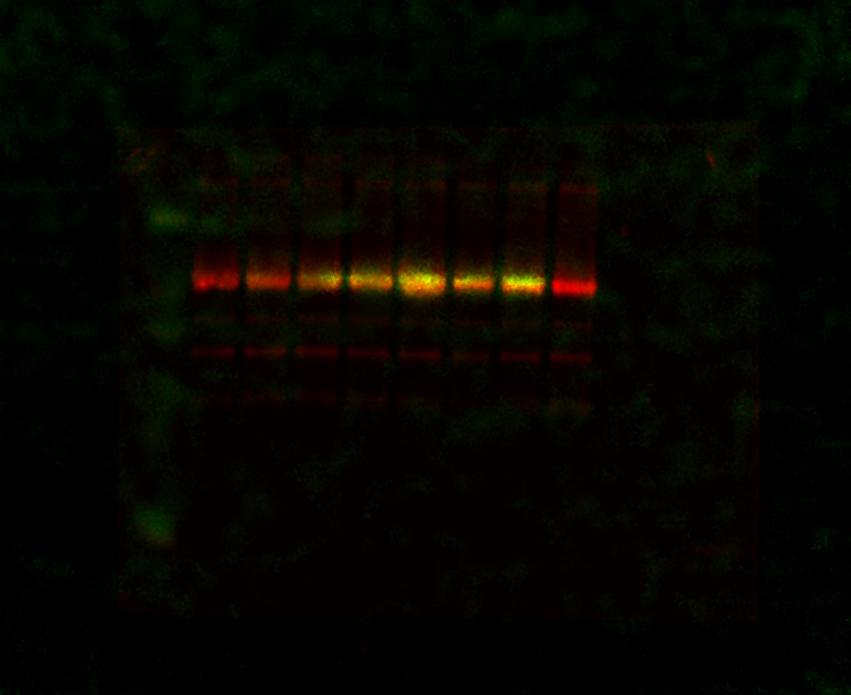

Supplement: Figure 1—figure supplement 4—source data 1. [file elife-86090-fig1-figsupp4-data1.zip › Figure 1-figure supplement 4-source data 1/Figure 1 - figure supplement 4_rep 1_merged.tif]

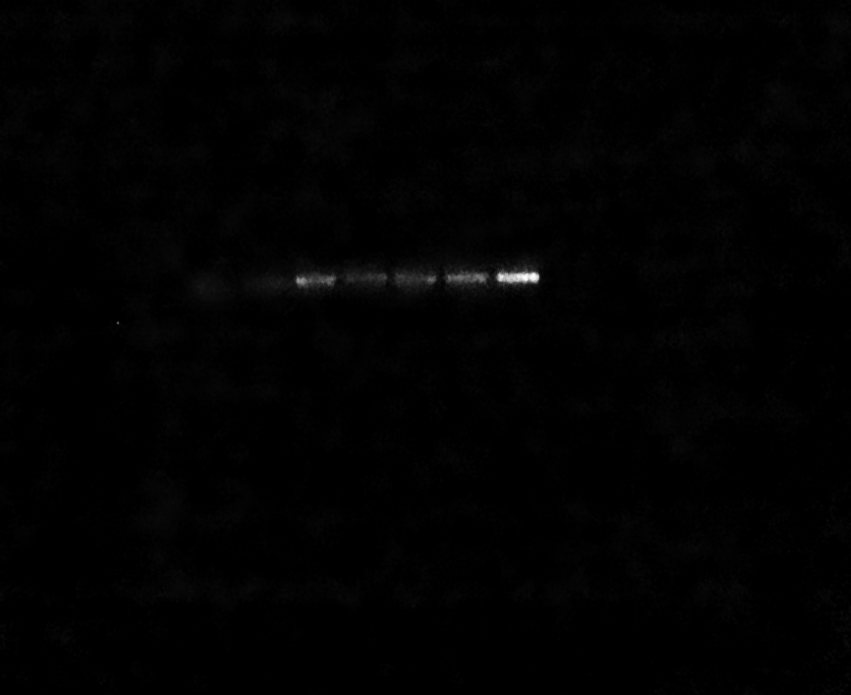

Supplement: Figure 1—figure supplement 4—source data 1. [file elife-86090-fig1-figsupp4-data1.zip › Figure 1-figure supplement 4-source data 1/Figure 1 - figure supplement 4_rep 2_pT286.tif]

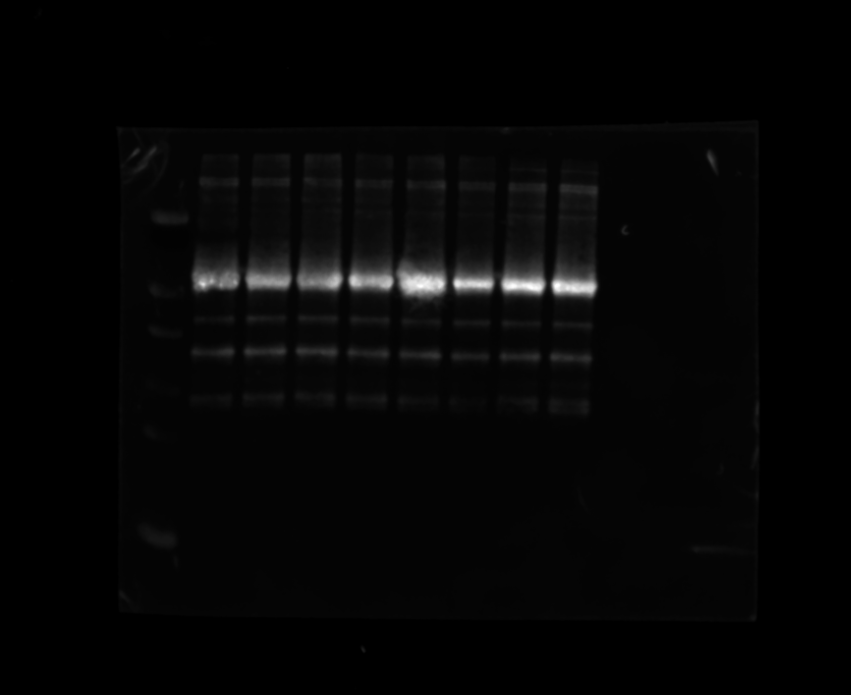

Supplement: Figure 1—figure supplement 4—source data 1. [file elife-86090-fig1-figsupp4-data1.zip › Figure 1-figure supplement 4-source data 1/Figure 1 - figure supplement 4_rep 1_pan CaMKII.tif]

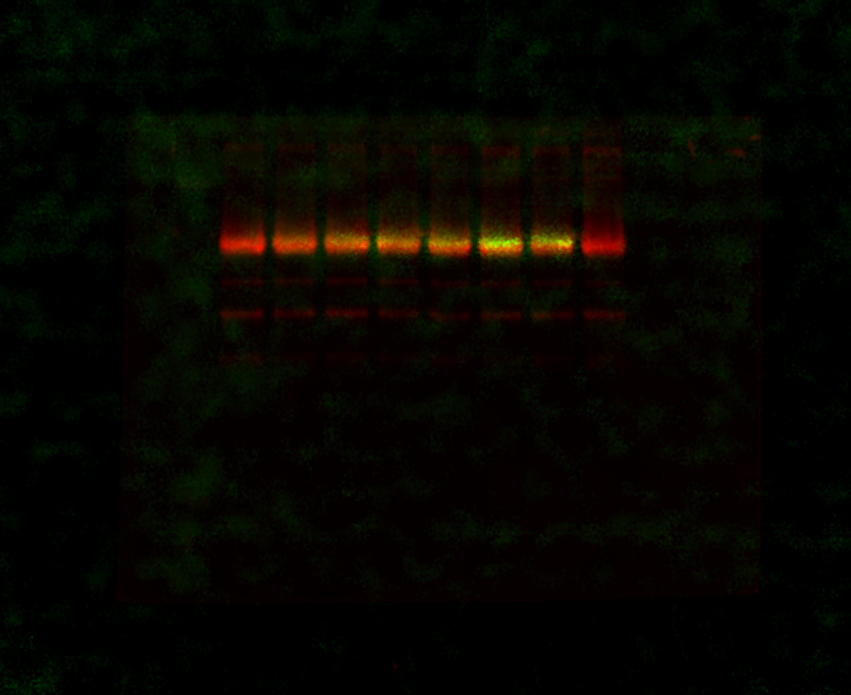

Supplement: Figure 1—figure supplement 4—source data 1. [file elife-86090-fig1-figsupp4-data1.zip › Figure 1-figure supplement 4-source data 1/Figure 1 - figure supplement 4_rep 3_merged.tif]

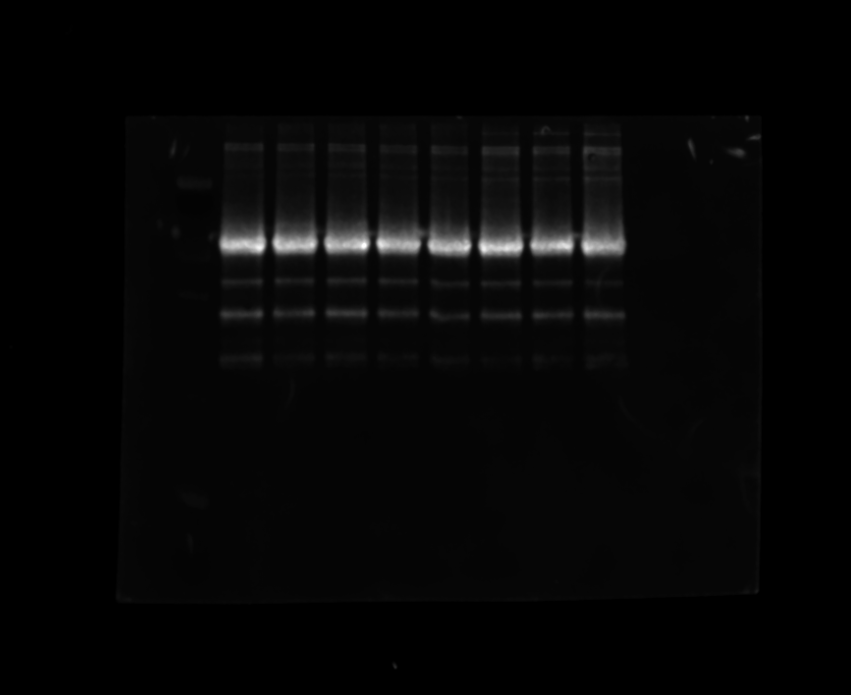

Supplement: Figure 1—figure supplement 4—source data 1. [file elife-86090-fig1-figsupp4-data1.zip › Figure 1-figure supplement 4-source data 1/Figure 1 - figure supplement 4_rep 3_pan CaMKII.tif]

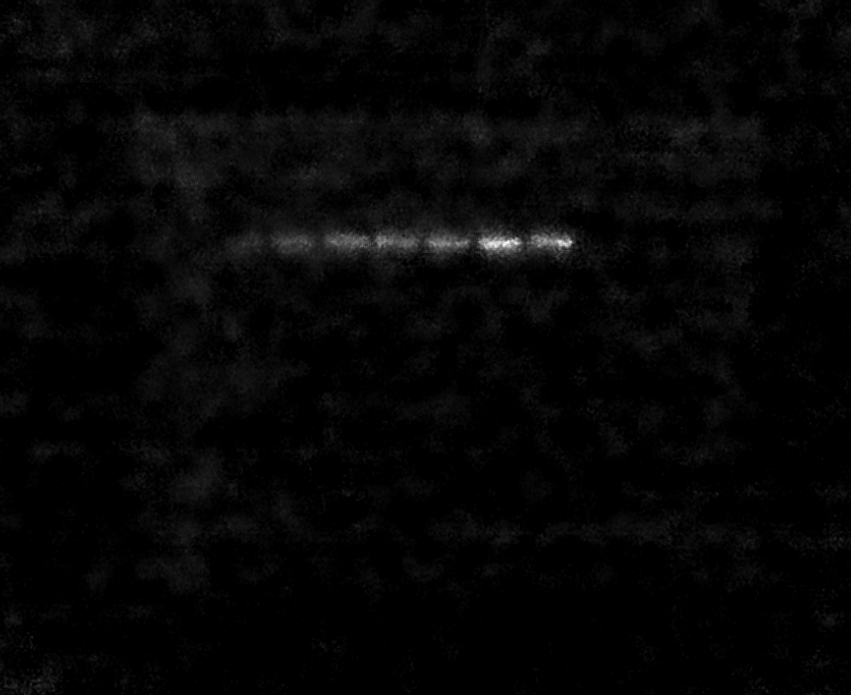

Supplement: Figure 1—figure supplement 4—source data 1. [file elife-86090-fig1-figsupp4-data1.zip › Figure 1-figure supplement 4-source data 1/Figure 1 - figure supplement 4_rep 3_pT286.tif]

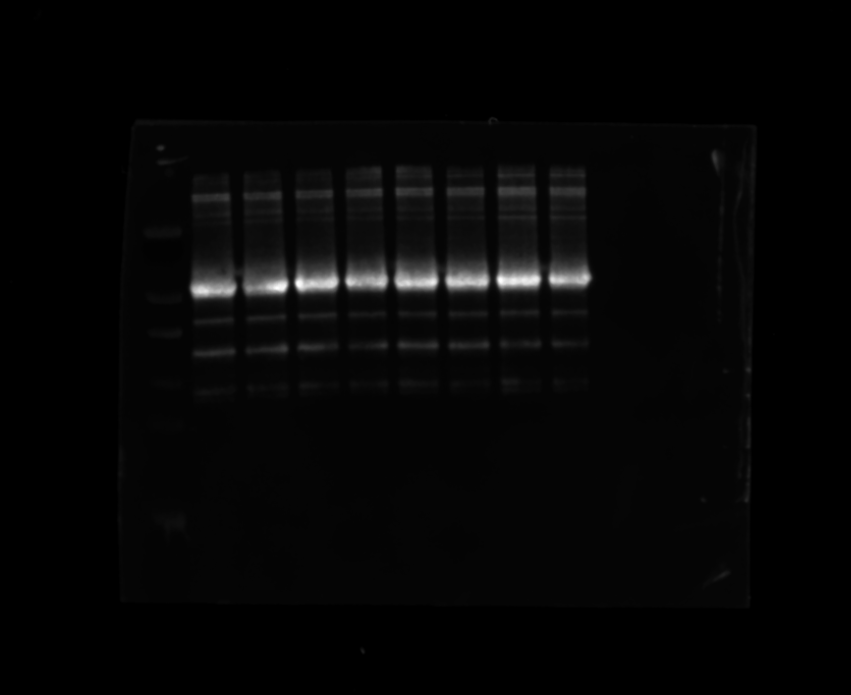

Supplement: Figure 1—figure supplement 4—source data 1. [file elife-86090-fig1-figsupp4-data1.zip › Figure 1-figure supplement 4-source data 1/Figure 1 - figure supplement 4_rep 2_pan CaMKII.tif]

Figure 1 - figure supplement 4 blots

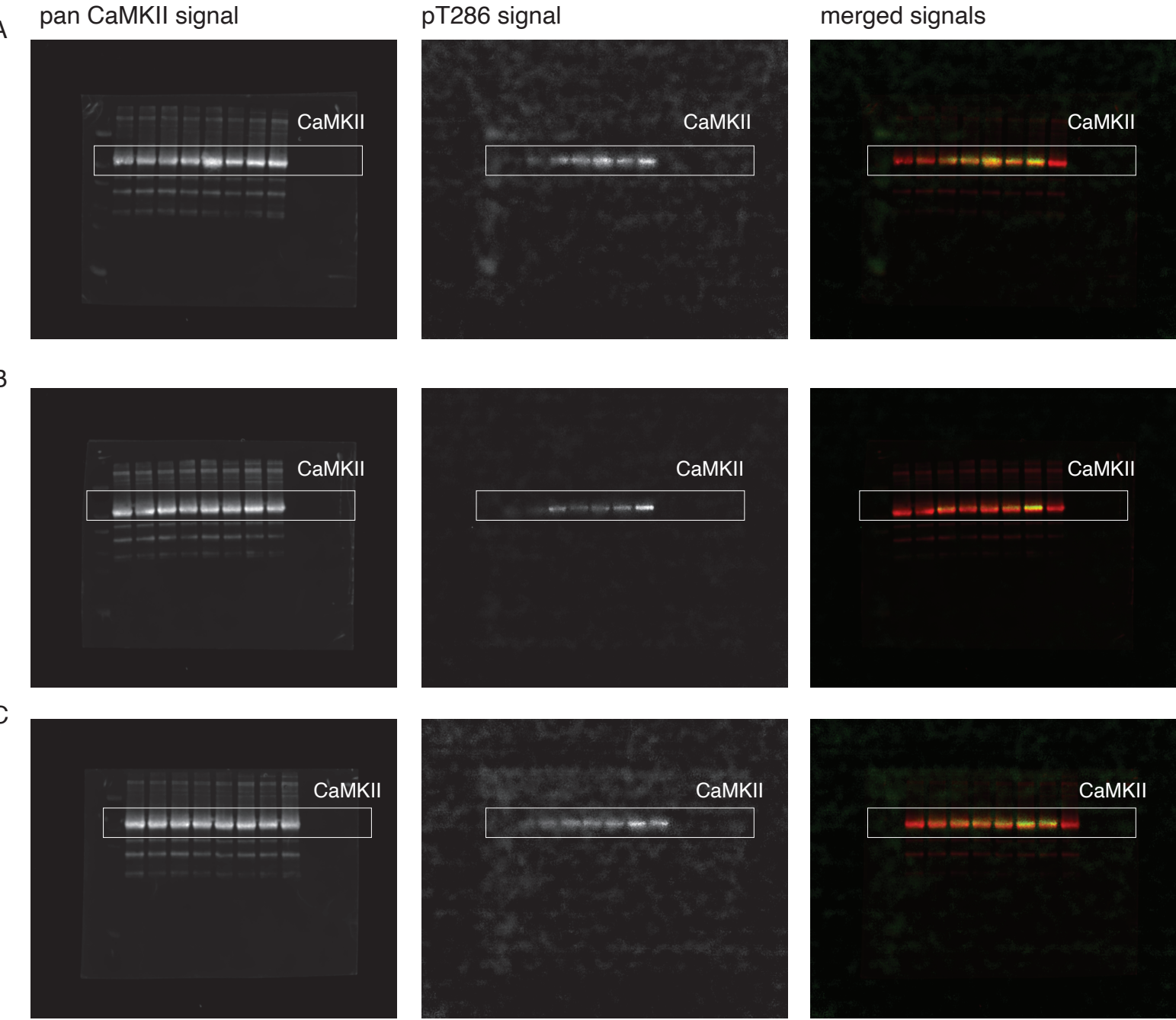

Supplement: Figure 1—figure supplement 4—source data 1. [file elife-86090-fig1-figsupp4-data1.zip › Figure 1-figure supplement 4-source data 1/Figure 1 - figure supplement 4-source data 1.pdf]

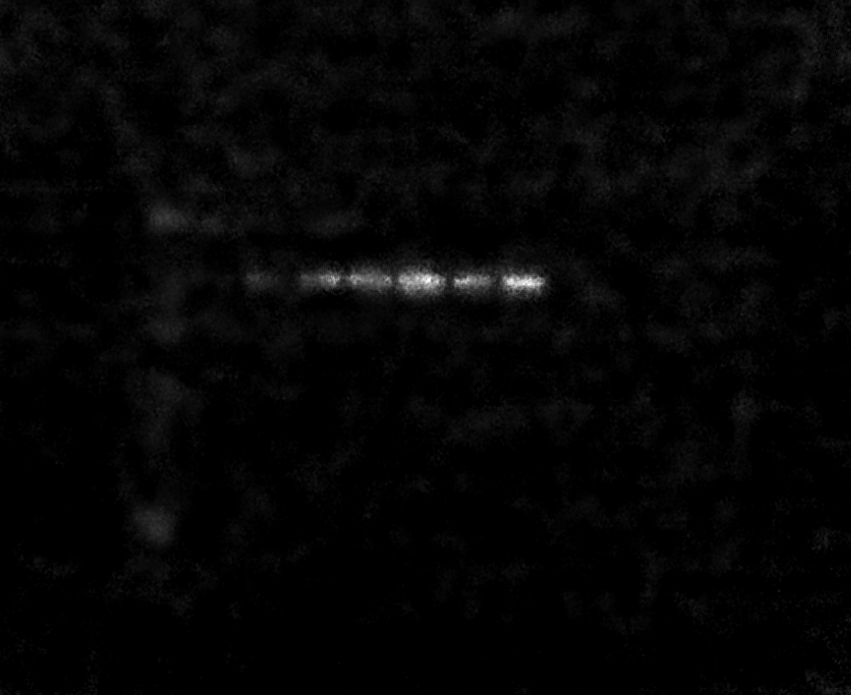

Supplement: Figure 1—figure supplement 4—source data 1. [file elife-86090-fig1-figsupp4-data1.zip › Figure 1-figure supplement 4-source data 1/Figure 1 - figure supplement 4_rep 1_pT286.tif]

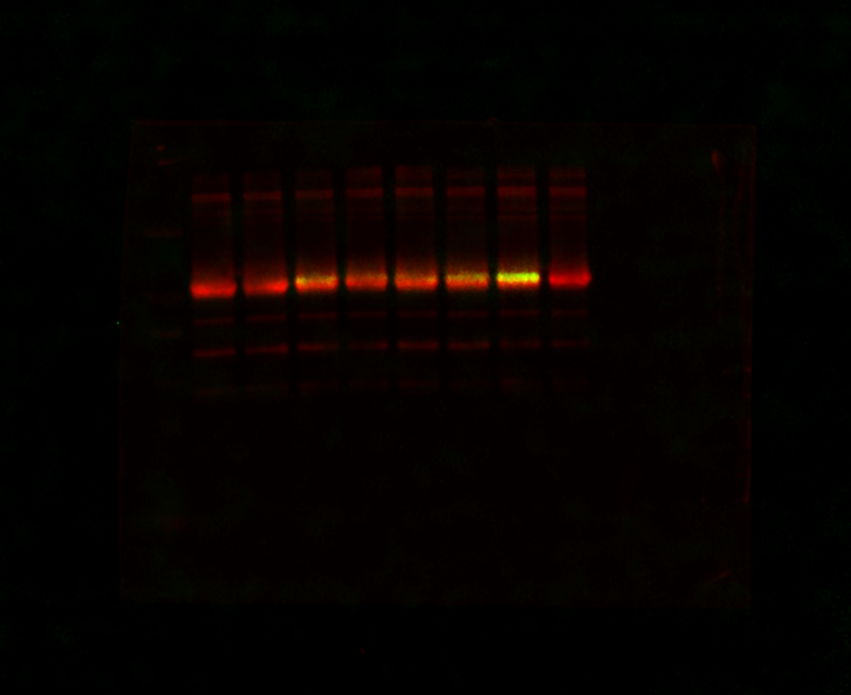

Supplement: Figure 1—figure supplement 4—source data 1. [file elife-86090-fig1-figsupp4-data1.zip › Figure 1-figure supplement 4-source data 1/Figure 1 - figure supplement 4_rep 2_merged.tif]

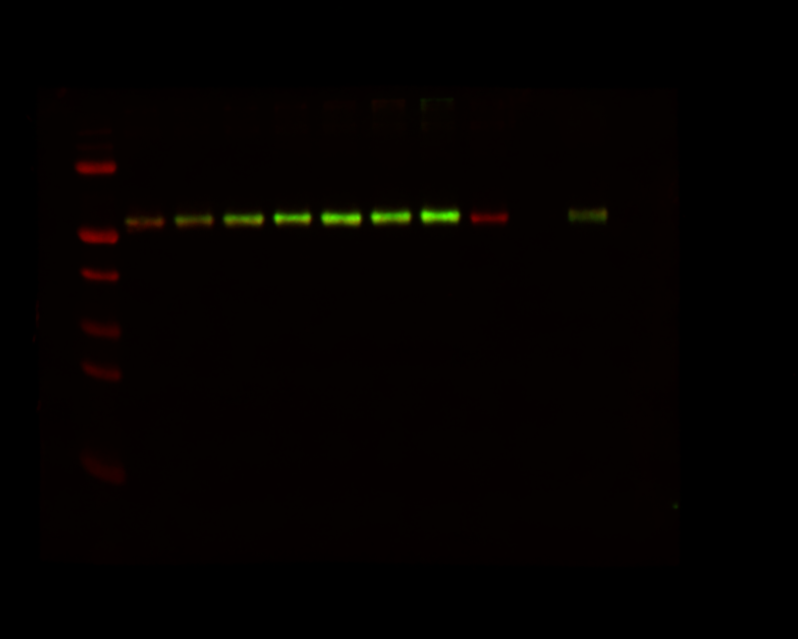

Supplement: Figure 1—figure supplement 5—source data 1. [file elife-86090-fig1-figsupp5-data1.zip › Figure 1-figure supplement 5-source data 1/Figure 1 - figure supplement 5_rep_2_merged blot.tif]

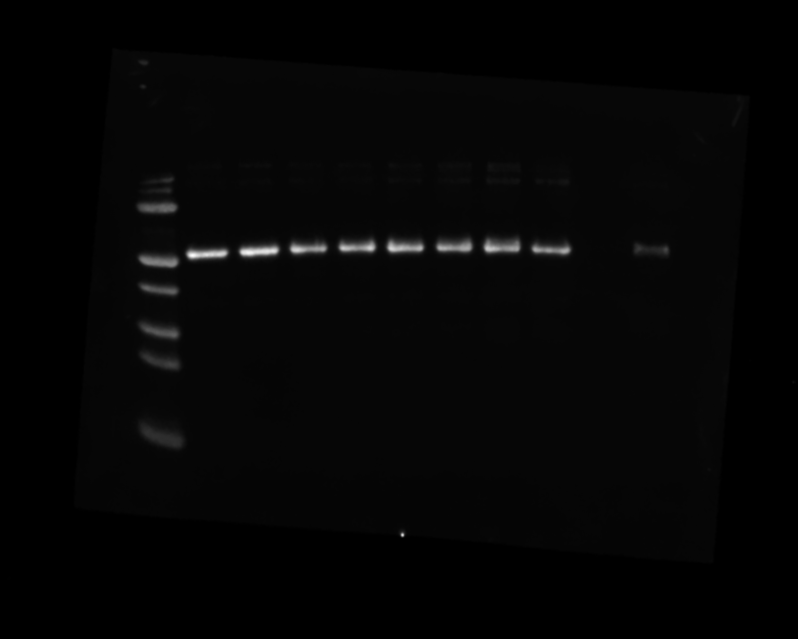

Supplement: Figure 1—figure supplement 5—source data 1. [file elife-86090-fig1-figsupp5-data1.zip › Figure 1-figure supplement 5-source data 1/Figure 1 - figure supplement 5_rep 3_pan CaMKII.tif]

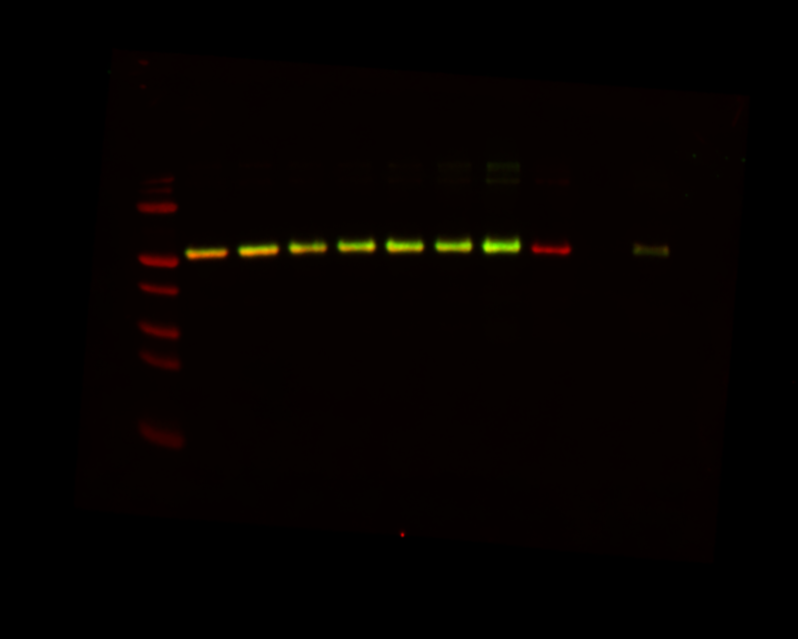

Supplement: Figure 1—figure supplement 5—source data 1. [file elife-86090-fig1-figsupp5-data1.zip › Figure 1-figure supplement 5-source data 1/Figure 1 - figure supplement 5_rep 3_merged blot.tif]

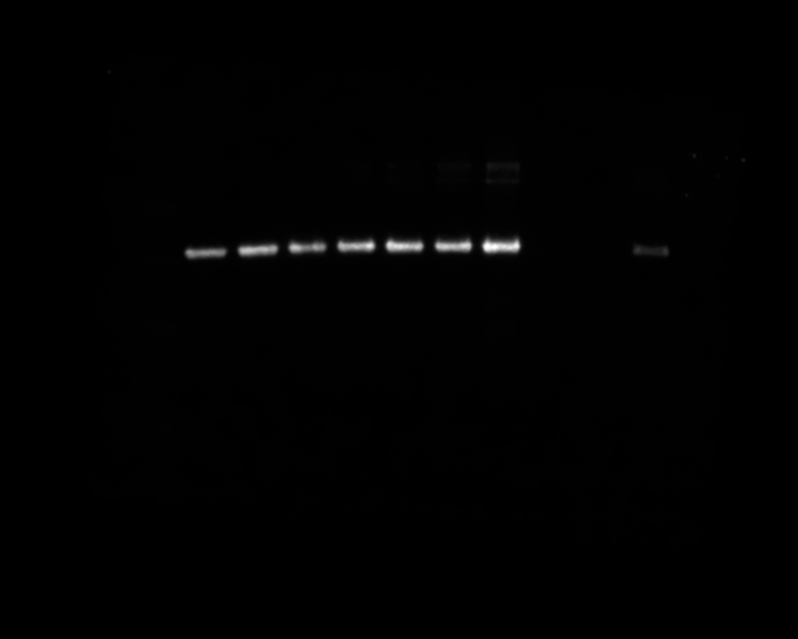

Supplement: Figure 1—figure supplement 5—source data 1. [file elife-86090-fig1-figsupp5-data1.zip › Figure 1-figure supplement 5-source data 1/Figure 1 - figure supplement 5_rep 3_pT286.tif]

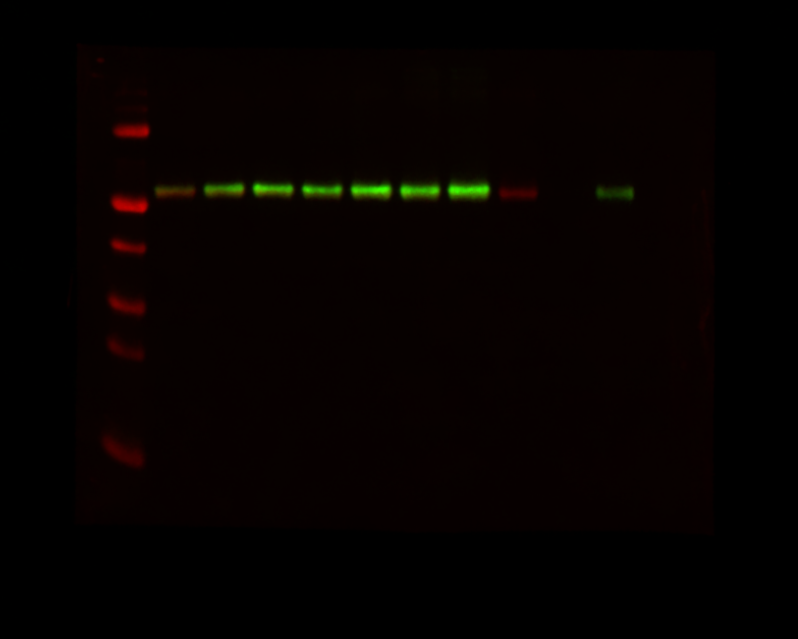

Supplement: Figure 1—figure supplement 5—source data 1. [file elife-86090-fig1-figsupp5-data1.zip › Figure 1-figure supplement 5-source data 1/Figure 1 - figure supplement 5_merged blot.tif]

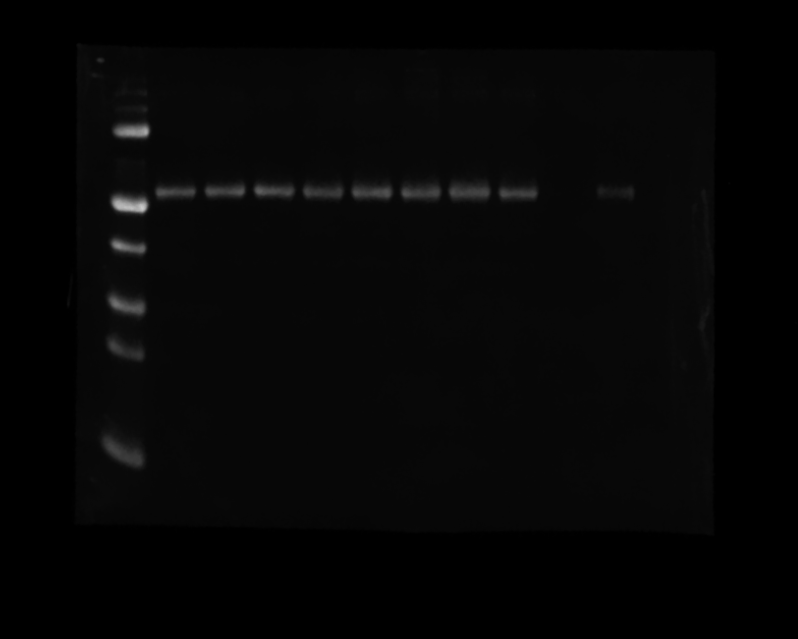

Supplement: Figure 1—figure supplement 5—source data 1. [file elife-86090-fig1-figsupp5-data1.zip › Figure 1-figure supplement 5-source data 1/Figure 1 - figure supplement 5_pan CaMKII blot.tif]

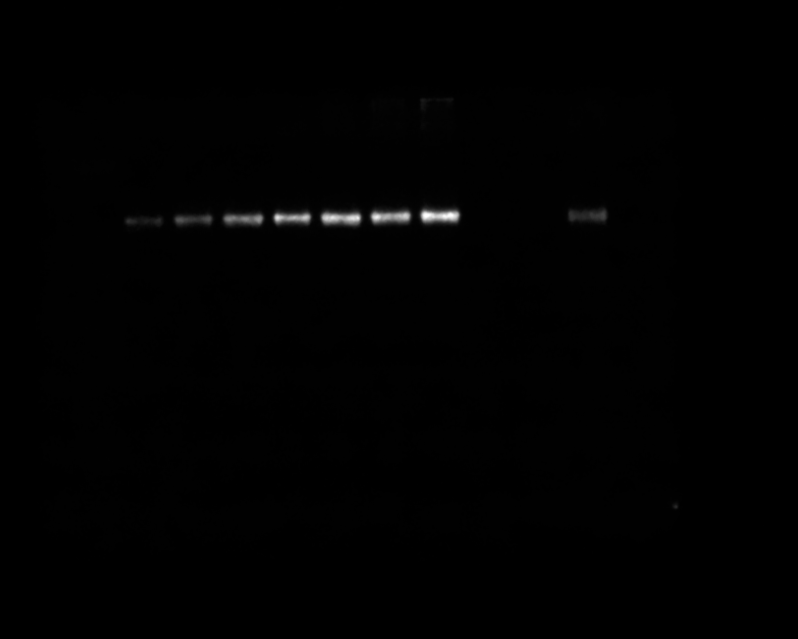

Supplement: Figure 1—figure supplement 5—source data 1. [file elife-86090-fig1-figsupp5-data1.zip › Figure 1-figure supplement 5-source data 1/Figure 1 - figure supplement 5_rep_2_pT286.tif]

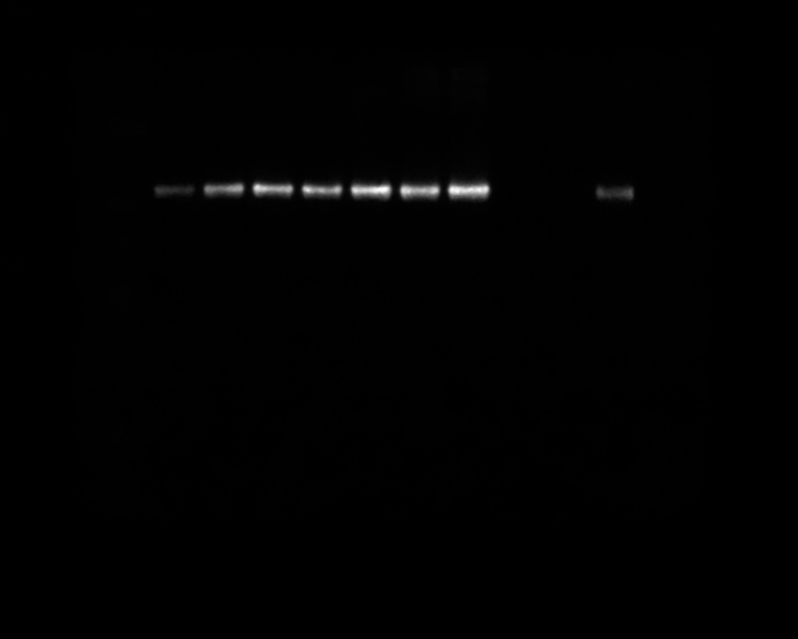

Supplement: Figure 1—figure supplement 5—source data 1. [file elife-86090-fig1-figsupp5-data1.zip › Figure 1-figure supplement 5-source data 1/Figure 1 - figure supplement 5_pT286 blot.tif]

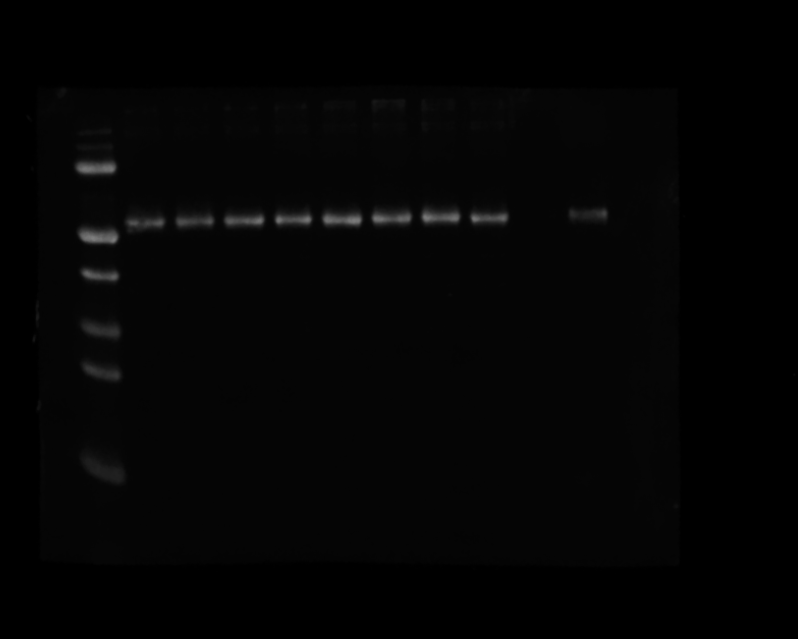

Supplement: Figure 1—figure supplement 5—source data 1. [file elife-86090-fig1-figsupp5-data1.zip › Figure 1-figure supplement 5-source data 1/Figure 1 - figure supplement 5_rep_2_pan CaMKII.tif]

Figure 1 - figure supplement 5 blots

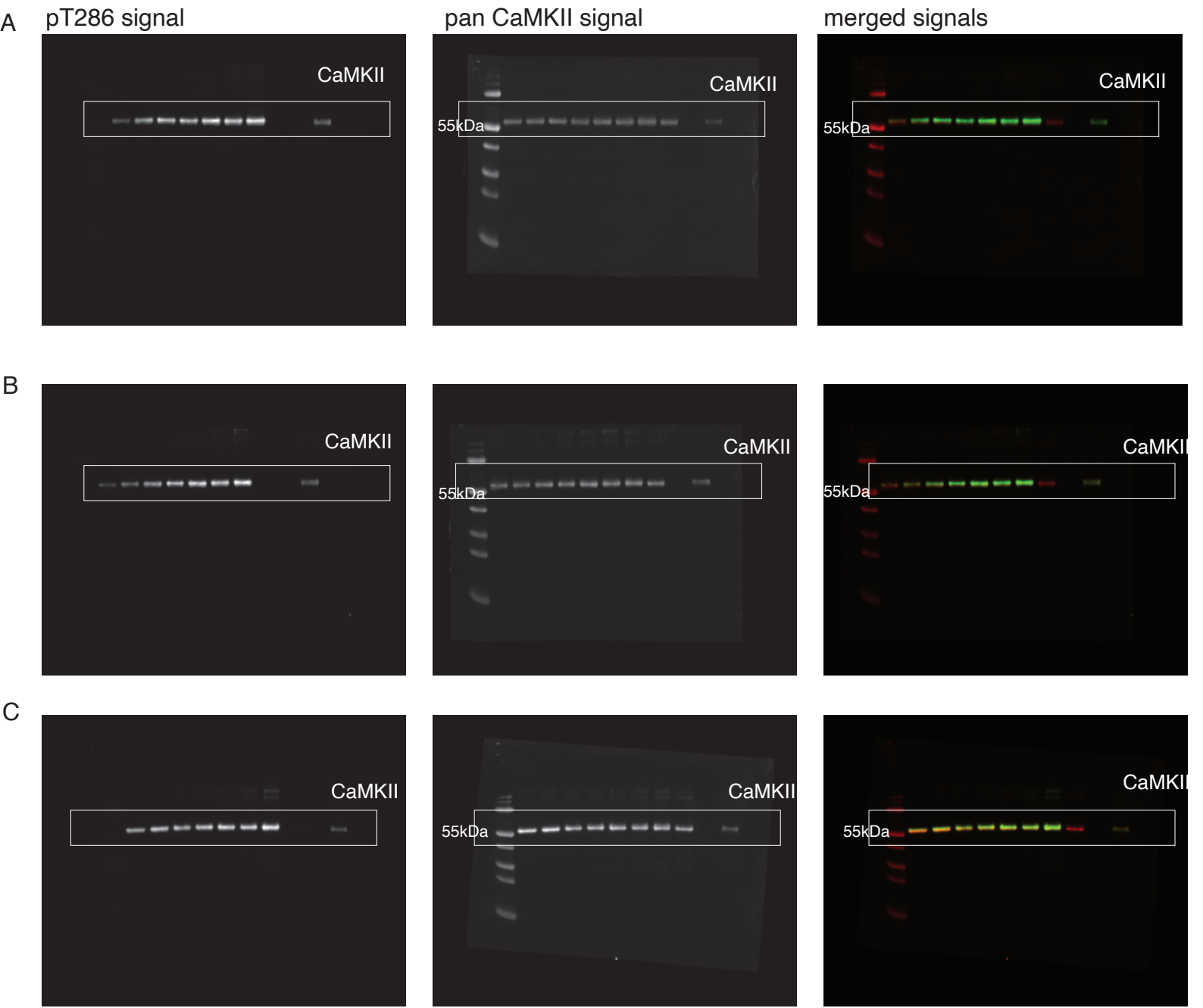

Supplement: Figure 1—figure supplement 5—source data 1. [file elife-86090-fig1-figsupp5-data1.zip › Figure 1-figure supplement 5-source data 1/Figure 1 - figure supplement 5-source data 1.pdf]

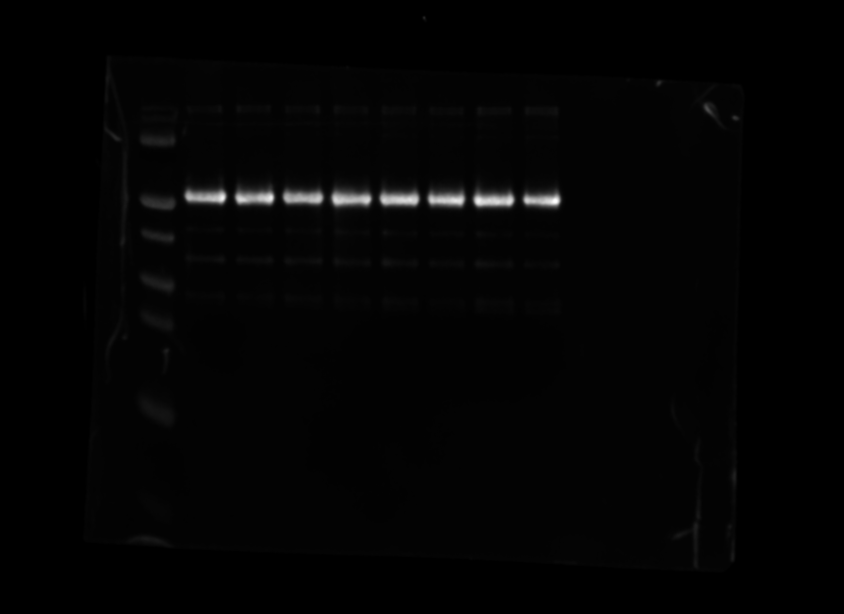

Supplement: Figure 1—figure supplement 6—source data 1. [file elife-86090-fig1-figsupp6-data1.zip › Figure 1-figure supplement 6-source data 1/Figure 1 - figure supplement 6 C pan CaMKII.tif]

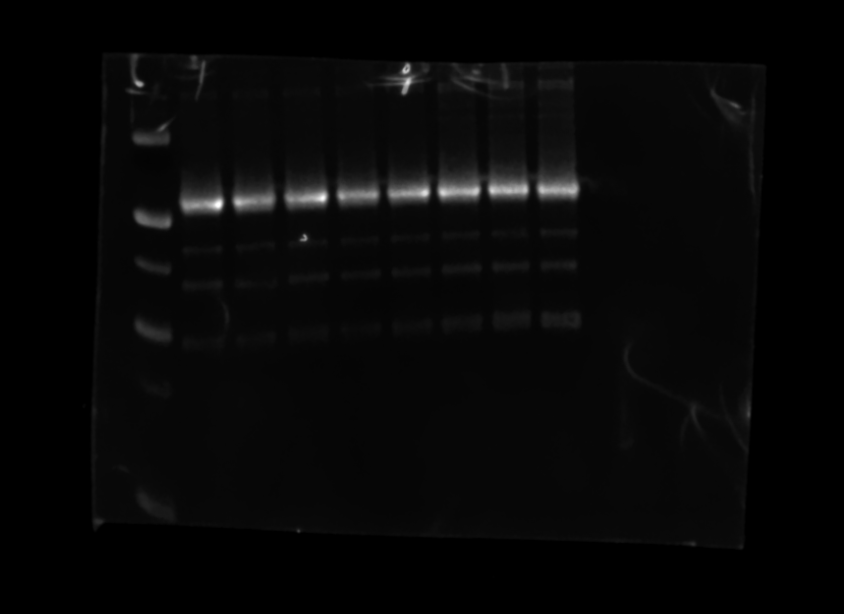

Supplement: Figure 1—figure supplement 6—source data 1. [file elife-86090-fig1-figsupp6-data1.zip › Figure 1-figure supplement 6-source data 1/Figure 1 - figure supplement 6 B pan CaMKII.tif]

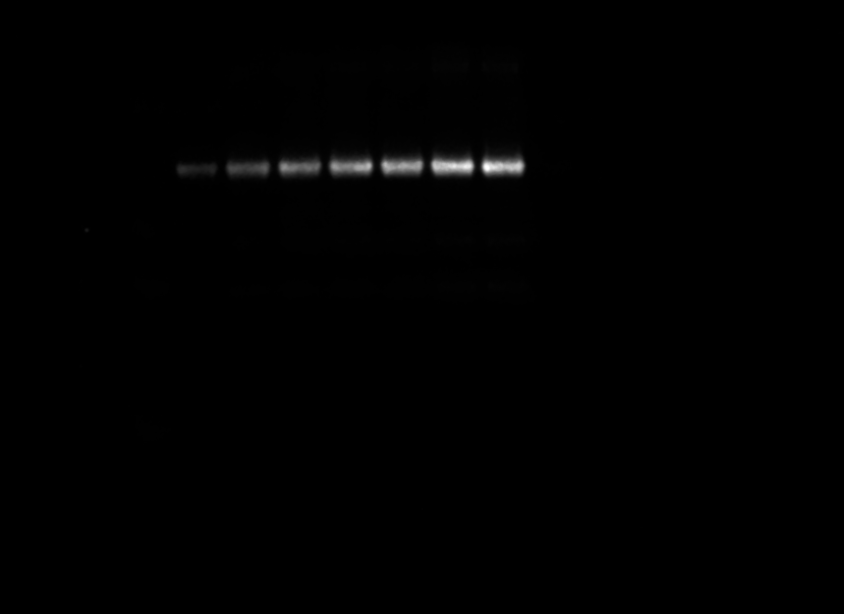

Supplement: Figure 1—figure supplement 6—source data 1. [file elife-86090-fig1-figsupp6-data1.zip › Figure 1-figure supplement 6-source data 1/Figure 1 - figure supplement 6 A pT286.tif]

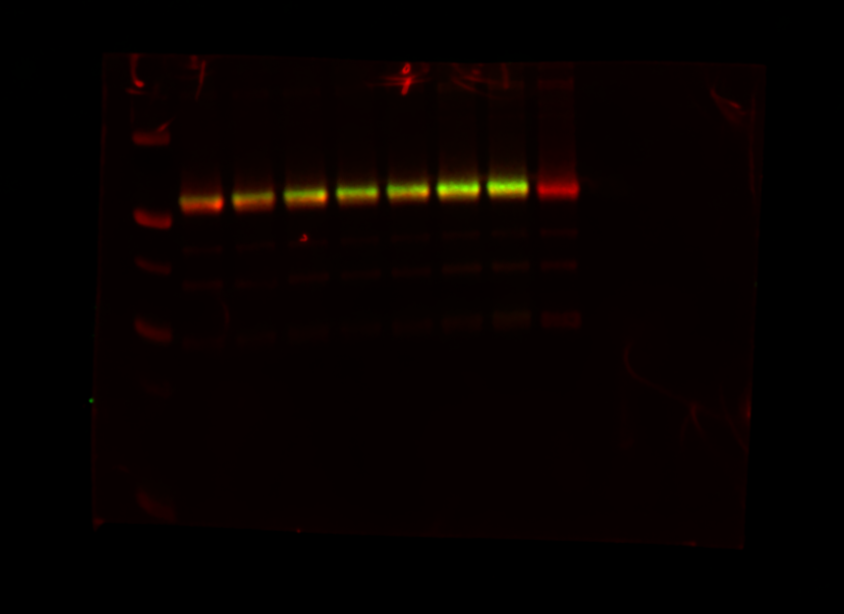

Supplement: Figure 1—figure supplement 6—source data 1. [file elife-86090-fig1-figsupp6-data1.zip › Figure 1-figure supplement 6-source data 1/Figure 1 - figure supplement 6 B merged.tif]

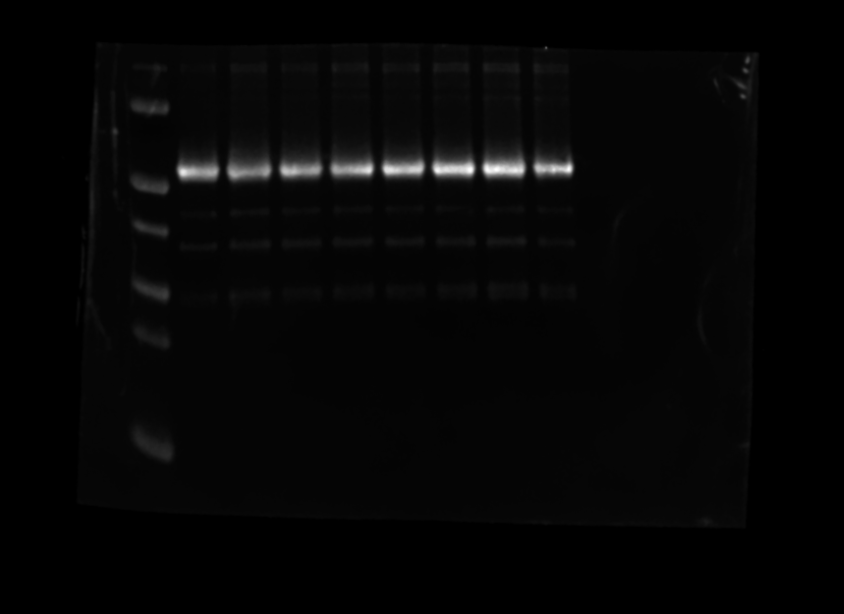

Supplement: Figure 1—figure supplement 6—source data 1. [file elife-86090-fig1-figsupp6-data1.zip › Figure 1-figure supplement 6-source data 1/Figure 1 - figure supplement 6 A pan CaMKII.tif]

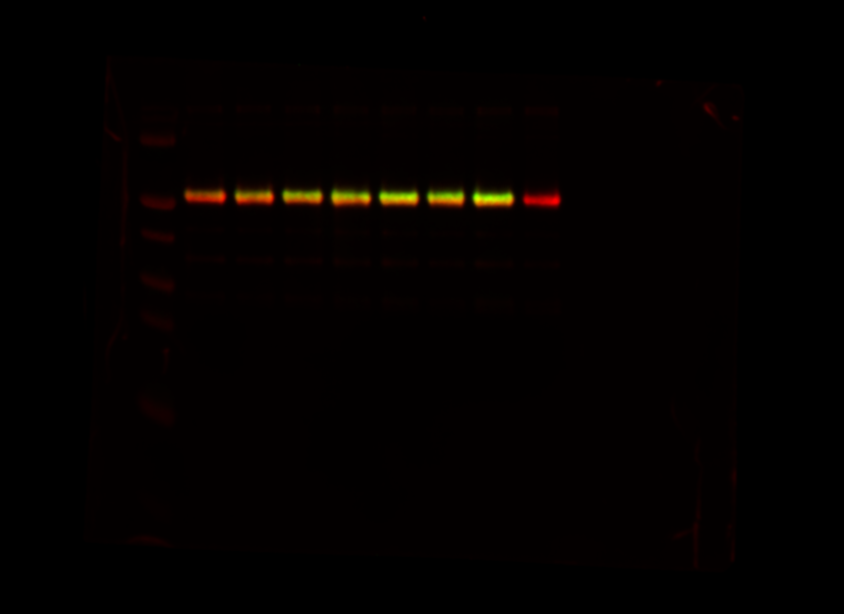

Supplement: Figure 1—figure supplement 6—source data 1. [file elife-86090-fig1-figsupp6-data1.zip › Figure 1-figure supplement 6-source data 1/Figure 1 - figure supplement 6 C merged.tif]

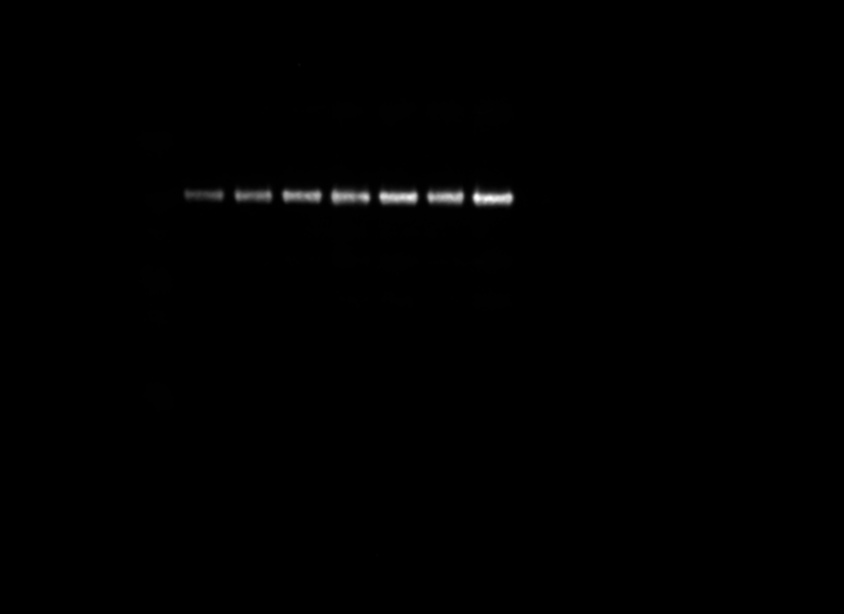

Supplement: Figure 1—figure supplement 6—source data 1. [file elife-86090-fig1-figsupp6-data1.zip › Figure 1-figure supplement 6-source data 1/Figure 1 - figure supplement 6 C pT286.tif]

Figure 1-figure supplement 6 blots

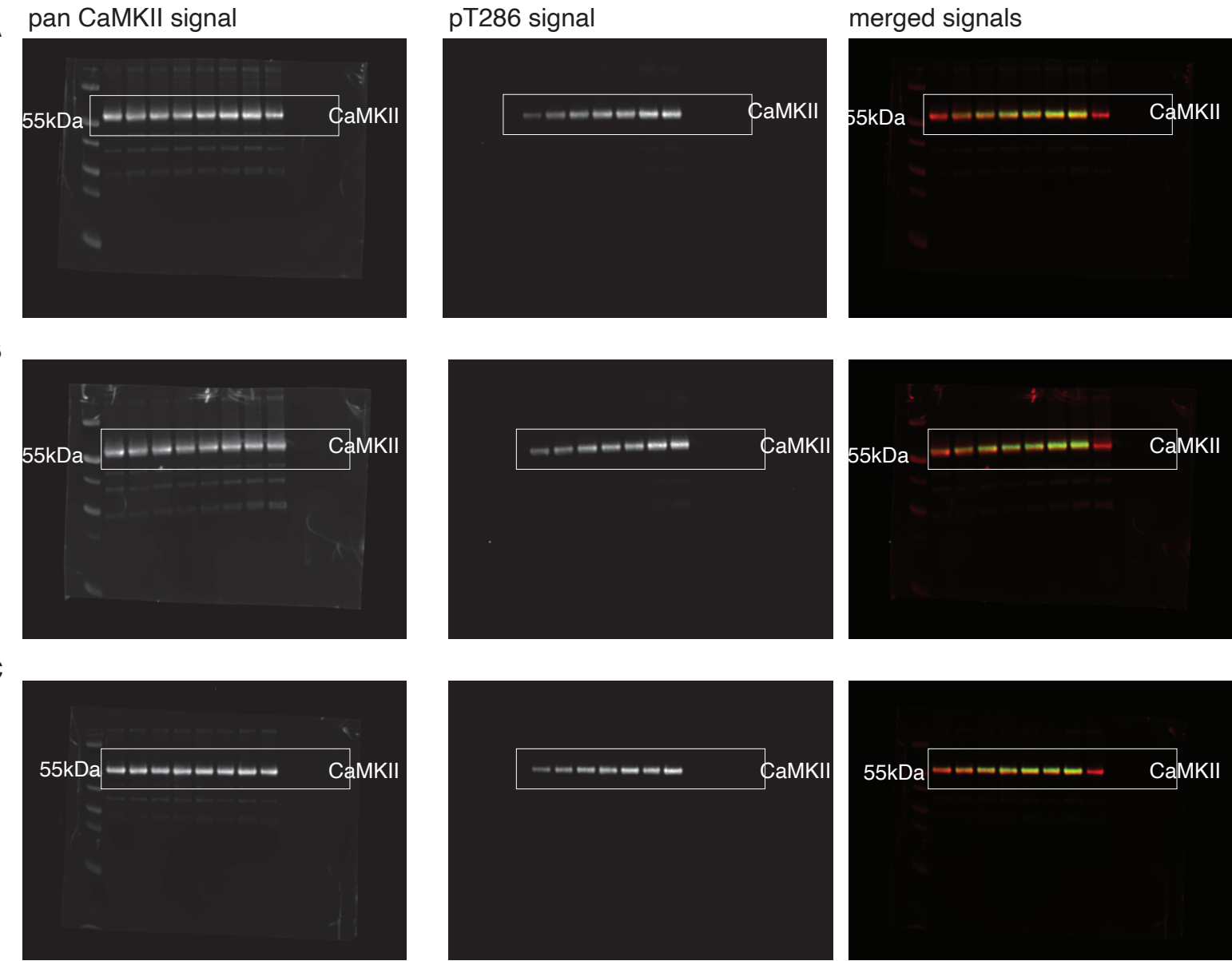

Supplement: Figure 1—figure supplement 6—source data 1. [file elife-86090-fig1-figsupp6-data1.zip › Figure 1-figure supplement 6-source data 1/Figure 1 - figure supplemet 6-source data 1.pdf]

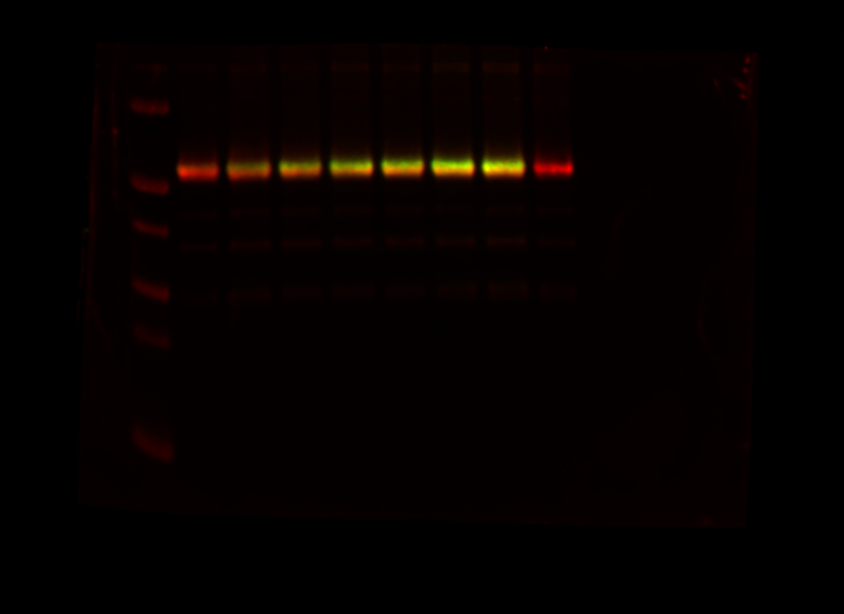

Supplement: Figure 1—figure supplement 6—source data 1. [file elife-86090-fig1-figsupp6-data1.zip › Figure 1-figure supplement 6-source data 1/Figure 1 - figure supplement 6 A merged.tif]

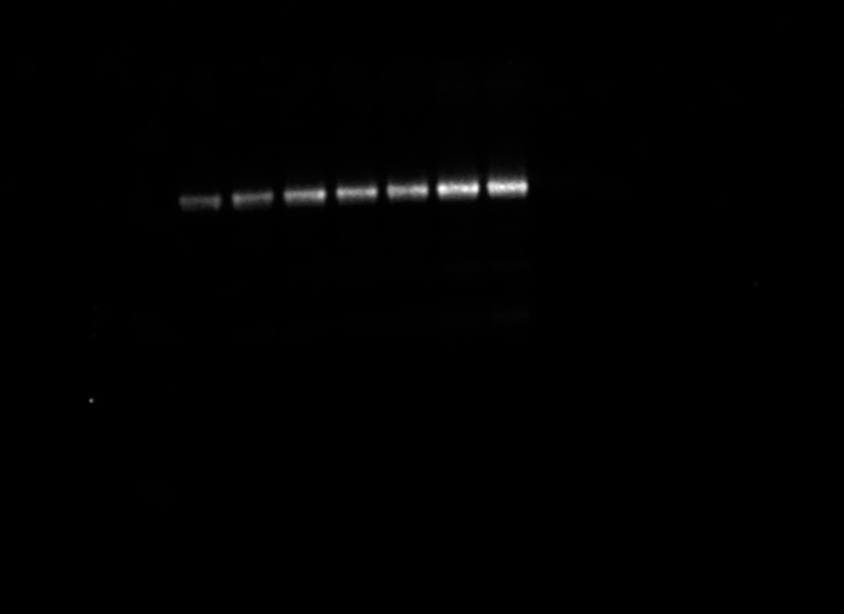

Supplement: Figure 1—figure supplement 6—source data 1. [file elife-86090-fig1-figsupp6-data1.zip › Figure 1-figure supplement 6-source data 1/Figure 1 - figure supplement 6 B pT286.tif]

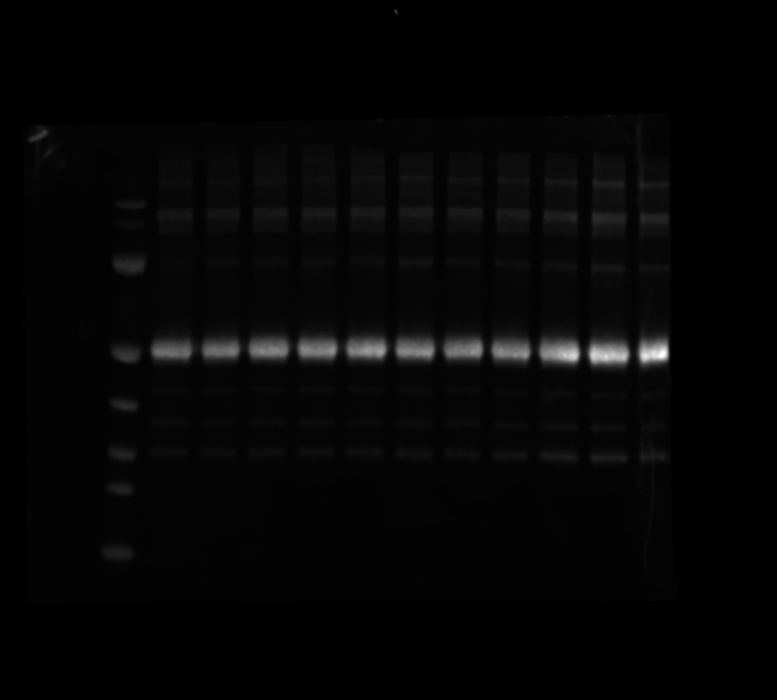

Supplement: Figure 1—figure supplement 7—source data 1. [file elife-86090-fig1-figsupp7-data1.zip › Figure 1-figure supplement 7-source data 1/Figure 1 - figure supplement 7_gel 2_ pan CaMKII.tif]

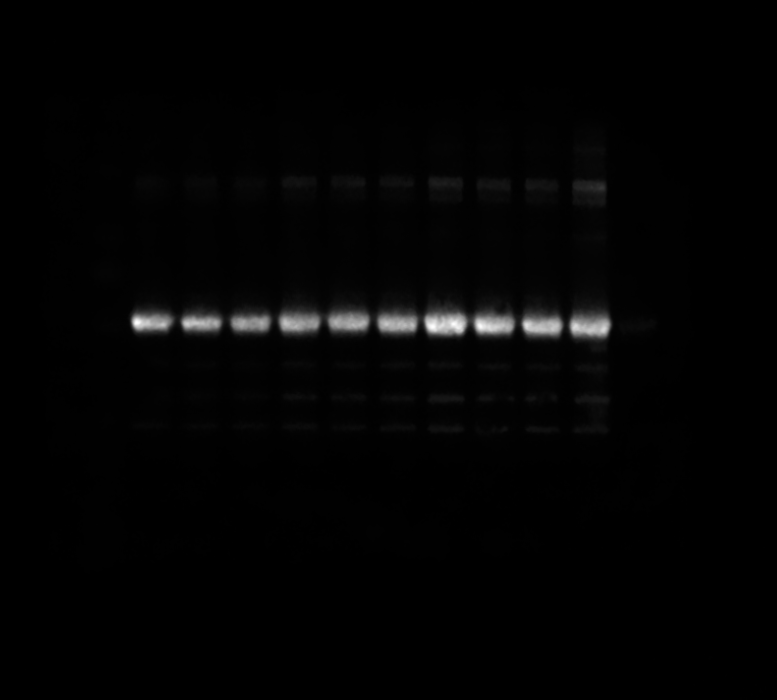

Supplement: Figure 1—figure supplement 7—source data 1. [file elife-86090-fig1-figsupp7-data1.zip › Figure 1-figure supplement 7-source data 1/Figure 1 - figure supplement 7_gel 1_ pT286.tif]

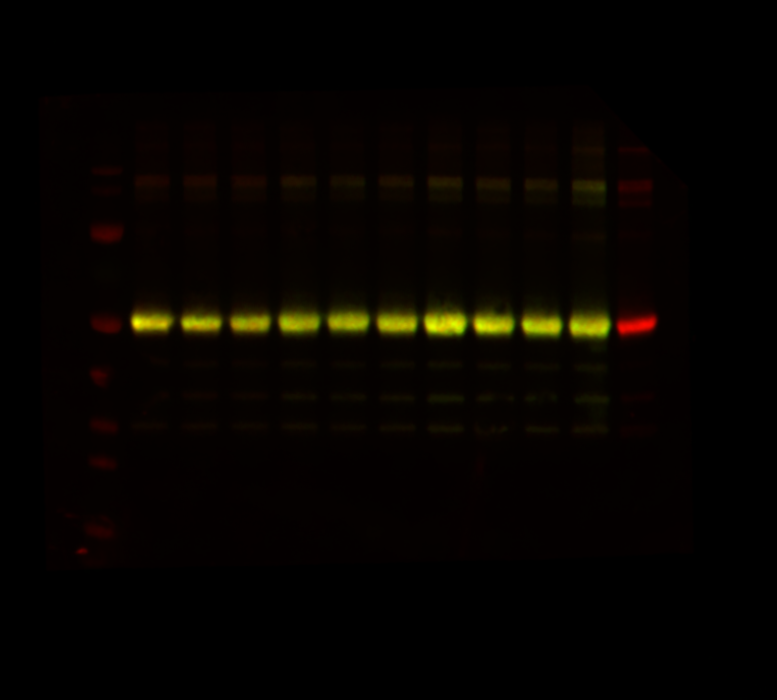

Supplement: Figure 1—figure supplement 7—source data 1. [file elife-86090-fig1-figsupp7-data1.zip › Figure 1-figure supplement 7-source data 1/Figure 1 - figure supplement 7_gel 1_ merged.tif]

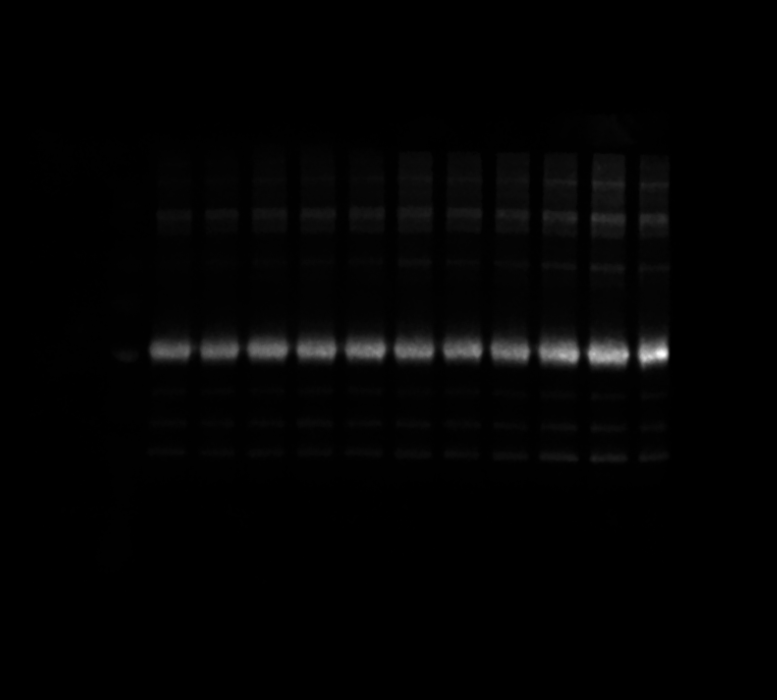

Supplement: Figure 1—figure supplement 7—source data 1. [file elife-86090-fig1-figsupp7-data1.zip › Figure 1-figure supplement 7-source data 1/Figure 1 - figure supplement 7_gel 2_ pT286.tif]

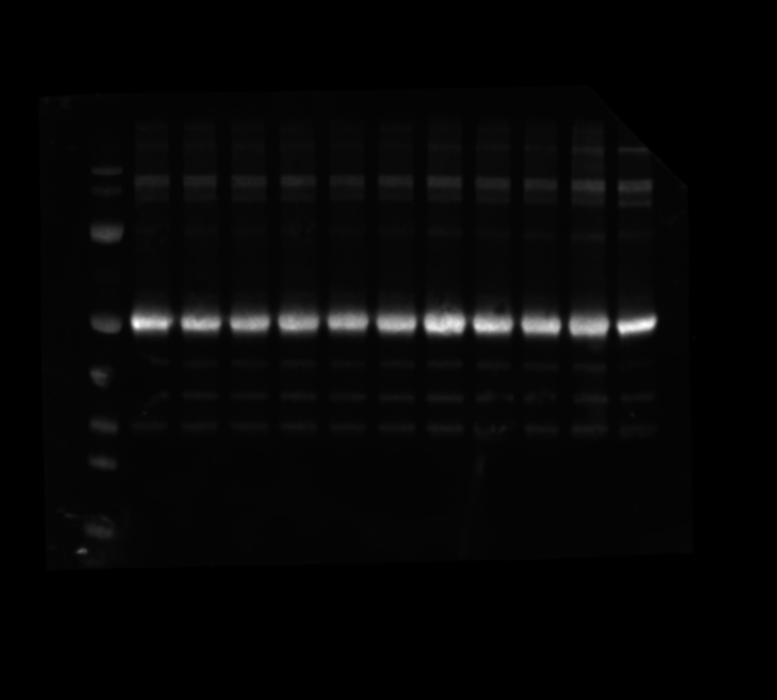

Supplement: Figure 1—figure supplement 7—source data 1. [file elife-86090-fig1-figsupp7-data1.zip › Figure 1-figure supplement 7-source data 1/Figure 1 - figure supplement 7_gel 1_ pan CaMKII.tif]

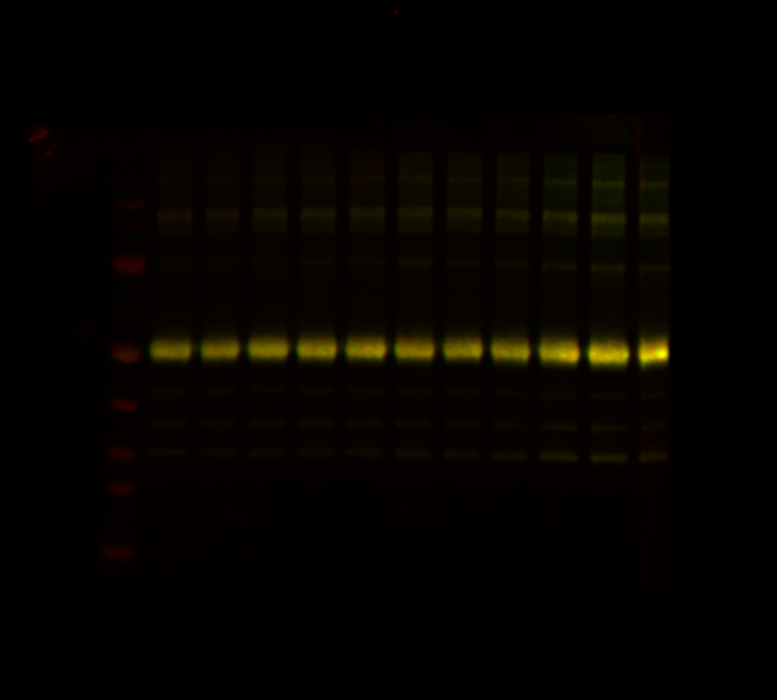

Supplement: Figure 1—figure supplement 7—source data 1. [file elife-86090-fig1-figsupp7-data1.zip › Figure 1-figure supplement 7-source data 1/Figure 1 - figure supplement 7_gel 2_ merged.tif]

Figure 1-figure supplement 7 blots

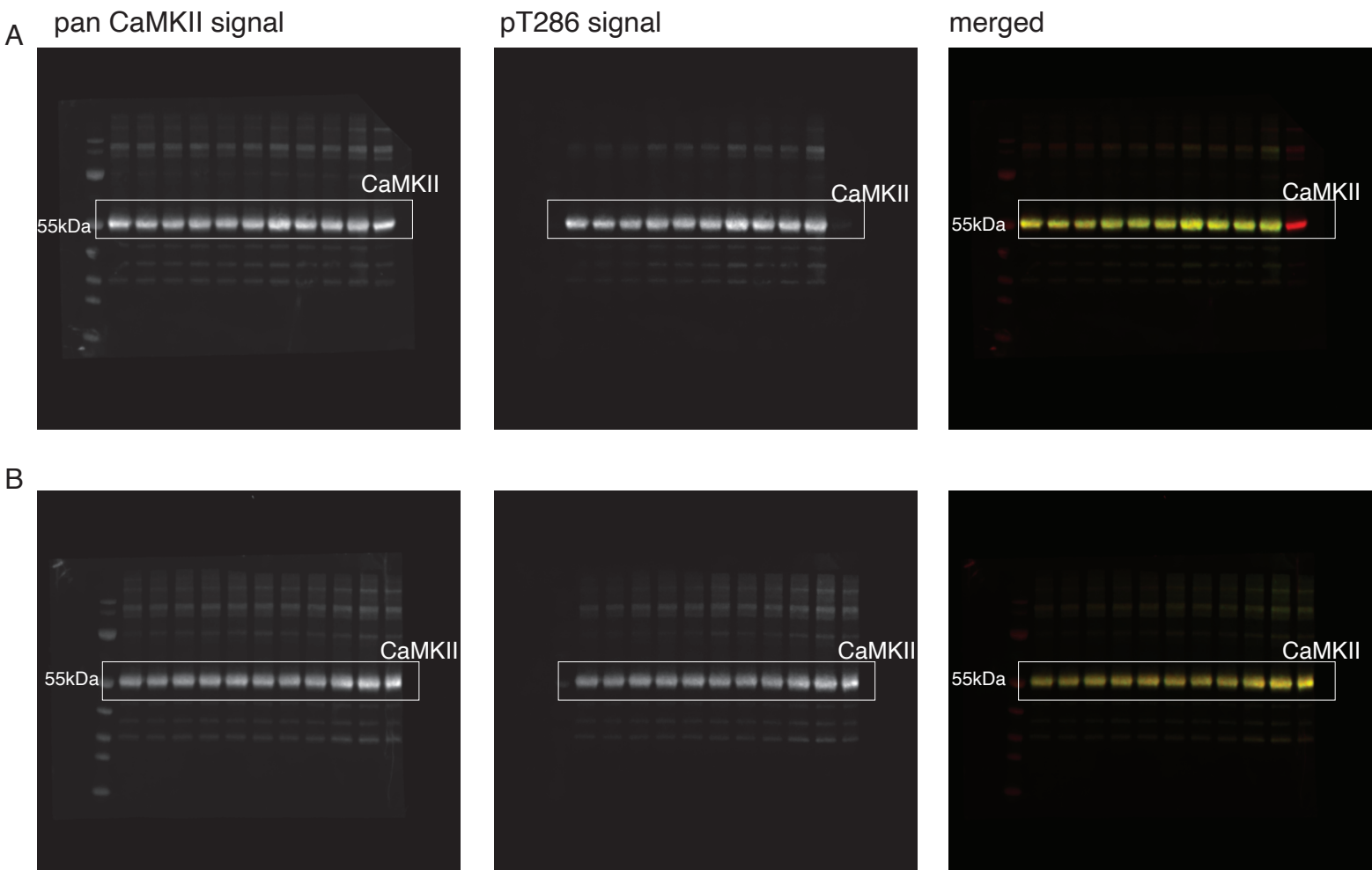

Supplement: Figure 1—figure supplement 7—source data 1. [file elife-86090-fig1-figsupp7-data1.zip › Figure 1-figure supplement 7-source data 1/Figure 1 - figure supplement 7-source data 1.pdf]

Figure 2B

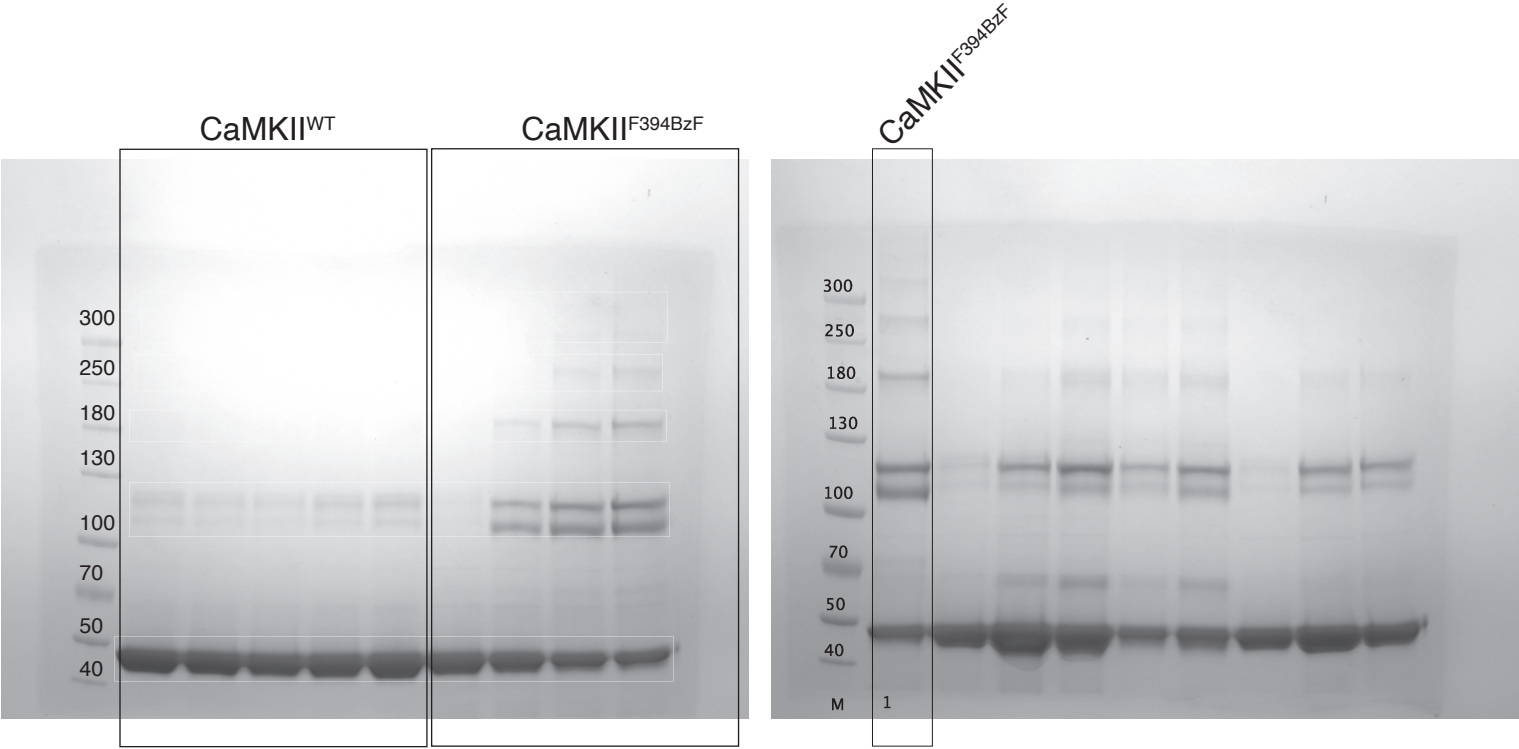

Supplement: Figure 2—source data 1. [file elife-86090-fig2-data1.zip › Figure 2-source data 1/Figure 2-source data 1.pdf]

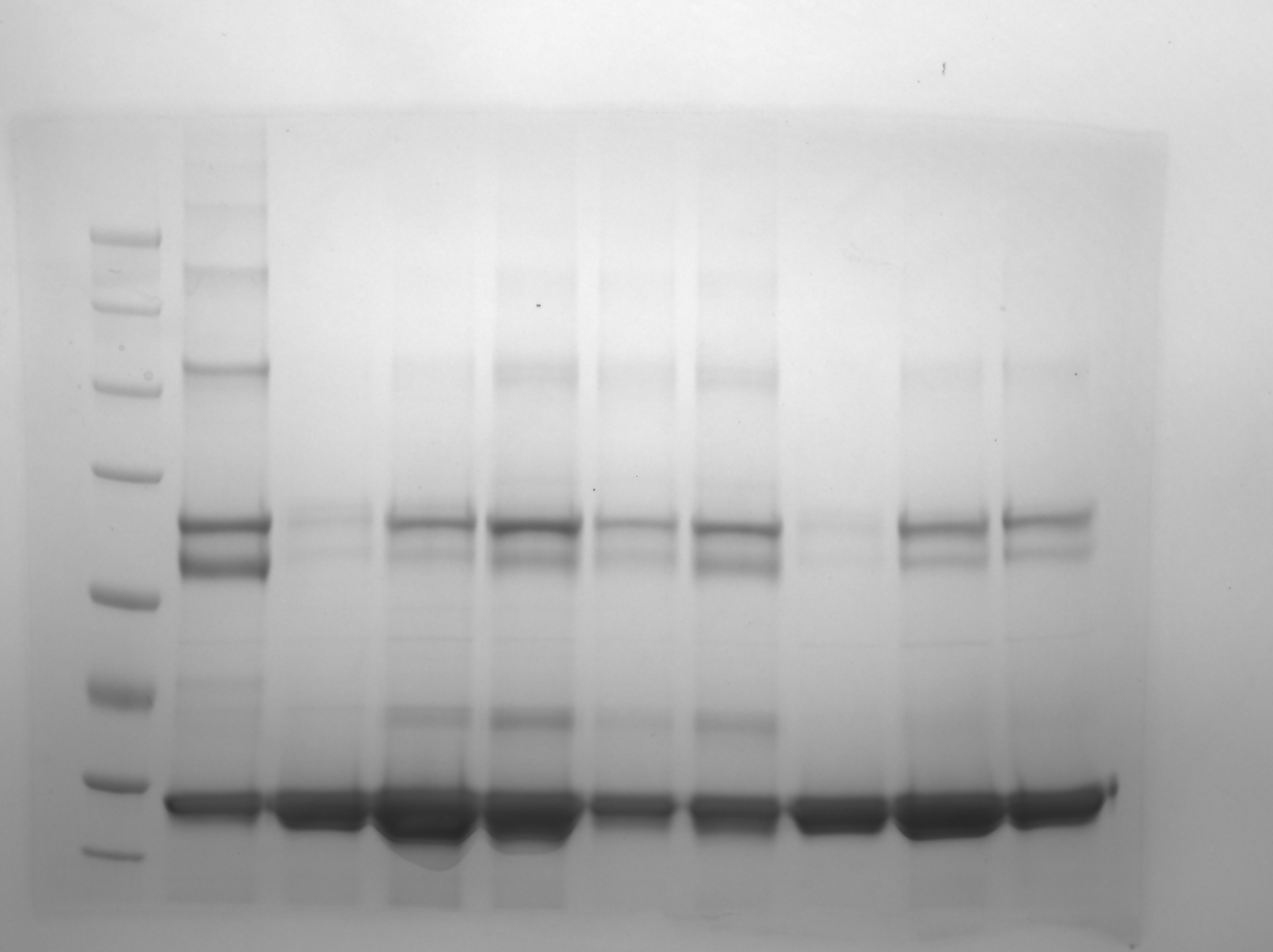

Supplement: Figure 2—source data 1. [file elife-86090-fig2-data1.zip › Figure 2-source data 1/Figure 2B gel second part.tif]

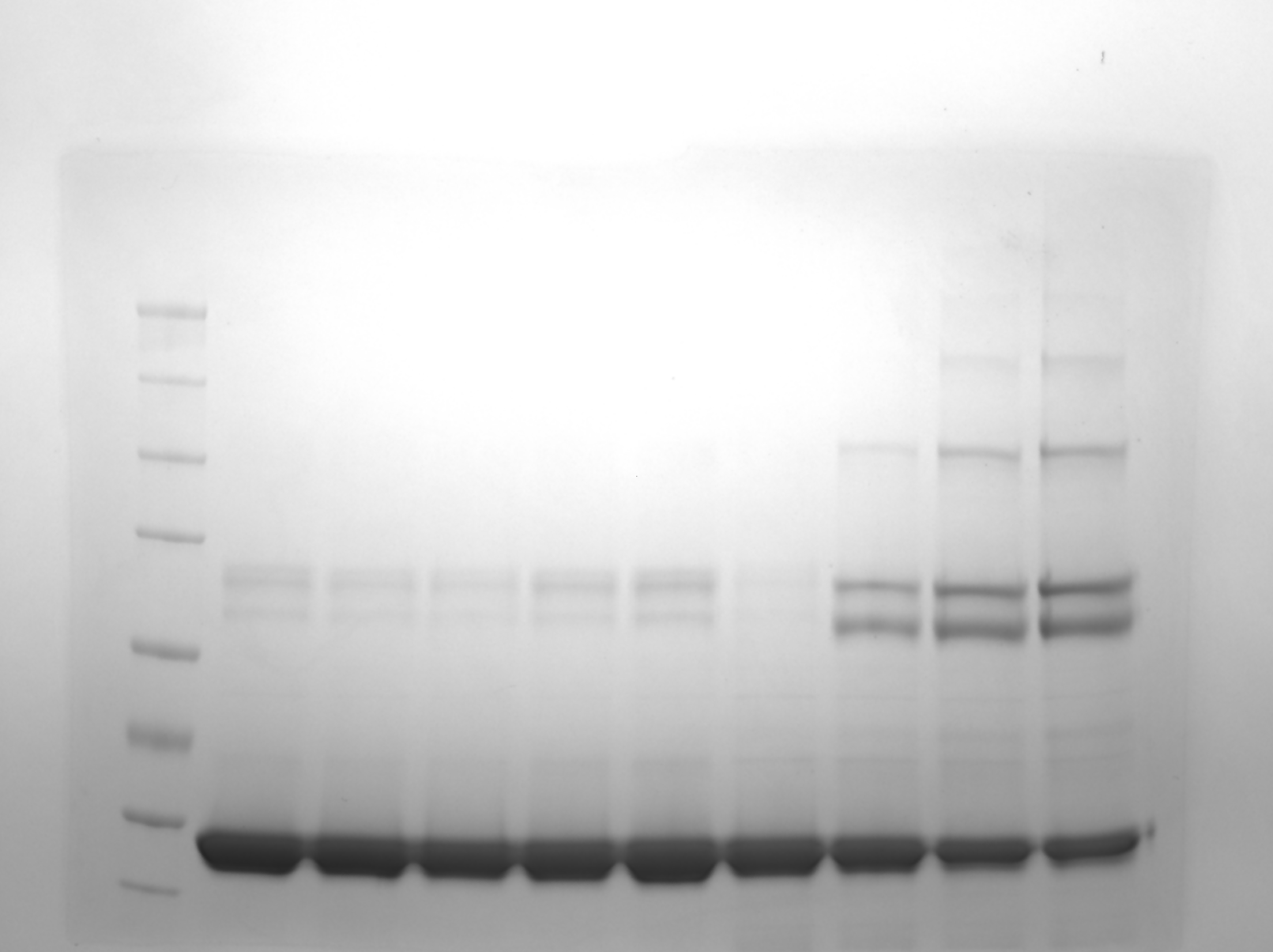

Supplement: Figure 2—source data 1. [file elife-86090-fig2-data1.zip › Figure 2-source data 1/Figure 2B gel first part.tif]

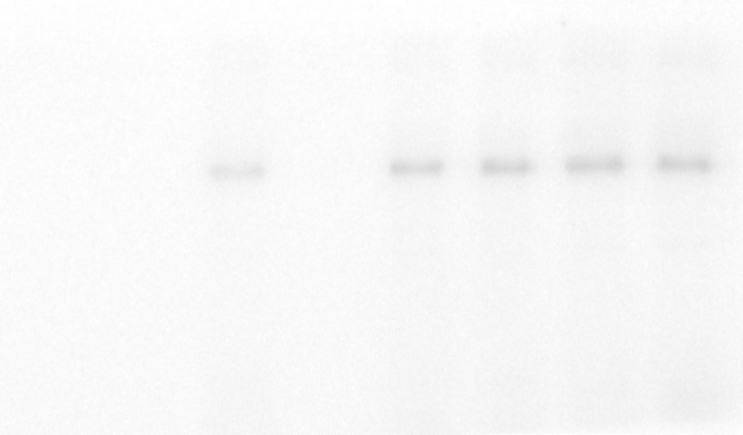

Supplement: Figure 2—figure supplement 1—source data 1. [file elife-86090-fig2-figsupp1-data1.zip › Figure 2-figure supplement 1-source data 1/Figure 2 - figure supplement 1_B_rep_3.tif]

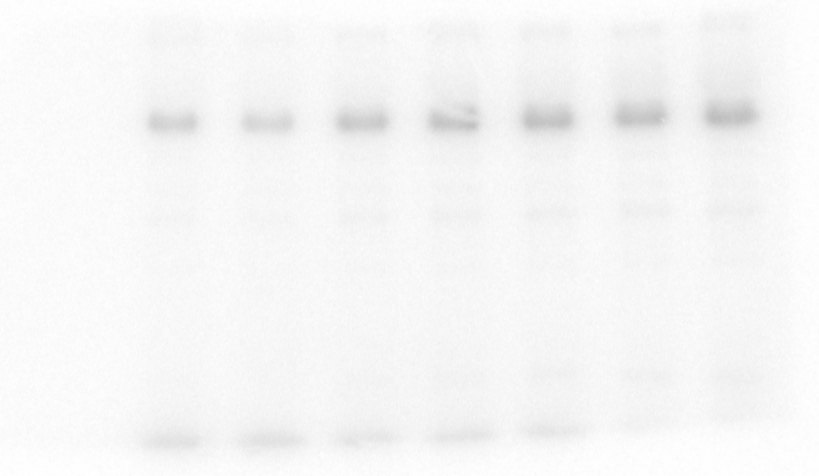

Supplement: Figure 2—figure supplement 1—source data 1. [file elife-86090-fig2-figsupp1-data1.zip › Figure 2-figure supplement 1-source data 1/Figure 2 - figure supplement 1_B_rep_2.tif]

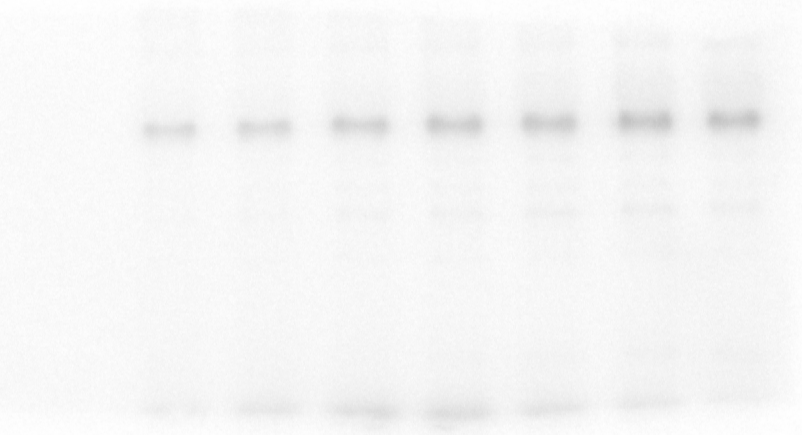

Supplement: Figure 2—figure supplement 1—source data 1. [file elife-86090-fig2-figsupp1-data1.zip › Figure 2-figure supplement 1-source data 1/Figure 2 - figure supplement 1_B_rep_1.tif]

Figure 2-figure supplement 1 gels

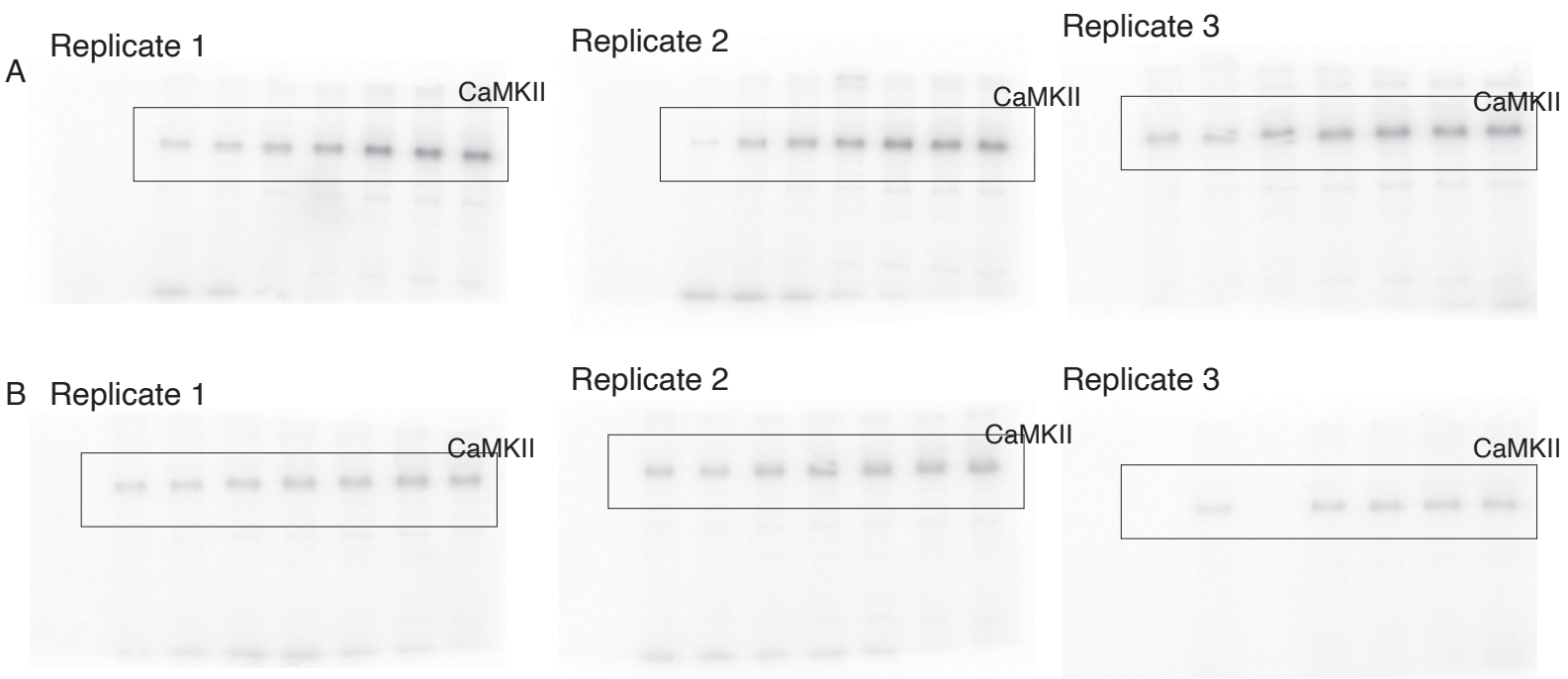

Supplement: Figure 2—figure supplement 1—source data 1. [file elife-86090-fig2-figsupp1-data1.zip › Figure 2-figure supplement 1-source data 1/Figure 2 - figure supplement 1-source data 1.pdf]

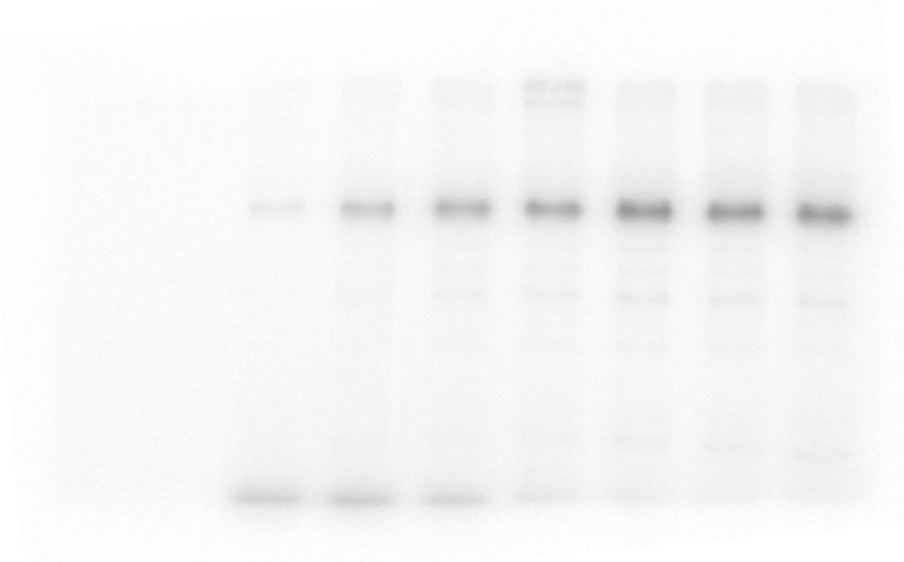

Supplement: Figure 2—figure supplement 1—source data 1. [file elife-86090-fig2-figsupp1-data1.zip › Figure 2-figure supplement 1-source data 1/Figure 2 - figure supplement 1_A_rep_2.tif]

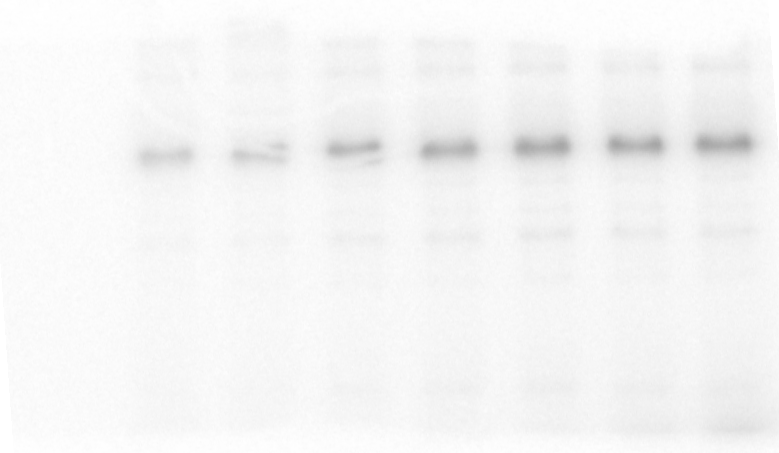

Supplement: Figure 2—figure supplement 1—source data 1. [file elife-86090-fig2-figsupp1-data1.zip › Figure 2-figure supplement 1-source data 1/Figure 2 - figure supplement 1_A_rep_3.tif]

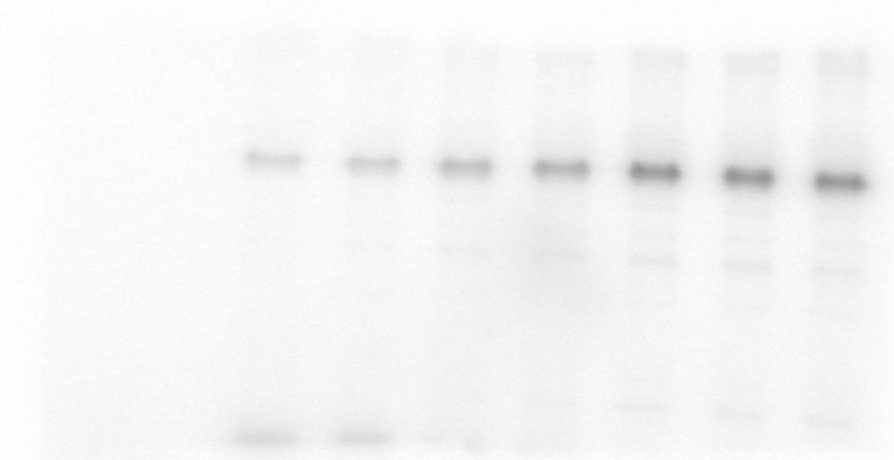

Supplement: Figure 2—figure supplement 1—source data 1. [file elife-86090-fig2-figsupp1-data1.zip › Figure 2-figure supplement 1-source data 1/Figure 2 - figure supplement 1_A_rep_1.tif]

Figure 2-figure supplement 2 gels

A

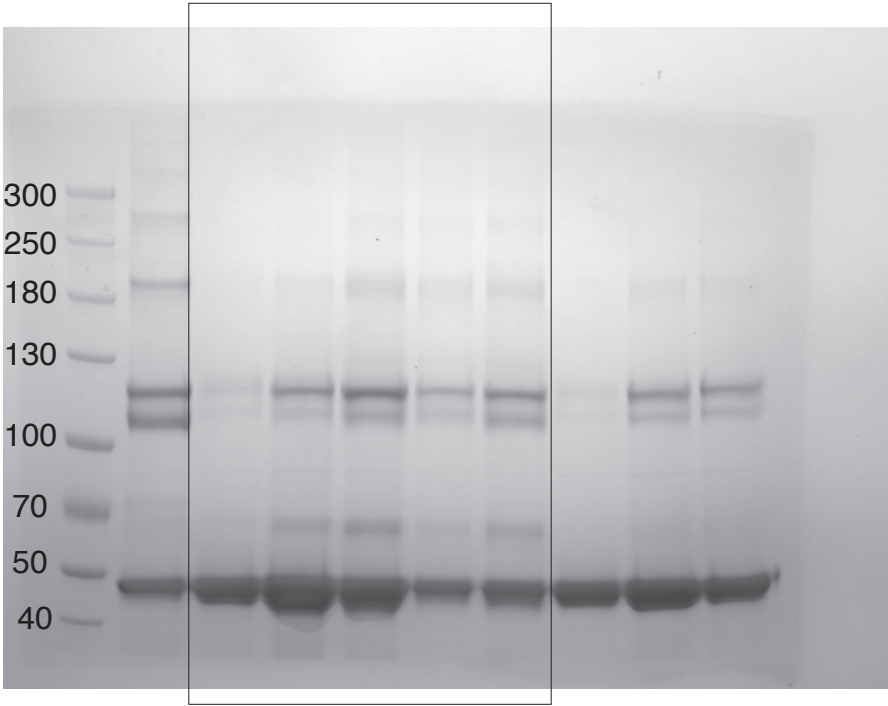

B

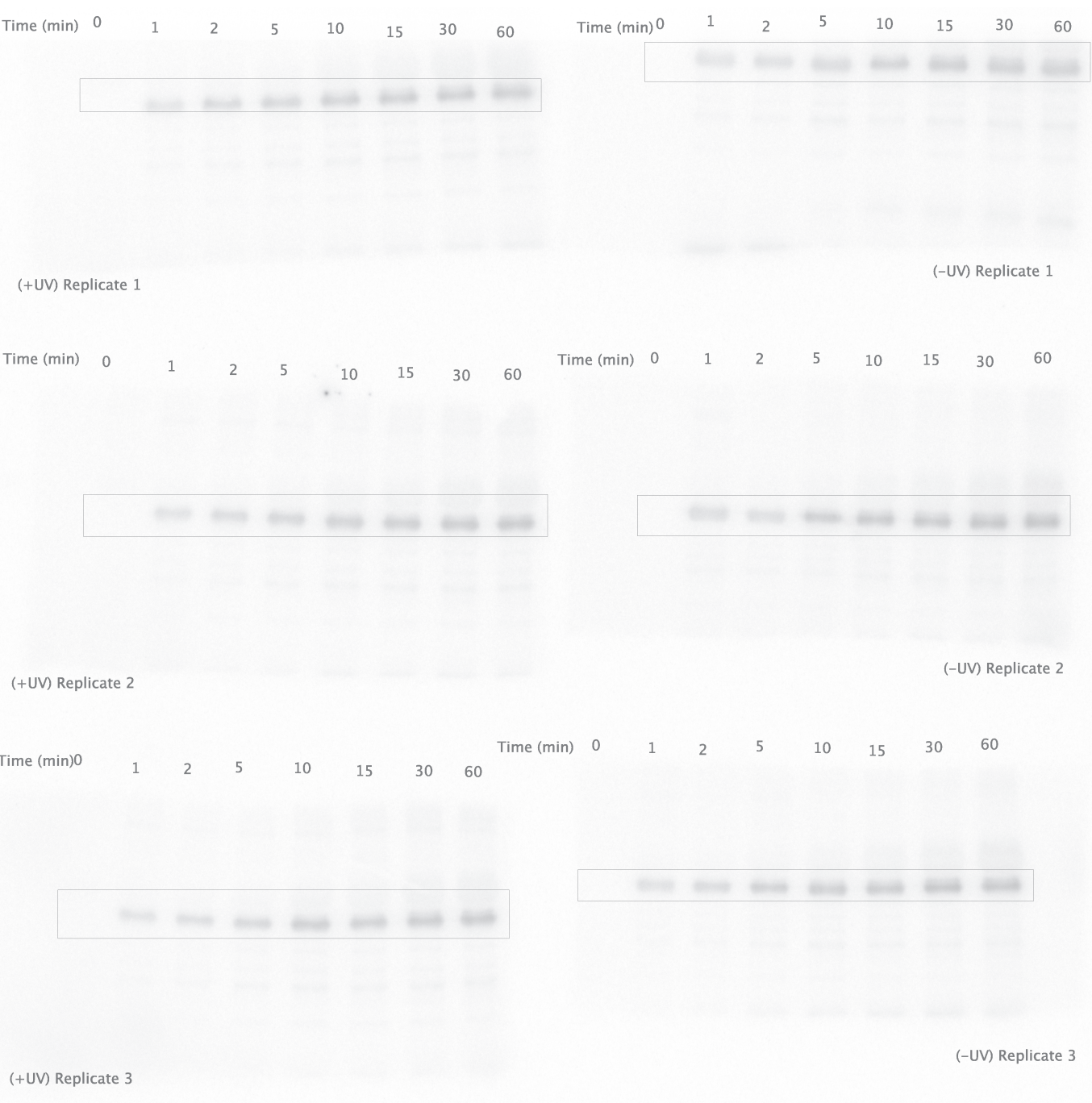

Supplement: Figure 2—figure supplement 2—source data 1. [file elife-86090-fig2-figsupp2-data1.zip › Figure 2-figure supplement 2-source data 1/Figure 2 - figure supplements 2-source data 1.pdf]

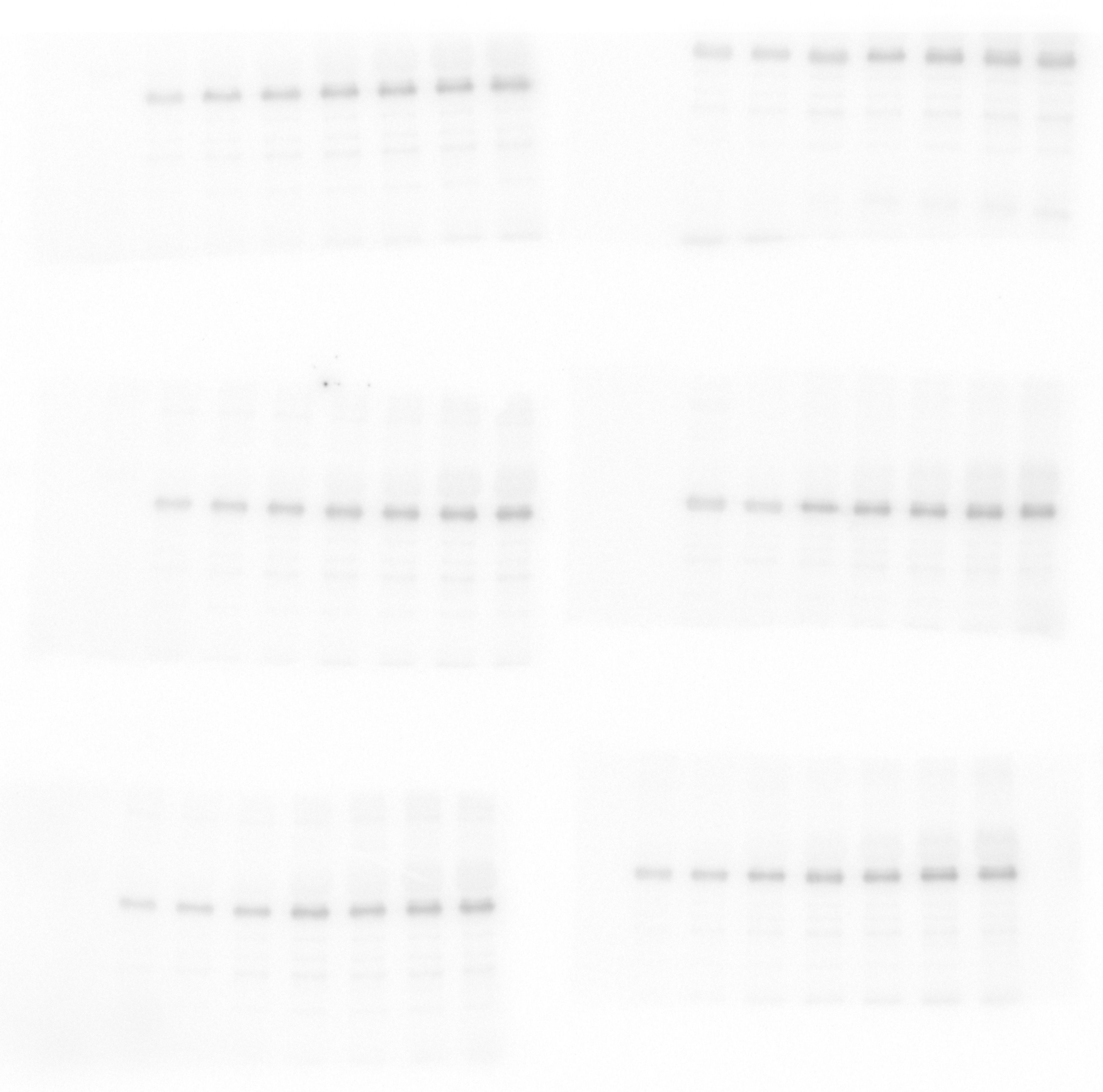

Supplement: Figure 2—figure supplement 2—source data 1. [file elife-86090-fig2-figsupp2-data1.zip › Figure 2-figure supplement 2-source data 1/Figure 2 - figure supplement 2 - radioactive gels.tif]

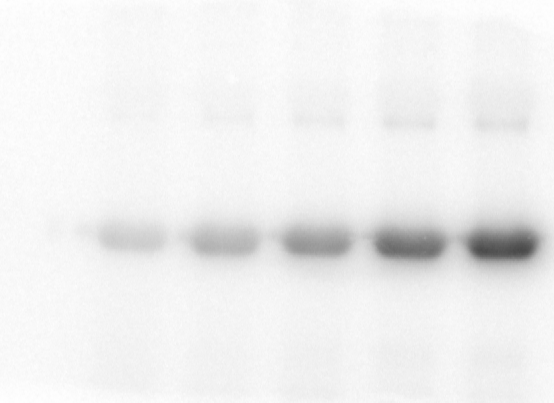

Supplement: Figure 2—figure supplement 3—source data 1. [file elife-86090-fig2-figsupp3-data1.zip › Figure 2-figure supplement 3-source data 1/Figure 2 - figure supplement 3 B rep1.tif]

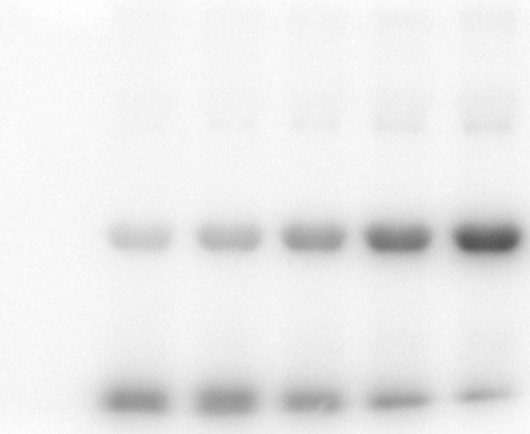

Supplement: Figure 2—figure supplement 3—source data 1. [file elife-86090-fig2-figsupp3-data1.zip › Figure 2-figure supplement 3-source data 1/Figure 2 - figure supplement 3 B rep3.tif]

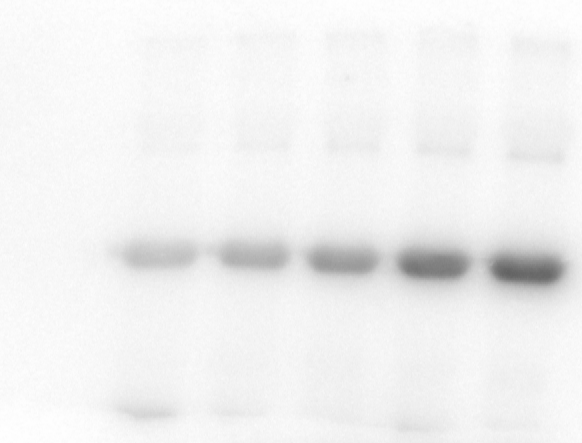

Supplement: Figure 2—figure supplement 3—source data 1. [file elife-86090-fig2-figsupp3-data1.zip › Figure 2-figure supplement 3-source data 1/Figure 2 - figure supplement 3 B rep2.tif]

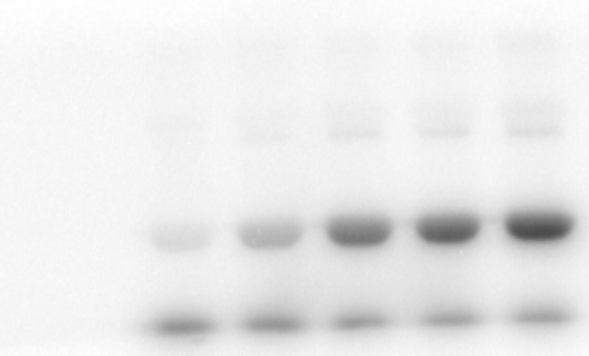

Supplement: Figure 2—figure supplement 3—source data 1. [file elife-86090-fig2-figsupp3-data1.zip › Figure 2-figure supplement 3-source data 1/Figure 2 - figure supplement 3 A rep2tif.tif]

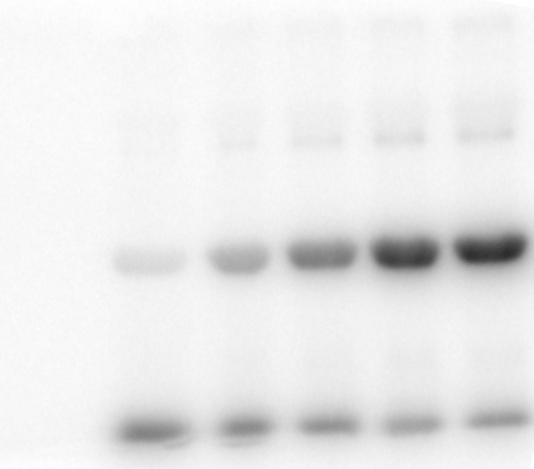

Supplement: Figure 2—figure supplement 3—source data 1. [file elife-86090-fig2-figsupp3-data1.zip › Figure 2-figure supplement 3-source data 1/Figure 2 - figure supplement 3 A rep3.tif]

Figure 2-figure supplement 3 gels

A

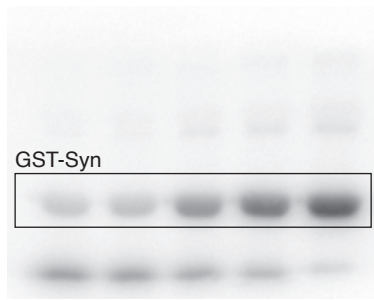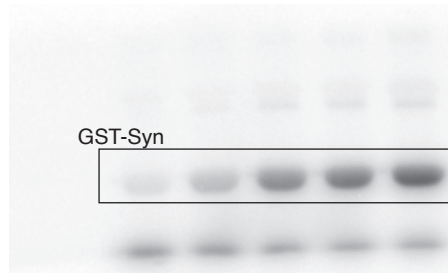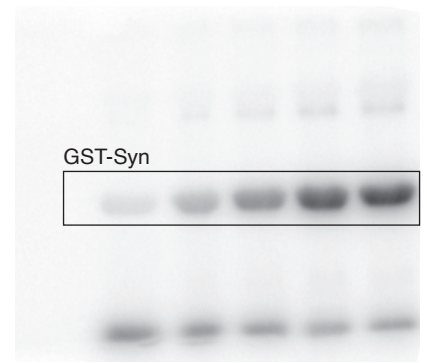

B

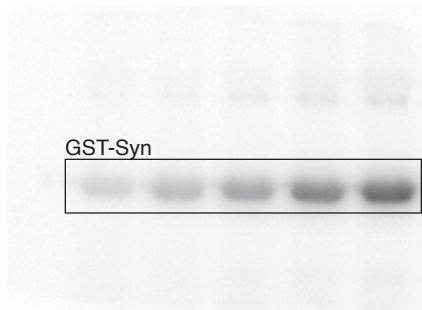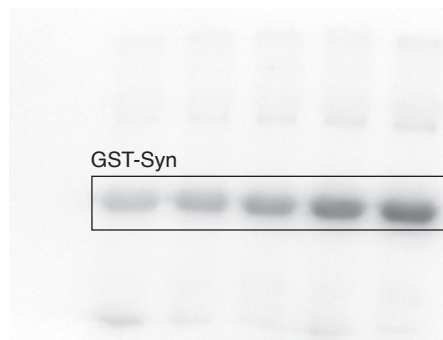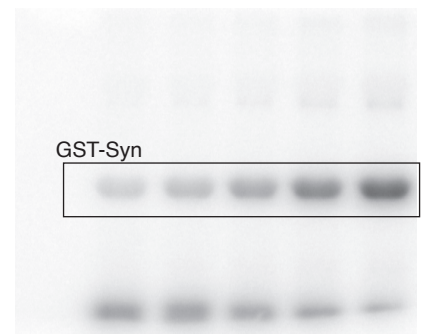

Supplement: Figure 2—figure supplement 3—source data 1. [file elife-86090-fig2-figsupp3-data1.zip › Figure 2-figure supplement 3-source data 1/Figure 2 - figure supplement 3-source data 1.pdf]

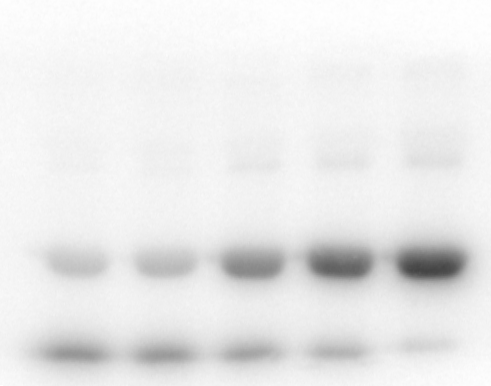

Supplement: Figure 2—figure supplement 3—source data 1. [file elife-86090-fig2-figsupp3-data1.zip › Figure 2-figure supplement 3-source data 1/Figure 2 - figure supplement 3 A rep1.tif]

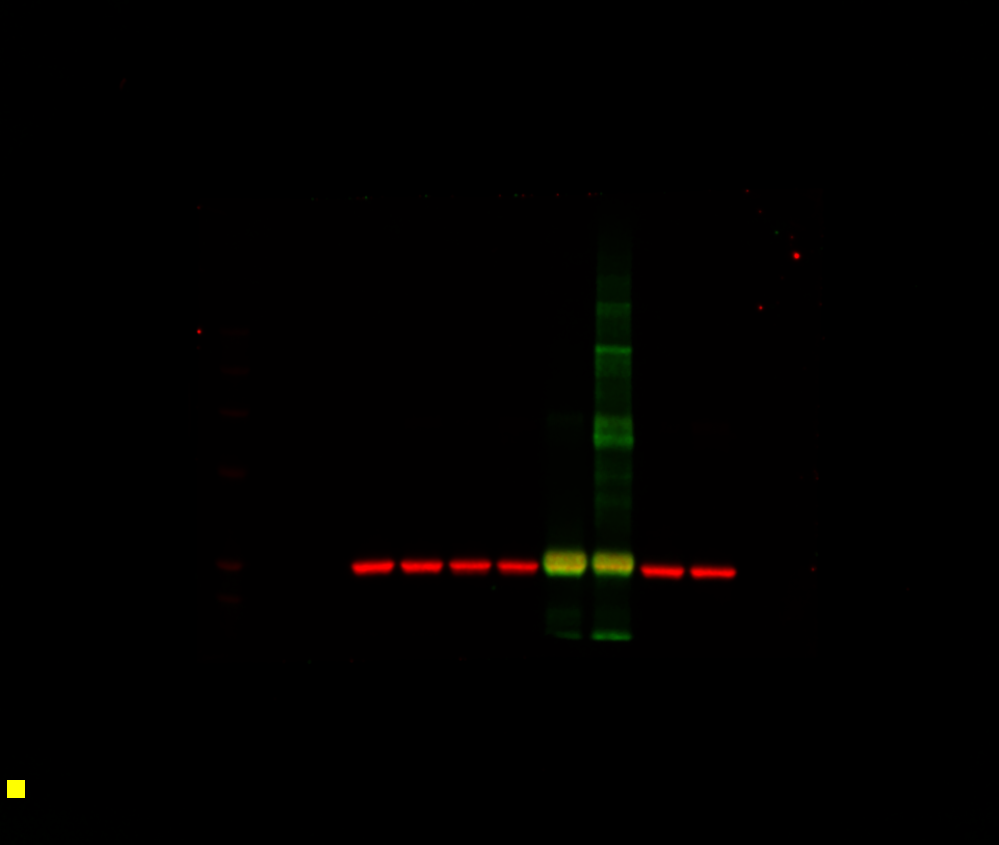

Supplement: Figure 3—source data 1. [file elife-86090-fig3-data1.zip › Figure 3-source data 1/Figure 3B merged signals.tif]

Figure 3B

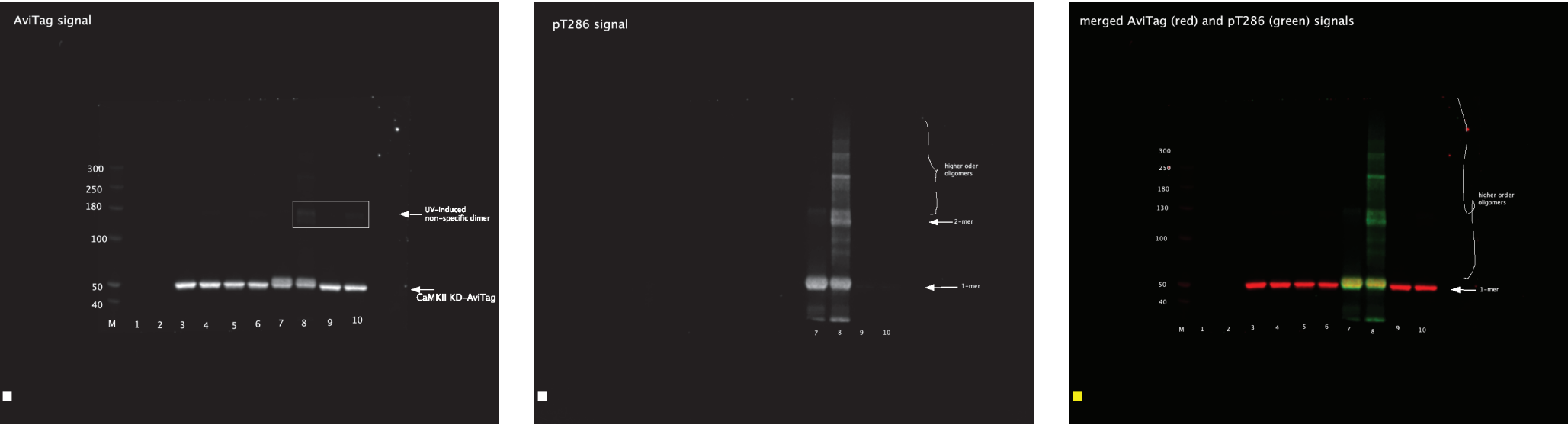

Supplement: Figure 3—source data 1. [file elife-86090-fig3-data1.zip › Figure 3-source data 1/Figure 3-source data 1.pdf]

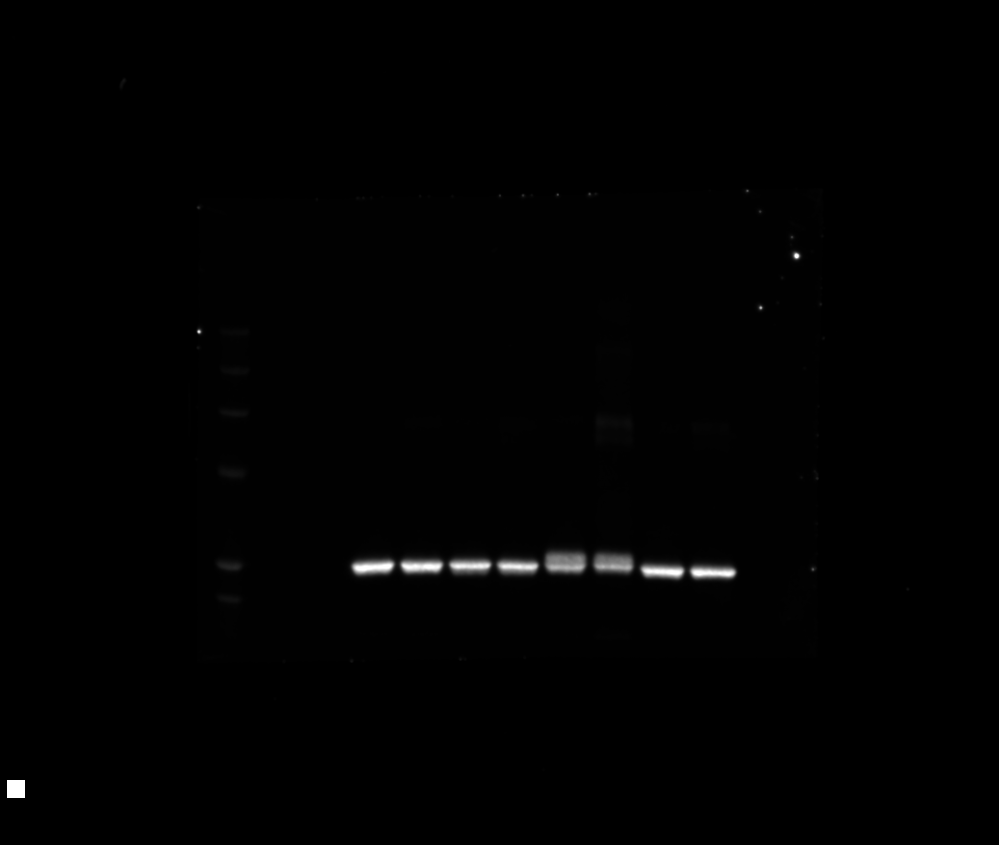

Supplement: Figure 3—source data 1. [file elife-86090-fig3-data1.zip › Figure 3-source data 1/Figure 3B AviTag signal.tif]

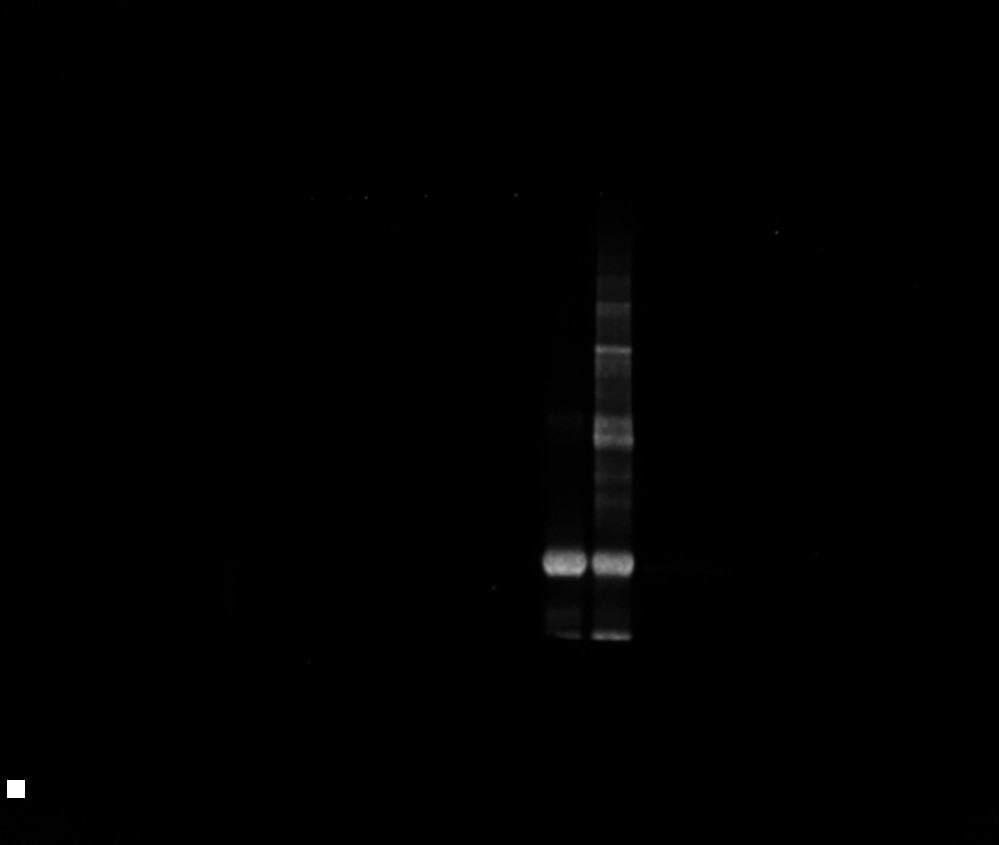

Supplement: Figure 3—source data 1. [file elife-86090-fig3-data1.zip › Figure 3-source data 1/Figure 3B pT286 signal.tif]

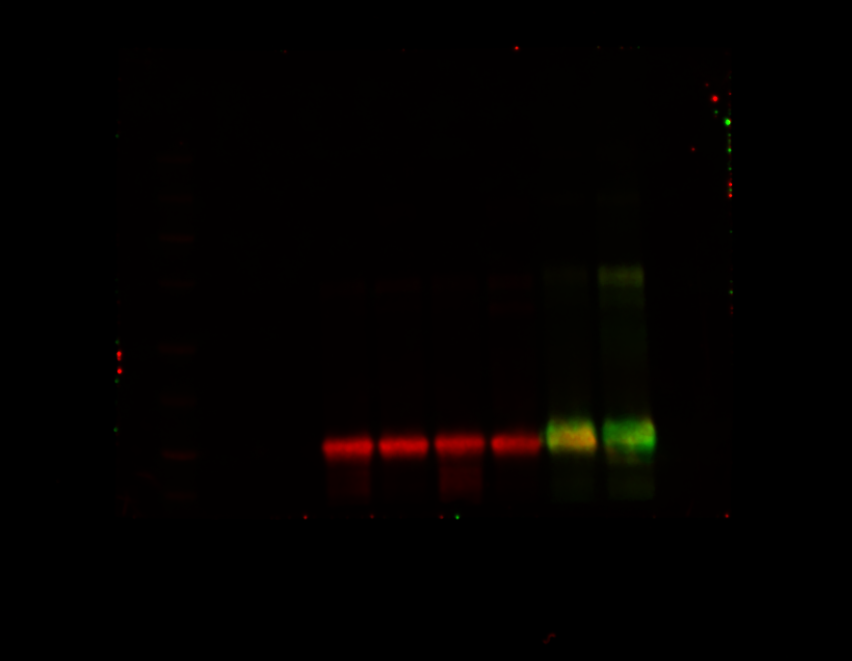

Supplement: Figure 3—figure supplement 1—source data 1. [file elife-86090-fig3-figsupp1-data1.zip › Figure 3-figure supplement 1-source data 1/Figure 3 - figure supplement 1 B merged signals_1.tif]

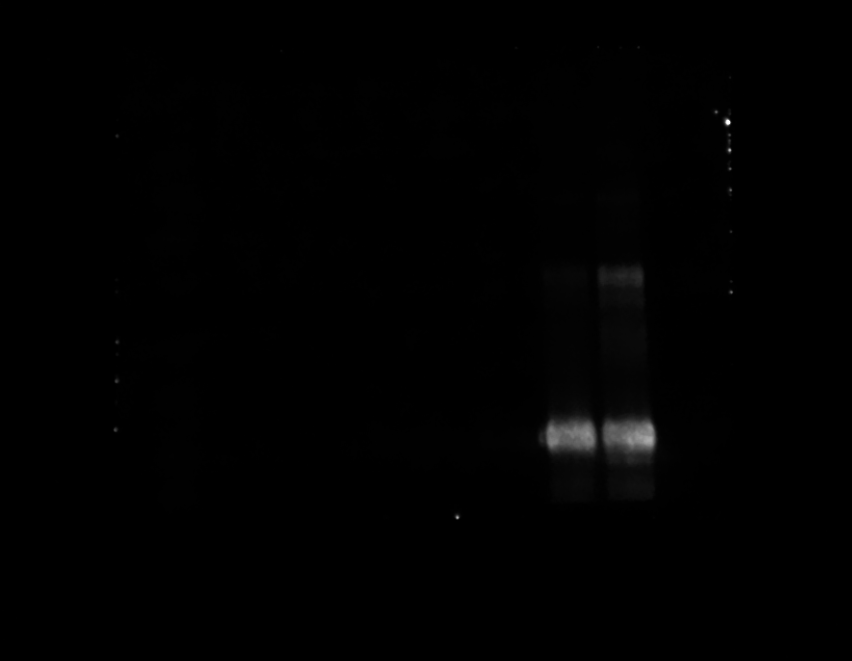

Supplement: Figure 3—figure supplement 1—source data 1. [file elife-86090-fig3-figsupp1-data1.zip › Figure 3-figure supplement 1-source data 1/Figure 3 - figure supplement 1 B pT286_1.tif]

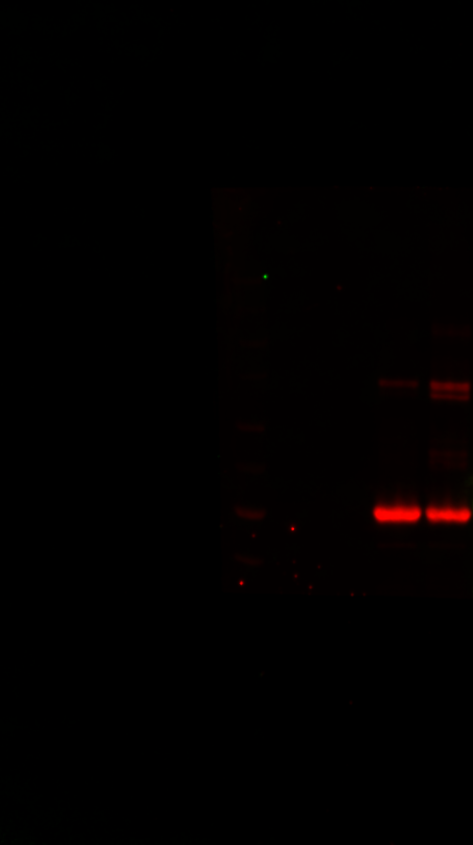

Supplement: Figure 3—figure supplement 1—source data 1. [file elife-86090-fig3-figsupp1-data1.zip › Figure 3-figure supplement 1-source data 1/Figure 3 - figure supplement 1 C blot.tif]

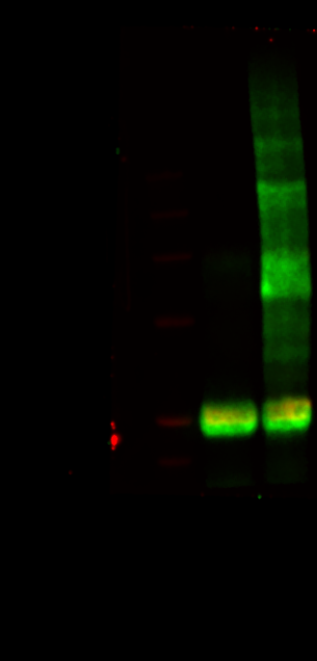

Supplement: Figure 3—figure supplement 1—source data 1. [file elife-86090-fig3-figsupp1-data1.zip › Figure 3-figure supplement 1-source data 1/Figure 3 - figure supplement 1 B merged signals_2.tif]

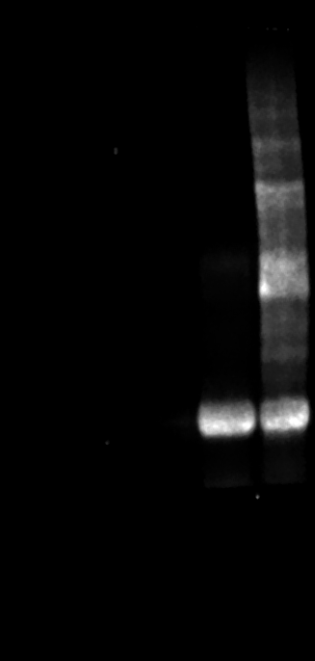

Supplement: Figure 3—figure supplement 1—source data 1. [file elife-86090-fig3-figsupp1-data1.zip › Figure 3-figure supplement 1-source data 1/Figure 3 - figure supplement 1 B pT286_2.tif]

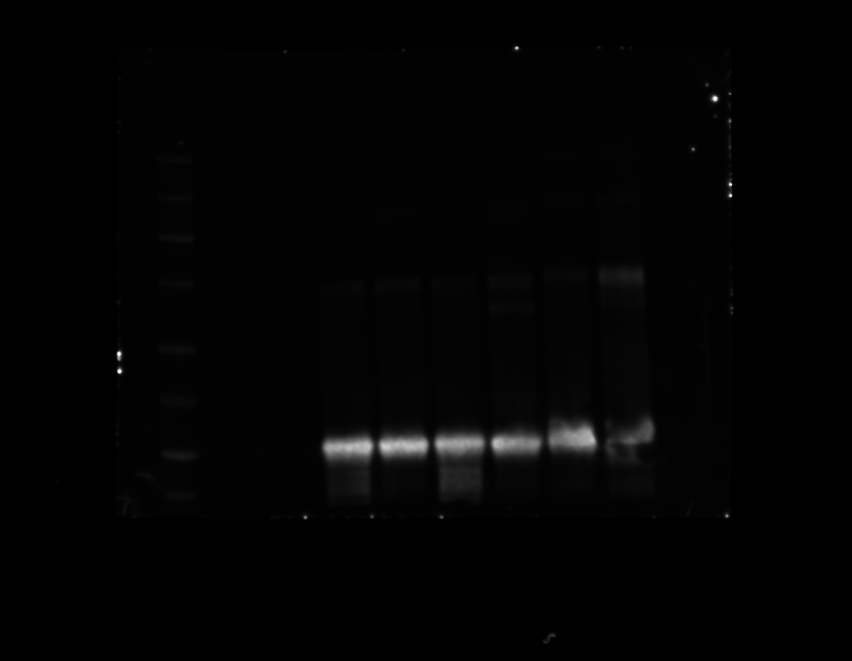

Supplement: Figure 3—figure supplement 1—source data 1. [file elife-86090-fig3-figsupp1-data1.zip › Figure 3-figure supplement 1-source data 1/Figure 3 - figure supplement 1 B AviTag_1.tif]

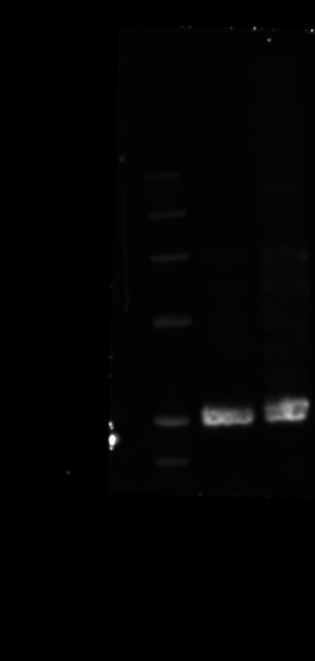

Supplement: Figure 3—figure supplement 1—source data 1. [file elife-86090-fig3-figsupp1-data1.zip › Figure 3-figure supplement 1-source data 1/Figure 3 - figure supplement 1 B AviTag_2.tif]

Figure 3-figure supplement 1 gels and blots

A

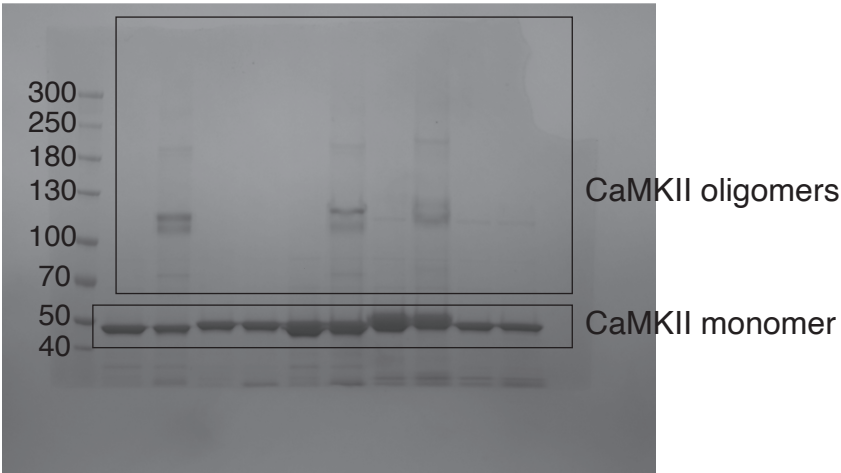

B AviTag signal

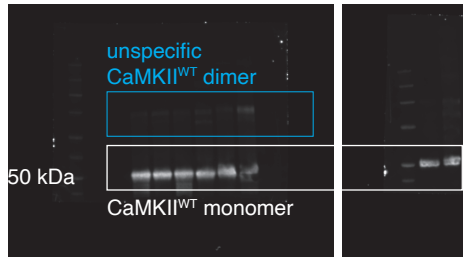

pT286 signal

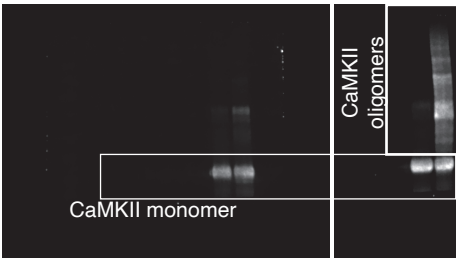

merged signals

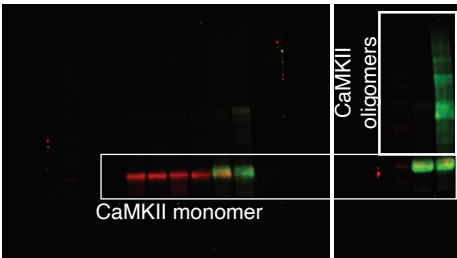

C

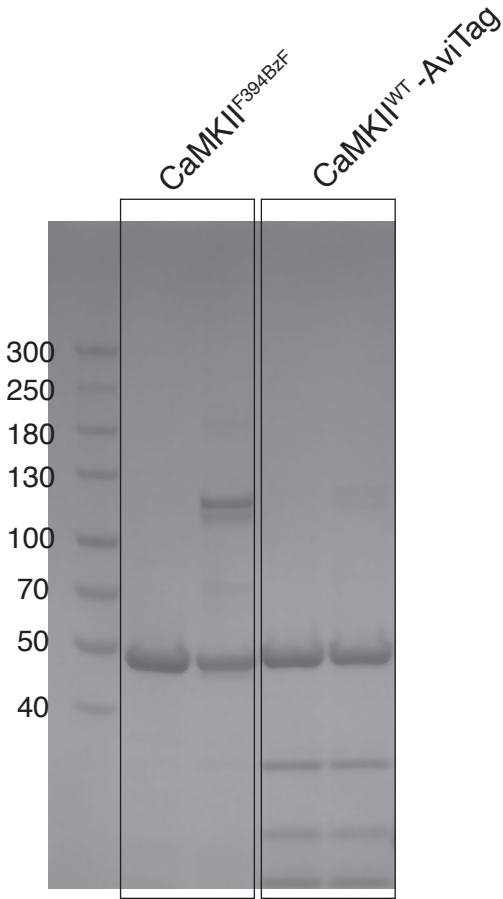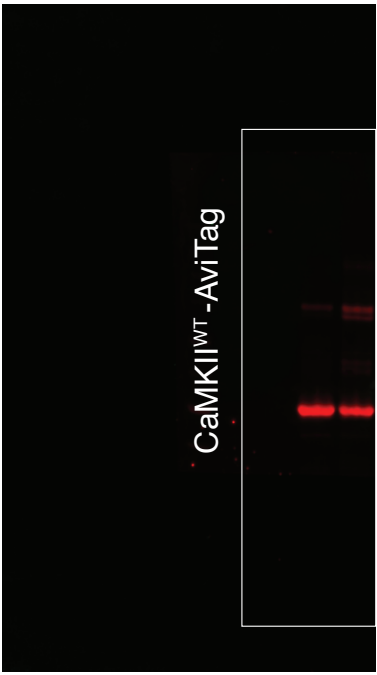

Supplement: Figure 3—figure supplement 1—source data 1. [file elife-86090-fig3-figsupp1-data1.zip › Figure 3-figure supplement 1-source data 1/Figure 3 - figure supplement 1-source data 1.pdf]

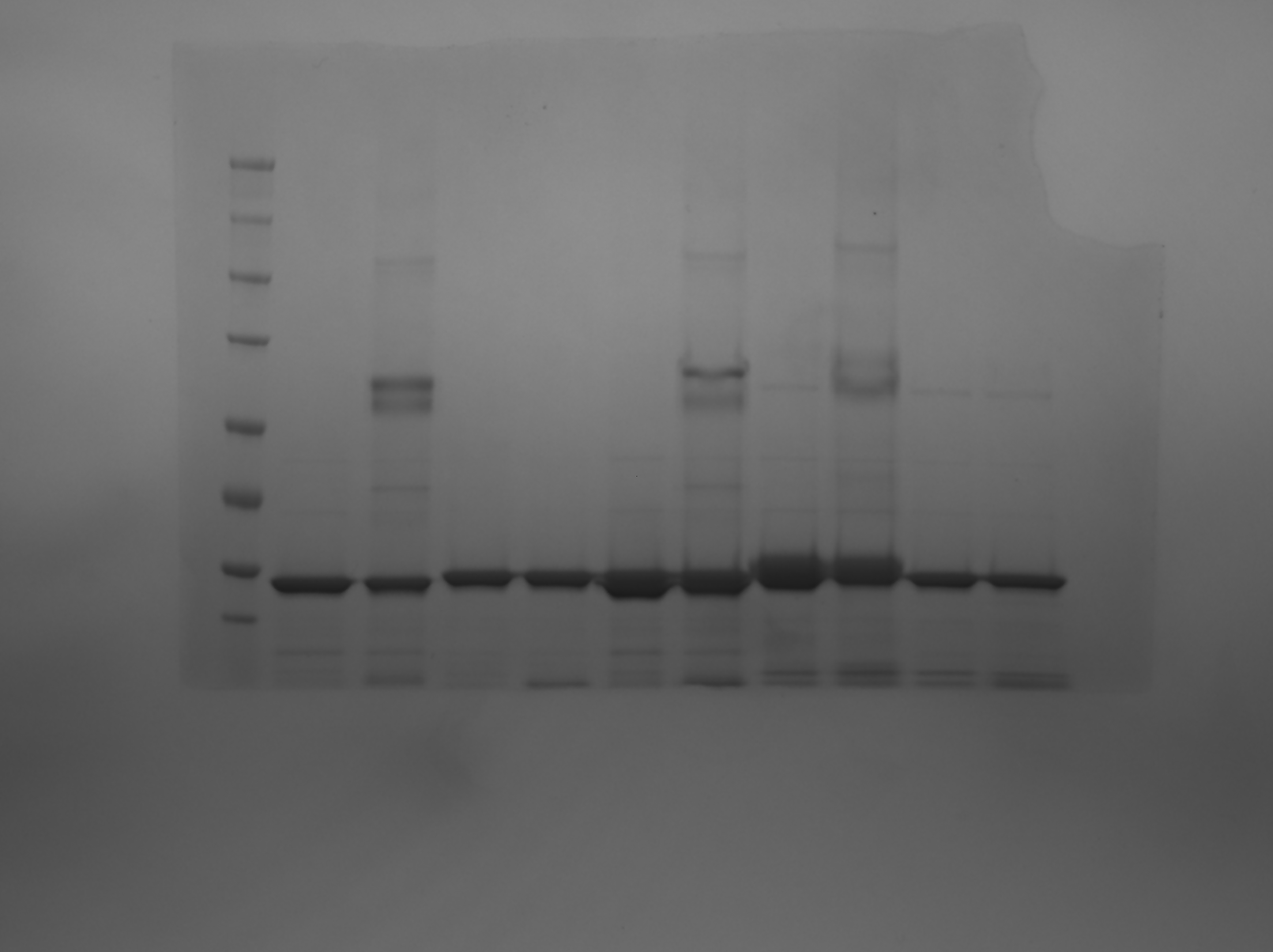

Supplement: Figure 3—figure supplement 1—source data 1. [file elife-86090-fig3-figsupp1-data1.zip › Figure 3-figure supplement 1-source data 1/Figure 3 - figure supplement 1 A.tif]

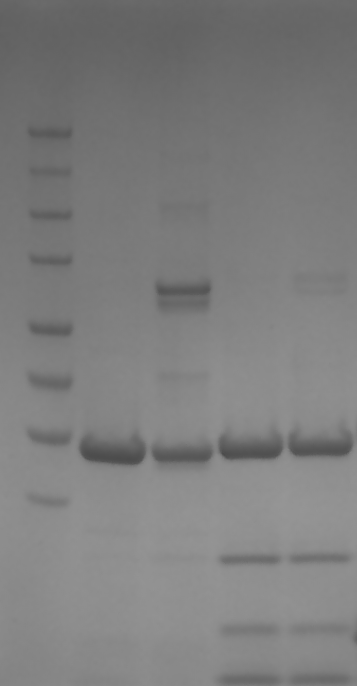

Supplement: Figure 3—figure supplement 1—source data 1. [file elife-86090-fig3-figsupp1-data1.zip › Figure 3-figure supplement 1-source data 1/Figure 3 - figure supplement 1 C_gel.tif]

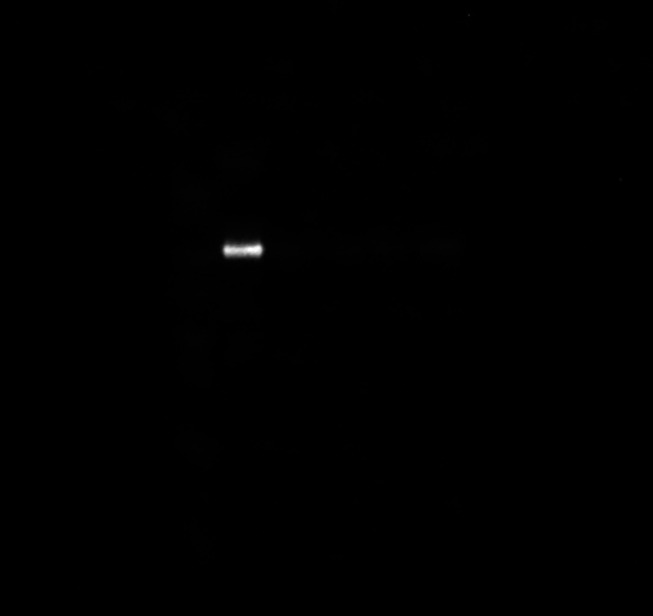

Supplement: Figure 7—figure supplement 2—source data 1. [file elife-86090-fig7-figsupp2-data1.zip › Figure 7-figure supplement 2-source data 1/Figure 7 - figure supplement 2 C pT286 signal.tif]

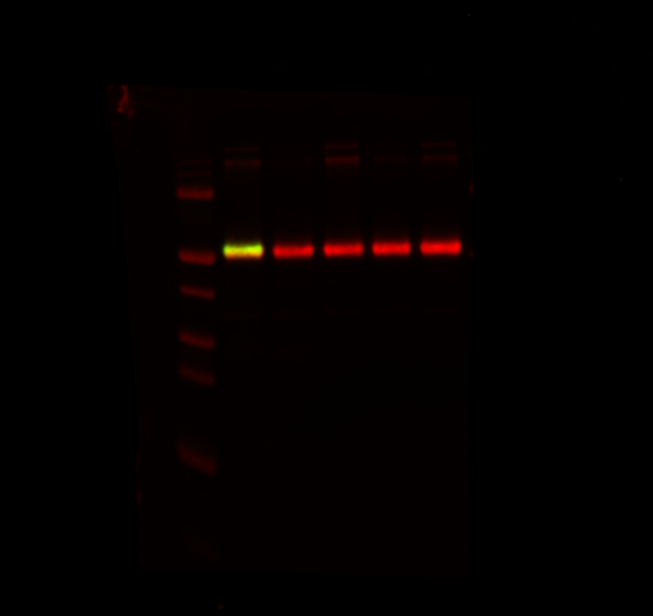

Supplement: Figure 7—figure supplement 2—source data 1. [file elife-86090-fig7-figsupp2-data1.zip › Figure 7-figure supplement 2-source data 1/Figure 7 - figure supplement 2 C merged signals.tif]

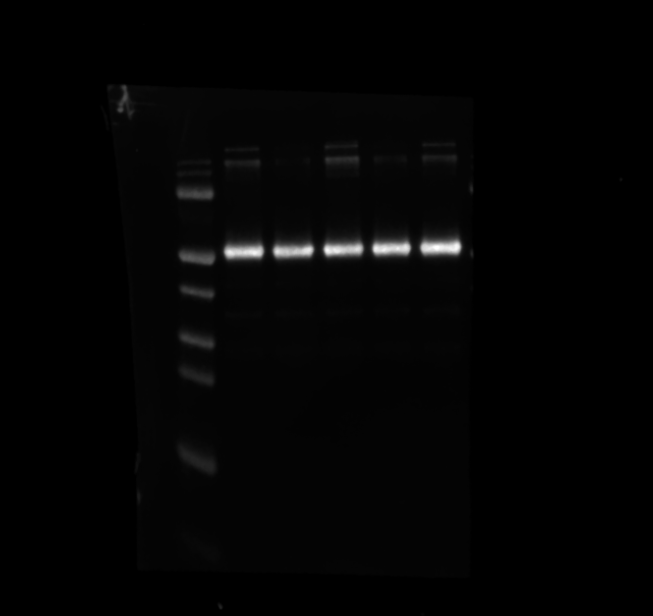

Supplement: Figure 7—figure supplement 2—source data 1. [file elife-86090-fig7-figsupp2-data1.zip › Figure 7-figure supplement 2-source data 1/Figure 7 - figure supplement 2 C pan CaMKII signal.tif]
