# Supplementary figures and images for: CaMKII autophosphorylation can occur between holoenzymes without subunit exchange (part 2 of 2)
Source: eLife. 2023 Aug 11;12:e86090. doi: 10.7554/eLife.86090 (PMC10468207; doi:10.7554/eLife.86090)

Figure 7-figure supplement 2 blots

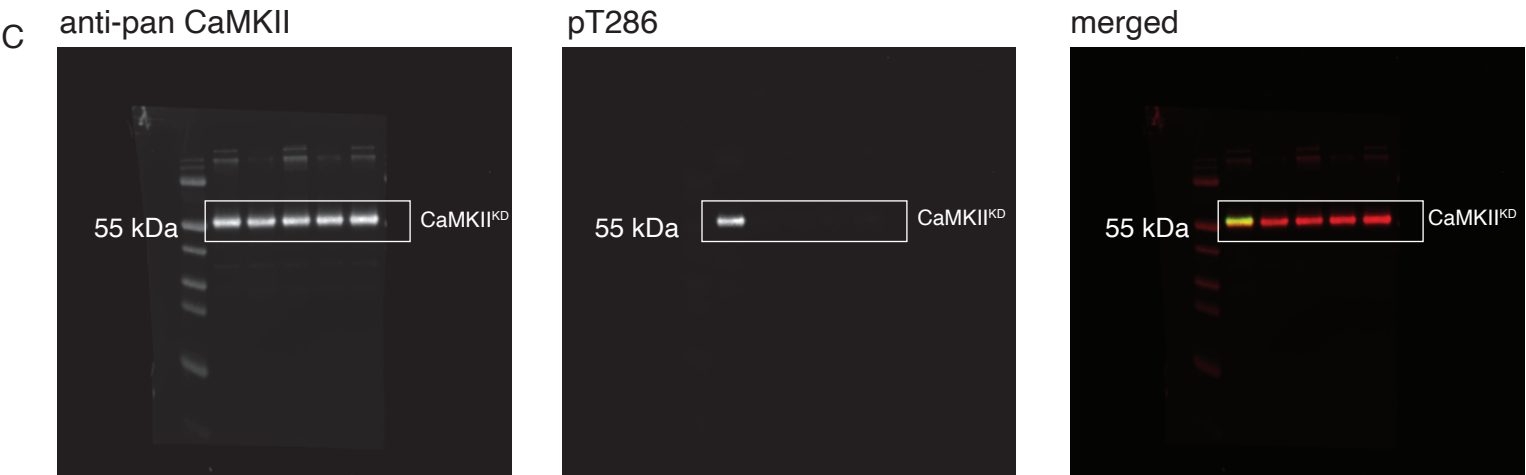

Supplement: Figure 7—figure supplement 2—source data 1. [file elife-86090-fig7-figsupp2-data1.zip › Figure 7-figure supplement 2-source data 1/Figure 7 - figure supplement 2-source data 1.pdf]
