# Supplementary material for: CaMKII autophosphorylation can occur between holoenzymes without subunit exchange
Source: eLife. 2023 Aug 11;12:e86090. doi: 10.7554/eLife.86090 (PMC10468207; doi:10.7554/eLife.86090)
Supplement: Supplementary file 1. — (A) Homotypic crosslinks (basal, 30 min). (B) Homotypic crosslinks (basal, 150 min). (C) Heterotypic crosslinks (basal, 30 min). (D) Heterotypic crosslinks (basal, 150 min). (E) Homotypic crosslinks (activated, 30 min). (F) Homotypic crosslinks (activated, 150 min). (G) Heterotypic crosslinks (activated, 30 min). (H) Heterotypic crosslinks (activated, 150 min) (I) pT286 Heterotypic peptides (30 min). (J) pT286 Heterotypic peptides (150 min). [file elife-86090-supp1.pdf]

Supplementary File 1A

Homotypic crosslinks (basal, 30 min)

#	Peptide	domain_a	domain_b	crosslinked lysines
1	AGAYDFPSPEWDTVTPEAKDLINK(19)-NFSGGKSGGNK(6)	kinase	linker	245-317
2	CVKVLAGQEYAAK(3)-LLKHPNIVR(3)	kinase	kinase	32-68
3	DGKWQIVHFHR(3)-DHQKLER(4)	hub	kinase	461-56
4	DGKWQIVHFHR(3)-KQEIIK(1)	hub	hub	461-347
5	DGKWQIVHFHR(3)-KSDGVK(1)	hub	linker	461-323
6	DHQKLER(4)-DHQKLER(4)	kinase	kinase	56-56
7	DHQKLER(4)-IINTKK(5)	kinase	kinase	56-47
8	DHQKLER(4)-KSDGVK(1)	kinase	linker	56-323
9	DHQKLER(4)-LKGAAVK(2)	kinase	kinase	56-148
10	DLINKMLTINPSK(5)-DHQKLER(4)	kinase	kinase	250-56
11	DLINKMLTINPSK(5)-DLKPENLLLASK(3)	kinase	kinase	250-137
12	DLINKMLTINPSK(5)-KQEIIK(1)	kinase	hub	250-347
13	DLINKMLTINPSK(5)-LKGAAVK(2)	kinase	kinase	250-148
14	DLINKMLTINPSK(5)-MLTINPSKR(8)	kinase	kinase	250-258
15	DLKPENLLLASK(3)-DHQKLER(4)	kinase	kinase	137-56
16	DLKPENLLLASK(3)-DLKPENLLLASK(3)	kinase	kinase	137-137
17	DLKPENLLLASK(3)-KSDGVK(1)	kinase	linker	137-323
18	DLKPENLLLASK(3)-LKGAAVK(2)	kinase	kinase	137-148
19	DLKPENLLLASK(3)-LLKHPNIVR(3)	kinase	kinase	137-68
20	DLKPENLLLASK(3)-MLTINPSKR(8)	kinase	kinase	137-258
21	DLKPENLLLASK(3)-NFSGGKSGGNK(6)	kinase	linker	137-317
22	DLKPENLLLASKLK(12)-DHQKLER(4)	kinase	kinase	146-56
23	DLKPENLLLASKLK(12)-IINTKK(5)	kinase	kinase	146-47
24	DLKPENLLLASKLK(12)-KSDGVK(1)	kinase	linker	146-323
25	DLKPENLLLASKLK(12)-LKGAAVK(2)	kinase	kinase	146-148
26	DLKPENLLLASKLK(12)-LLKHPNIVR(3)	kinase	kinase	146-68
27	DLKPENLLLASKLK(12)-MLTINPSKR(8)	kinase	kinase	146-258
28	DLKPENLLLASKLK(12)-NFSGGKSGGNK(6)	kinase	linker	146-317
29	ESSESTNTTIEDEDTKVR(16)-DGKWQIVHFHR(3)	linker	hub	344-461
30	ESSESTNTTIEDEDTKVR(16)-DHQKLER(4)	hub	kinase	344-56
31	ESSESTNTTIEDEDTKVR(16)-ESSESTNTTIEDEDTKVR(16)	hub	hub	344-344
32	ESSESTNTTIEDEDTKVR(16)-KQEIIK(1)	hub	hub	344-347
33	ESSESTNTTIEDEDTKVR(16)-KSDGVK(1)	hub	linker	344-323
34	ESSESTNTTIEDEDTKVR(16)-LKGAAVK(2)	hub	kinase	344-148
35	ESSESTNTTIEDEDTKVR(16)-LKGAILTTMLATR(2)	hub	regulatory	344-300

36	ESSESTNTTIEDEDTKVR(16)-LLKHPNIVR(3)	hub	kinase	344-68
37	ESSESTNTTIEDEDTKVR(16)-MLTINPSKR(8)	hub	kinase	344-258
38	ESSESTNTTIEDEDTKVR(16)-NFSGGKSGGNK(6)	hub	linker	344-317
39	ESSESTNTTIEDEDTKVRK(16)-KQEIIK(1)	hub	hub	344-347
40	FTEEYQLFEELGKGAFSVVR(13)-DGKWQIVHFHR(3)	kinase	hub	21-461
41	FTEEYQLFEELGKGAFSVVR(13)-DHQKLER(4)	kinase	kinase	21-56
42	FTEEYQLFEELGKGAFSVVR(13)-DLKPENLLLASK(3)	kinase	kinase	21-137
43	FTEEYQLFEELGKGAFSVVR(13)-DLKPENLLLASKLK(12)	kinase	kinase	21-146
44	FTEEYQLFEELGKGAFSVVR(13)-IINTKK(5)	kinase	kinase	21-47
45	FTEEYQLFEELGKGAFSVVR(13)-KSDGVK(1)	kinase	linker	21-323
46	FTEEYQLFEELGKGAFSVVR(13)-LKGAILTTMLATR(2)	kinase	regulatory	21-300
47	FTEEYQLFEELGKGAFSVVR(13)-MLTINPSKR(8)	kinase	kinase	21-258
48	FTEEYQLFEELGKGAFSVVR(13)-NFSGGKSGGNK(6)	kinase	linker	21-317
49	FTEEYQLFEELGKGAFSVVR(13)-VLAGQEYAAKIINTK(10)	kinase	kinase	21-42
50	FTEEYQLFEELGKGAFSVVRR(13)-IINTKKLSAR(5)	kinase	kinase	21-47
51	GAAVKLADFGLAIEVEGEQQAWFGFAGTPGYLSPEVLR(5)-LLKHPNIVR(3)	kinase	kinase	153-68
52	ITAAEALKHPWISHR(8)-DHQKLER(4)	kinase	kinase	267-56
53	ITAAEALKHPWISHR(8)-DLINKMLTINPSK(5)	kinase	kinase	267-250
54	ITAAEALKHPWISHR(8)-DLKPENLLLASK(3)	kinase	kinase	267-137
55	ITAAEALKHPWISHR(8)-KQEIIK(1)	kinase	hub	267-347
56	ITAAEALKHPWISHR(8)-KSDGVK(1)	kinase	linker	267-323
57	KLKGAILTTMLATR(3)-KLKGAILTTMLATR(1)	regulatory	regulatory	300-298
58	KQEIIK(1)-IINTKK(5)	hub	kinase	347-47
59	KQEIIK(1)-KQEIIK(1)	hub	hub	347-347
60	KQEIIK(1)-KSDGVK(1)	hub	linker	347-323
61	KQEIIK(1)-LKGAAVK(2)	hub	kinase	347-148
62	KSDGVKESSESTNTTIEDEDTK(1)-MLTINPSKR(8)	linker	kinase	323-258
63	KSDGVKESSESTNTTIEDEDTK(1)-NFSGGKSGGNK(6)	linker	linker	323-317
64	KSDGVKESSESTNTTIEDEDTK(6)-MLTINPSKR(8)	linker	kinase	328-258
65	KSDGVKESSESTNTTIEDEDTK(6)-NFSGGKSGGNK(6)	linker	linker	328-317
66	LKGAILTTMLATR(2)-DHQKLER(4)	regulatory	kinase	300-56
67	LKGAILTTMLATR(2)-DLKPENLLLASK(3)	regulatory	kinase	300-137
68	LKGAILTTMLATR(2)-KQEIIK(1)	regulatory	hub	300-347
69	LKGAILTTMLATR(2)-KSDGVK(1)	regulatory	linker	300-323
70	LKGAILTTMLATR(2)-LKGAAVK(2)	regulatory	kinase	300-148
71	LKGAILTTMLATR(2)-LKGAILTTMLATR(2)	regulatory	regulatory	300-300
72	LKGAILTTMLATR(2)-MLTINPSKR(8)	regulatory	kinase	300-258

73	LKGAILTTMLATR(2)-NFSGGKSGGNK(6)	regulatory	linker	300-317
74	LKGAILTTMLATR(2)-QETVDCLKK(8)	regulatory	regulatory	300-291
75	LLKHPNIVR(3)-DHQKLER(4)	kinase	kinase	68-56
76	LLKHPNIVR(3)-LKGAAVK(2)	kinase	kinase	68-148
77	LYQQIKAGAYDFPSPEWDTVTPEAK(6)-DGKWQIVHFHR(3)	kinase	hub	226-461
78	LYQQIKAGAYDFPSPEWDTVTPEAK(6)-DHQKLER(4)	kinase	kinase	226-56
79	LYQQIKAGAYDFPSPEWDTVTPEAK(6)-DLKPENLLLASKLK(12)	kinase	kinase	226-146
80	LYQQIKAGAYDFPSPEWDTVTPEAK(6)-ITAAEALKHPWISHR(8)	kinase	kinase	226-267
81	LYQQIKAGAYDFPSPEWDTVTPEAK(6)-KQEIIK(1)	kinase	hub	226-347
82	LYQQIKAGAYDFPSPEWDTVTPEAK(6)-KSDGVK(1)	kinase	linker	226-323
83	LYQQIKAGAYDFPSPEWDTVTPEAK(6)-LKGAAVK(2)	kinase	kinase	226-148
84	LYQQIKAGAYDFPSPEWDTVTPEAK(6)-LKGAILTTMLATR(2)	kinase	regulatory	226-300
85	LYQQIKAGAYDFPSPEWDTVTPEAK(6)-LLKHPNIVR(3)	kinase	kinase	226-68
86	LYQQIKAGAYDFPSPEWDTVTPEAK(6)-MLTINPSKR(8)	kinase	kinase	226-258
87	LYQQIKAGAYDFPSPEWDTVTPEAK(6)-NFSGGKSGGNK(6)	kinase	linker	226-317
88	LYQQIKAGAYDFPSPEWDTVTPEAK(6)-SDGVKESSESTNTTIEDEDTK(5)	kinase	linker	226-328
89	LYQQIKAGAYDFPSPEWDTVTPEAK(6)-VLAGQEYAAKIINTK(10)	kinase	kinase	226-42
90	MLTINPSKR(8)-DHQKLER(4)	kinase	kinase	258-56
91	MLTINPSKR(8)-IINTKK(5)	kinase	kinase	258-47
92	MLTINPSKR(8)-KQEIIK(1)	kinase	hub	258-347
93	MLTINPSKR(8)-KSDGVK(1)	kinase	linker	258-323
94	MLTINPSKR(8)-LKGAAVK(2)	kinase	kinase	258-148
95	MLTINPSKR(8)-MLTINPSKR(8)	kinase	kinase	258-258
96	MLTINPSKR(8)-NFSGGKSGGNK(6)	kinase	linker	258-317
97	NFSGGKSGGNK(6)-KQEIIK(1)	linker	hub	317-347
98	NFSGGKSGGNK(6)-KSDGVK(1)	linker	linker	317-323
99	NFSGGKSGGNK(6)-LKGAAVK(2)	linker	kinase	317-148
100	NFSGGKSGGNK(6)-NFSGGKSGGNK(6)	linker	linker	317-317
101	NSKPVHTTILNPHIHLMGDESACIAYIR(3)-DHQKLER(4)	hub	kinase	408-56
102	QETVDCLKK(8)-DHQKLER(4)	regulatory	kinase	291-56
103	QETVDCLKK(8)-KQEIIK(1)	regulatory	hub	291-347
104	QETVDCLKK(8)-KSDGVK(1)	regulatory	linker	291-323
105	QETVDCLKK(8)-LLKHPNIVR(3)	regulatory	kinase	291-68
106	QETVDCLKK(8)-NFSGGKSGGNK(6)	regulatory	linker	291-317
107	RDGKWQIVHFHR(4)-SGGNKKSDGVK(5)	hub	linker	461-322
108	RITAAEALKHPWISHR(9)-DLINKMLTINPSKR(13)	kinase	kinase	267-258
109	RITAAEALKHPWISHR(9)-DLINKMLTINPSKR(5)	kinase	kinase	267-250

110	RKLKGAILTTMLATR(2)-VLAGQEYAAKIINTK(10)	regulatory	kinase	298-42
111	RKLKGAILTTMLATR(4)-KSDGVK(1)	regulatory	linker	300-323
112	SDGVKESSESTNTTIEDEDTK(5)-DGKWQIVHFHR(3)	linker	hub	328-461
113	SDGVKESSESTNTTIEDEDTK(5)-DHQKLER(4)	linker	kinase	328-56
114	SDGVKESSESTNTTIEDEDTK(5)-DLKPENLLLASKLK(12)	linker	kinase	328-146
115	SDGVKESSESTNTTIEDEDTK(5)-ESSESTNTTIEDEDTKVR(16)	linker	linker	328-344
116	SDGVKESSESTNTTIEDEDTK(5)-IINTKK(5)	linker	kinase	328-47
117	SDGVKESSESTNTTIEDEDTK(5)-ITAAEALKHPWISHR(8)	linker	kinase	328-267
118	SDGVKESSESTNTTIEDEDTK(5)-KQEIIK(1)	linker	hub	328-347
119	SDGVKESSESTNTTIEDEDTK(5)-LKGAILTTMLATR(2)	linker	regulatory	328-300
120	SDGVKESSESTNTTIEDEDTK(5)-LLKHPNIVR(3)	linker	kinase	328-68
121	SDGVKESSESTNTTIEDEDTK(5)-MLTINPSKR(8)	linker	kinase	328-258
122	SDGVKESSESTNTTIEDEDTK(5)-NFSGGKSGGNK(6)	linker	linker	328-317
123	SDGVKESSESTNTTIEDEDTK(5)-RKLKGAILTTMLATR(2)	linker	regulatory	328-298
124	SDGVKESSESTNTTIEDEDTK(5)-VLAGQEYAAKIINTK(10)	linker	kinase	328-42
125	VLAGQEYAAKIINTK(10)-DHQKLER(4)	kinase	kinase	42-56
126	VLAGQEYAAKIINTK(10)-DLKPENLLLASK(3)	kinase	kinase	42-137
127	VLAGQEYAAKIINTK(10)-DLKPENLLLASKLK(12)	kinase	kinase	42-146
128	VLAGQEYAAKIINTK(10)-IINTKK(5)	kinase	kinase	42-47
129	VLAGQEYAAKIINTK(10)-KSDGVK(1)	kinase	linker	42-323
130	VLAGQEYAAKIINTK(10)-LKGAAVK(2)	kinase	kinase	42-148
131	VLAGQEYAAKIINTK(10)-LKGAILTTMLATR(2)	kinase	regulatory	42-300
132	VLAGQEYAAKIINTK(10)-LLKHPNIVR(3)	kinase	kinase	42-68
133	VLAGQEYAAKIINTK(10)-MLTINPSKR(8)	kinase	kinase	42-258
134	VLAGQEYAAKIINTK(10)-NFSGGKSGGNK(6)	kinase	linker	42-317
135	VLAGQEYAAKIINTK(10)-VLAGQEYAAKIINTK(10)	kinase	kinase	42-42
136	VLAGQEYAAKIINTKK(10)-DHQKLER(4)	kinase	kinase	42-56

Homotypic crosslinks (basal, 150 min)

#	Peptide	domain_a	domain_b	crosslinked lysines
1	AGAYDFPSPEWDTVTPEAKDLINK(19)-DHQKLER(4)	kinase	kinase	245-56
2	AGAYDFPSPEWDTVTPEAKDLINK(19)-DLKPENLLLASK(3)	kinase	kinase	245-137
3	AGAYDFPSPEWDTVTPEAKDLINK(19)-IINTKK(5)	kinase	kinase	245-47
4	AGAYDFPSPEWDTVTPEAKDLINK(19)-ITAAEALKHPWISHR(8)	kinase	kinase	245-267
5	AGAYDFPSPEWDTVTPEAKDLINK(19)-KSDGVK(1)	kinase	linker	245-323
6	AGAYDFPSPEWDTVTPEAKDLINK(19)-LKGAAVK(2)	kinase	kinase	245-148
7	AGAYDFPSPEWDTVTPEAKDLINK(19)-LLKHPNIVR(3)	kinase	kinase	245-68
8	AGAYDFPSPEWDTVTPEAKDLINK(19)-MLTINPSKR(8)	kinase	kinase	245-258
9	AGAYDFPSPEWDTVTPEAKDLINK(19)-NFSGGKSGGNK(6)	kinase	linker	245-317
10	CVKVLAGQEYAAK(3)-DHQKLER(4)	kinase	kinase	32-56
11	DGKWQIVHFHR(3)-KQEIIK(1)	hub	hub	461-347
12	DGKWQIVHFHR(3)-KSDGVK(1)	hub	linker	461-323
13	DHQKLER(4)-DHQKLER(4)	kinase	kinase	56-56
14	DHQKLER(4)-IINTKK(5)	kinase	kinase	56-47
15	DHQKLER(4)-KQEIIK(1)	kinase	hub	56-347
16	DHQKLER(4)-KSDGVK(1)	kinase	linker	56-323
17	DHQKLER(4)-LKGAAVK(2)	kinase	kinase	56-148
18	DLINKMLTINPSK(5)-DHQKLER(4)	kinase	kinase	250-56
19	DLINKMLTINPSK(5)-IINTKK(5)	kinase	kinase	250-47
20	DLINKMLTINPSK(5)-KQEIIK(1)	kinase	hub	250-347
21	DLINKMLTINPSK(5)-KSDGVK(1)	kinase	linker	250-323
22	DLINKMLTINPSK(5)-LKGAAVK(2)	kinase	kinase	250-148
23	DLINKMLTINPSK(5)-MLTINPSKR(8)	kinase	kinase	250-258
24	DLINKMLTINPSK(5)-NFSGGKSGGNK(6)	kinase	linker	250-317
25	DLKPENLLLASK(3)-DHQKLER(4)	kinase	kinase	137-56
26	DLKPENLLLASK(3)-DLKPENLLLASK(3)	kinase	kinase	137-137
27	DLKPENLLLASK(3)-IINTKK(5)	kinase	kinase	137-47
28	DLKPENLLLASK(3)-KSDGVK(1)	kinase	linker	137-323
29	DLKPENLLLASK(3)-LKGAAVK(2)	kinase	kinase	137-148
30	DLKPENLLLASK(3)-MLTINPSKR(8)	kinase	kinase	137-258
31	DLKPENLLLASK(3)-NFSGGKSGGNK(6)	kinase	linker	137-317
32	DLKPENLLLASK(3)-QETVDCLKK(8)	kinase	regulatory	137-291
33	DLKPENLLLASKLK(12)-DHQKLER(4)	kinase	kinase	146-56
34	DLKPENLLLASKLK(12)-DLKPENLLLASKLK(12)	kinase	kinase	146-146
35	DLKPENLLLASKLK(12)-LKGAAVK(2)	kinase	kinase	146-148

36	DLKPENLLLASKLK(12)-MLTINPSKR(8)	kinase	kinase	146-258
37	DLKPENLLLASKLK(12)-NFSGGKSGGNK(6)	kinase	linker	146-317
38	ESSESTNTTIEDEDTKVR(16)-DHQKLER(4)	hub	kinase	344-56
39	ESSESTNTTIEDEDTKVR(16)-DLKPENLLLASKLK(12)	hub	kinase	344-146
40	ESSESTNTTIEDEDTKVR(16)-ESSESTNTTIEDEDTKVR(16)	hub	hub	344-344
41	ESSESTNTTIEDEDTKVR(16)-ITAAEALKHPWISHR(8)	hub	kinase	344-267
42	ESSESTNTTIEDEDTKVR(16)-KQEIIK(1)	hub	hub	344-347
43	ESSESTNTTIEDEDTKVR(16)-KSDGVK(1)	hub	linker	344-323
44	ESSESTNTTIEDEDTKVR(16)-LKGAAVK(2)	hub	kinase	344-148
45	ESSESTNTTIEDEDTKVR(16)-LKGAILTTMLATR(2)	hub	regulatory	344-300
46	ESSESTNTTIEDEDTKVR(16)-MLTINPSKR(8)	hub	kinase	344-258
47	ESSESTNTTIEDEDTKVR(16)-NFSGGKSGGNK(6)	hub	linker	344-317
48	ESSESTNTTIEDEDTKVR(16)-RKLKGAILTTMLATR(2)	hub	regulatory	344-298
49	ESSESTNTTIEDEDTKVRK(16)-KQEIIK(1)	hub	hub	344-347
50	ESSESTNTTIEDEDTKVRK(16)-KSDGVK(1)	hub	linker	344-323
51	FTEEYQLFEELGKGAFSVVR(13)-DGKWQIVHFHR(3)	kinase	hub	21-461
52	FTEEYQLFEELGKGAFSVVR(13)-DHQKLER(4)	kinase	kinase	21-56
53	FTEEYQLFEELGKGAFSVVR(13)-DLKPENLLLASK(3)	kinase	kinase	21-137
54	FTEEYQLFEELGKGAFSVVR(13)-DLKPENLLLASKLK(12)	kinase	kinase	21-146
55	FTEEYQLFEELGKGAFSVVR(13)-IINTKK(5)	kinase	kinase	21-47
56	FTEEYQLFEELGKGAFSVVR(13)-ITAAEALKHPWISHR(8)	kinase	kinase	21-267
57	FTEEYQLFEELGKGAFSVVR(13)-KQEIIK(1)	kinase	hub	21-347
58	FTEEYQLFEELGKGAFSVVR(13)-KSDGVK(1)	kinase	linker	21-323
59	FTEEYQLFEELGKGAFSVVR(13)-LKGAAVK(2)	kinase	kinase	21-148
60	FTEEYQLFEELGKGAFSVVR(13)-LKGAILTTMLATR(2)	kinase	regulatory	21-300
61	FTEEYQLFEELGKGAFSVVR(13)-MLTINPSKR(8)	kinase	kinase	21-258
62	FTEEYQLFEELGKGAFSVVR(13)-NFSGGKSGGNK(6)	kinase	linker	21-317
63	FTEEYQLFEELGKGAFSVVR(13)-VLAGQEYAAKIINTK(10)	kinase	kinase	21-42
64	FTEEYQLFEELGKGAFSVVRR(13)-IINTKKLSAR(5)	kinase	kinase	21-47
65	FTEEYQLFEELGKGAFSVVRR(13)-IINTKKLSAR(6)	kinase	kinase	21-48
66	GAAVKLADFGLAIEVEGEQQAWFGFAGTPGYLSPEVLR(5)-LLKHPNIVR(3)	kinase	kinase	153-68
67	IINTKK(5)-IINTKK(5)	kinase	kinase	47-47
68	IINTKK(5)-LKGAAVK(2)	kinase	kinase	47-148
69	ITAAEALKHPWISHR(8)-DLINKMLTINPSK(5)	kinase	kinase	267-250
70	ITAAEALKHPWISHR(8)-DLKPENLLLASK(3)	kinase	kinase	267-137
71	ITAAEALKHPWISHR(8)-KQEIIK(1)	kinase	hub	267-347
72	ITAAEALKHPWISHR(8)-KSDGVK(1)	kinase	linker	267-323

73	ITAAEALKHPWISHR(8)-MLTINPSKR(8)	kinase	kinase	267-258
74	KLKGAILTTMLATR(3)-KLKGAILTTMLATR(1)	regulatory	regulatory	300-298
75	KQEIIK(1)-IINTKK(5)	hub	kinase	347-47
76	KQEIIK(1)-KQEIIK(1)	hub	hub	347-347
77	KQEIIK(1)-KSDGVK(1)	hub	linker	347-323
78	KQEIIK(1)-LKGAAVK(2)	hub	kinase	347-148
79	KQEIIKVTEQLIEAISNGGFESYTK(1)-RDGKWQIVHFHR(4)	hub	hub	347-461
80	KQEIIKVTEQLIEAISNGGFESYTK(6)-DHQKLER(4)	hub	kinase	352-56
81	KQEIIKVTEQLIEAISNGGFESYTK(6)-NFSGGKSGGNK(6)	hub	linker	352-317
82	KSDGVKESSESTNTTIEDEDTK(6)-KQEIIK(1)	linker	hub	328-347
83	KSDGVKESSESTNTTIEDEDTK(6)-LKGAILTTMLATR(2)	linker	regulatory	328-300
84	KSDGVKESSESTNTTIEDEDTK(6)-NFSGGKSGGNK(6)	linker	linker	328-317
85	KSDGVKESSESTNTTIEDEDTKVR(22)-KQEIIK(1)	hub	hub	344-347
86	LKGAAVK(2)-LKGAAVK(2)	kinase	kinase	148-148
87	LKGAILTTMLATR(2)-DHQKLER(4)	regulatory	kinase	300-56
88	LKGAILTTMLATR(2)-DLKPENLLLASK(3)	regulatory	kinase	300-137
89	LKGAILTTMLATR(2)-KQEIIK(1)	regulatory	hub	300-347
90	LKGAILTTMLATR(2)-KSDGVK(1)	regulatory	linker	300-323
91	LKGAILTTMLATR(2)-MLTINPSKR(8)	regulatory	kinase	300-258
92	LKGAILTTMLATR(2)-NFSGGKSGGNK(6)	regulatory	linker	300-317
93	LKGAILTTMLATR(2)-QETVDCLKK(8)	regulatory	regulatory	300-291
94	LLKHPNIVR(3)-DHQKLER(4)	kinase	kinase	68-56
95	LLKHPNIVR(3)-KQEIIK(1)	kinase	hub	68-347
96	LLKHPNIVR(3)-LKGAAVK(2)	kinase	kinase	68-148
97	LLKHPNIVR(3)-MLTINPSKR(8)	kinase	kinase	68-258
98	LYQQIKAGAYDFPSPEWDTVTPEAK(6)-DGKWQIVHFHR(3)	kinase	hub	226-461
99	LYQQIKAGAYDFPSPEWDTVTPEAK(6)-DHQKLER(4)	kinase	kinase	226-56
100	LYQQIKAGAYDFPSPEWDTVTPEAK(6)-DLINKMLTINPSK(5)	kinase	kinase	226-250
101	LYQQIKAGAYDFPSPEWDTVTPEAK(6)-DLKPENLLLASK(3)	kinase	kinase	226-137
102	LYQQIKAGAYDFPSPEWDTVTPEAK(6)-DLKPENLLLASKLK(12)	kinase	kinase	226-146
103	LYQQIKAGAYDFPSPEWDTVTPEAK(6)-ESSESTNTTIEDEDTKVR(16)	kinase	hub	226-344
104	LYQQIKAGAYDFPSPEWDTVTPEAK(6)-IINTKK(5)	kinase	kinase	226-47
105	LYQQIKAGAYDFPSPEWDTVTPEAK(6)-ITAAEALKHPWISHR(8)	kinase	kinase	226-267
106	LYQQIKAGAYDFPSPEWDTVTPEAK(6)-KQEIIK(1)	kinase	hub	226-347
107	LYQQIKAGAYDFPSPEWDTVTPEAK(6)-KSDGVK(1)	kinase	linker	226-323
108	LYQQIKAGAYDFPSPEWDTVTPEAK(6)-LKGAAVK(2)	kinase	kinase	226-148
109	LYQQIKAGAYDFPSPEWDTVTPEAK(6)-LKGAILTTMLATR(2)	kinase	regulatory	226-300

110	LYQQIKAGAYDFPSPEWDTVTPEAK(6)-LLKHPNIVR(3)	kinase	kinase	226-68
111	LYQQIKAGAYDFPSPEWDTVTPEAK(6)-LYQQIKAGAYDFPSPEWDTVTPEAK(6)	kinase	kinase	226-226
112	LYQQIKAGAYDFPSPEWDTVTPEAK(6)-MLTINPSKR(8)	kinase	kinase	226-258
113	LYQQIKAGAYDFPSPEWDTVTPEAK(6)-NFSGGKSGGNK(6)	kinase	linker	226-317
114	LYQQIKAGAYDFPSPEWDTVTPEAK(6)-SDGVKESSESTNTTIEDEDTK(5)	kinase	linker	226-328
115	LYQQIKAGAYDFPSPEWDTVTPEAK(6)-VLAGQEYAAKIINTK(10)	kinase	kinase	226-42
116	MLTINPSKR(8)-DHQKLER(4)	kinase	kinase	258-56
117	MLTINPSKR(8)-IINTKK(5)	kinase	kinase	258-47
118	MLTINPSKR(8)-KQEIIK(1)	kinase	hub	258-347
119	MLTINPSKR(8)-KSDGVK(1)	kinase	linker	258-323
120	MLTINPSKR(8)-LKGAAVK(2)	kinase	kinase	258-148
121	MLTINPSKR(8)-MLTINPSKR(8)	kinase	kinase	258-258
122	MLTINPSKR(8)-NFSGGKSGGNK(6)	kinase	linker	258-317
123	NFSGGKSGGNK(6)-DHQKLER(4)	linker	kinase	317-56
124	NFSGGKSGGNK(6)-KQEIIK(1)	linker	hub	317-347
125	NFSGGKSGGNK(6)-KSDGVK(1)	linker	linker	317-323
126	NFSGGKSGGNK(6)-LKGAAVK(2)	linker	kinase	317-148
127	NFSGGKSGGNK(6)-NFSGGKSGGNK(6)	linker	linker	317-317
128	QEIIKVTEQLIEAISNGGFESYTK(5)-DGKWQIVHFHR(3)	hub	hub	352-461
129	QEIIKVTEQLIEAISNGGFESYTK(5)-DHQKLER(4)	hub	kinase	352-56
130	QEIIKVTEQLIEAISNGGFESYTK(5)-MLTINPSKR(8)	hub	kinase	352-258
131	QEIIKVTEQLIEAISNGGFESYTK(5)-NFSGGKSGGNK(6)	hub	linker	352-317
132	QETVDCLKK(8)-DHQKLER(4)	regulatory	kinase	291-56
133	QETVDCLKK(8)-KQEIIK(1)	regulatory	hub	291-347
134	QETVDCLKK(8)-KSDGVK(1)	regulatory	linker	291-323
135	QETVDCLKK(8)-LLKHPNIVR(3)	regulatory	kinase	291-68
136	QETVDCLKK(8)-NFSGGKSGGNK(6)	regulatory	linker	291-317
137	RDGKWQIVHFHR(4)-SGGNKKSDGVK(5)	hub	linker	461-322
138	RKLKGAILTTMLATR(2)-DLKPENLLLASK(3)	regulatory	kinase	298-137
139	RKLKGAILTTMLATR(2)-RKLKGAILTTMLATR(2)	regulatory	regulatory	298-298
140	RKLKGAILTTMLATR(2)-VLAGQEYAAKIINTK(10)	regulatory	kinase	298-42
141	RKLKGAILTTMLATR(4)-DHQKLER(4)	regulatory	kinase	300-56
142	SDGVKESSESTNTTIEDEDTK(5)-DGKWQIVHFHR(3)	linker	hub	328-461
143	SDGVKESSESTNTTIEDEDTK(5)-DHQKLER(4)	linker	kinase	328-56
144	SDGVKESSESTNTTIEDEDTK(5)-DLINKMLTINPSK(5)	linker	kinase	328-250
145	SDGVKESSESTNTTIEDEDTK(5)-DLKPENLLLASK(3)	linker	kinase	328-137
146	SDGVKESSESTNTTIEDEDTK(5)-DLKPENLLLASKLK(12)	linker	kinase	328-146

147	SDGVKESSESTNTTIEDEDTK(5)-ESSESTNTTIEDEDTKVR(16)	linker	hub	328-344
148	SDGVKESSESTNTTIEDEDTK(5)-ITAAEALKHPWISHR(8)	linker	kinase	328-267
149	SDGVKESSESTNTTIEDEDTK(5)-KQEIIK(1)	linker	hub	328-347
150	SDGVKESSESTNTTIEDEDTK(5)-KSDGVK(1)	linker	linker	328-323
151	SDGVKESSESTNTTIEDEDTK(5)-LKGAAVK(2)	linker	kinase	328-148
152	SDGVKESSESTNTTIEDEDTK(5)-LKGAILTTMLATR(2)	linker	regulatory	328-300
153	SDGVKESSESTNTTIEDEDTK(5)-LLKHPNIVR(3)	linker	kinase	328-68
154	SDGVKESSESTNTTIEDEDTK(5)-MLTINPSKR(8)	linker	kinase	328-258
155	SDGVKESSESTNTTIEDEDTK(5)-NFSGGKSGGNK(6)	linker	linker	328-317
156	SDGVKESSESTNTTIEDEDTK(5)-NFSGGKSGGNKK(6)	linker	linker	328-317
157	SDGVKESSESTNTTIEDEDTK(5)-RKLKGAILTTMLATR(2)	linker	regulatory	328-298
158	SDGVKESSESTNTTIEDEDTK(5)-VLAGQEYAAKIINTK(10)	linker	kinase	328-42
159	VLAGQEYAAKIINTK(10)-DHQKLER(4)	kinase	kinase	42-56
160	VLAGQEYAAKIINTK(10)-DLKPENLLLASK(3)	kinase	kinase	42-137
161	VLAGQEYAAKIINTK(10)-DLKPENLLLASKLK(12)	kinase	kinase	42-146
162	VLAGQEYAAKIINTK(10)-KSDGVK(1)	kinase	linker	42-323
163	VLAGQEYAAKIINTK(10)-LKGAAVK(2)	kinase	kinase	42-148
164	VLAGQEYAAKIINTK(10)-LKGAILTTMLATR(2)	kinase	regulatory	42-300
165	VLAGQEYAAKIINTK(10)-LLKHPNIVR(3)	kinase	kinase	42-68
166	VLAGQEYAAKIINTK(10)-MLTINPSKR(8)	kinase	kinase	42-258
167	VLAGQEYAAKIINTK(10)-NFSGGKSGGNK(6)	kinase	linker	42-317
168	VLAGQEYAAKIINTKK(10)-DHQKLER(4)	kinase	kinase	42-56

Heterotypic crosslinks (basal, 30 min)

#	Peptides	domain_a	domain_b	crosslinked lysines	R sample 1	R sample 2
1	DGKWQIVHFHR(3)-DLKPENLLLASK(3)	hub	kinase	461-137	0,15	0,12
2	LLKHPNIVR(3)-KQEIIK(1)	kinase	hub	68-347	0,08	0,13
3	ESSESTNTTIEDEDTKVR(16)-IINTKK(5)	hub	kinase	344-47	0,02	0,05

Supplementary File 1D

Heterotypic crosslinks (basal, 150 min)

#	Peptides	domain_a	domain_b	crosslinked lysines	R sample 1	R sample 2
1	DGKWQIVHFHR(3)-DLKPENLLLASK(3)	hub	kinase	461-137	0,12	0,09
2	DLKPENLLLASK(3)-KQEIIK(1)	kinase	hub	137-347	0,09	0,08
3	DLKPENLLLASKLK(12)-DLINKMLTINPSK(5)	kinase	kinase	146-250	0,05	0,08
4	DLKPENLLLASKLK(12)-DLKPENLLLASK(3)	kinase	kinase	146-137	0,10	0,08
5	DLKPENLLLASKLK(12)-IINTKK(5)	kinase	kinase	146-47	0,06	0,07
6	ESSESTNTTIEDEDTKVR(16)-DLKPENLLLASK(3)	hub	kinase	344-137	0,09	0,13
7	ITAAEALKHPWISHR(8)-DLKPENLLLASKLK(12)	kinase	kinase	267-146	0,07	0,28
8	VLAGQEYAAKIINTK(10)-DLINKMLTINPSK(5)	kinase	kinase	42-250	0,06	0,06

Homotypic crosslinks (activated, 30 min)				
#	Peptide	domain_a	domain_b	crosslinked lysines
1	AGAYDFPSPEWDTVTPEAKDLINK(19)-DHQKLER(4)	kinase	kinase	245-56
2	AGAYDFPSPEWDTVTPEAKDLINK(19)-DLKPENLLLASK(3)	kinase	kinase	245-137
3	AGAYDFPSPEWDTVTPEAKDLINK(19)-ITAAEALKHPWISHR(8)	kinase	kinase	245-267
4	AGAYDFPSPEWDTVTPEAKDLINK(19)-KQEIIK(1)	kinase	hub	245-347
5	CVKVLAGQEYAAK(3)-IINTKK(5)	kinase	kinase	32-47
6	CVKVLAGQEYAAK(3)-LLKHPNIVR(3)	kinase	kinase	32-68
7	DGKWQIVHFHR(3)-KQEIIK(1)	hub	hub	461-347
8	DGKWQIVHFHR(3)-KSDGVK(1)	hub	linker	461-323
9	DGKWQIVHFHR(3)-NFSGGKSGGNK(6)	hub	linker	461-317
10	DHQKLER(4)-DHQKLER(4)	kinase	kinase	56-56
11	DHQKLER(4)-IINTKK(5)	kinase	kinase	56-47
12	DLINKMLTINPSK(5)-DLKPENLLLASK(3)	kinase	kinase	250-137
13	DLKPENLLLASK(3)-DHQKLER(4)	kinase	kinase	137-56
14	DLKPENLLLASK(3)-DLKPENLLLASK(3)	kinase	kinase	137-137
15	DLKPENLLLASK(3)-KSDGVK(1)	kinase	linker	137-323
16	DLKPENLLLASK(3)-LKGAAVK(2)	kinase	kinase	137-148
17	DLKPENLLLASK(3)-MLTINPSKR(8)	kinase	kinase	137-258
18	DLKPENLLLASK(3)-NFSGGKSGGNK(6)	kinase	linker	137-317
19	DLKPENLLLASKLK(12)-DHQKLER(4)	kinase	kinase	146-56
20	DLKPENLLLASKLK(12)-KSDGVK(1)	kinase	linker	146-323
21	DLKPENLLLASKLK(12)-LLKHPNIVR(3)	kinase	kinase	146-68
22	ESSESTNTTIEDEDTKVR(16)-DGKWQIVHFHR(3)	hub	hub	344-461
23	ESSESTNTTIEDEDTKVR(16)-ESSESTNTTIEDEDTKVR(16)	hub	hub	344-344
24	ESSESTNTTIEDEDTKVR(16)-KQEIIK(1)	hub	hub	344-347
25	ESSESTNTTIEDEDTKVR(16)-KSDGVK(1)	hub	linker	344-323
26	ESSESTNTTIEDEDTKVR(16)-NFSGGKSGGNK(6)	hub	linker	344-317
27	ESSESTNTTIEDEDTKVRK(16)-ESSESTNTTIEDEDTKVR(16)	hub	hub	344-344
28	ESSESTNTTIEDEDTKVRK(16)-KQEIIK(1)	hub	hub	344-347
29	IINTKK(5)-IINTKK(5)	kinase	kinase	47-47
30	IINTKK(5)-LKGAAVK(2)	kinase	kinase	47-148
31	ITAAEALKHPWISHR(8)-DLINKMLTINPSK(5)	kinase	kinase	267-250
32	ITAAEALKHPWISHR(8)-DLKPENLLLASKLK(12)	kinase	kinase	267-146
33	ITAAEALKHPWISHR(8)-ITAAEALKHPWISHR(8)	kinase	kinase	267-267
34	ITAAEALKHPWISHR(8)-KQEIIK(1)	kinase	hub	267-347
35	ITAAEALKHPWISHR(8)-KSDGVK(1)	kinase	linker	267-323

36	ITAAEALKHPWISHR(8)-LKGAAVK(2)	kinase	kinase	267-148
37	ITAAEALKHPWISHR(8)-MLTINPSKR(8)	kinase	kinase	267-258
38	ITAAEALKHPWISHR(8)-NFSGGKSGGNK(6)	kinase	linker	267-317
39	KQEIIK(1)-IINTKK(5)	hub	kinase	347-47
40	KQEIIK(1)-KSDGVK(1)	hub	linker	347-323
41	KQEIIK(1)-LKGAAVK(2)	hub	kinase	347-148
42	KSDGVKESSESTNTTIEDEDTK(6)-KQEIIK(1)	linker	hub	328-347
43	KSDGVKESSESTNTTIEDEDTK(6)-NFSGGKSGGNK(6)	linker	linker	328-317
44	LKGAAVK(2)-LKGAAVK(2)	kinase	kinase	148-148
45	LKGAILTTMLATR(2)-DHQKLER(4)	regulatory	kinase	300-56
46	LKGAILTTMLATR(2)-KSDGVK(1)	regulatory	linker	300-323
47	LKGAILTTMLATR(2)-NFSGGKSGGNK(6)	regulatory	linker	300-317
48	LLKHPNIVR(3)-DHQKLER(4)	kinase	kinase	68-56
49	LLKHPNIVR(3)-KQEIIK(1)	kinase	hub	68-347
50	LLKHPNIVR(3)-LLKHPNIVR(3)	kinase	kinase	68-68
51	LYQQIKAGAYDFPSPEWDTVTPEAK(6)-DHQKLER(4)	kinase	kinase	226-56
52	LYQQIKAGAYDFPSPEWDTVTPEAK(6)-DLKPENLLLASKLK(12)	kinase	kinase	226-146
53	LYQQIKAGAYDFPSPEWDTVTPEAK(6)-IINTKK(5)	kinase	kinase	226-47
54	LYQQIKAGAYDFPSPEWDTVTPEAK(6)-KSDGVK(1)	kinase	linker	226-323
55	LYQQIKAGAYDFPSPEWDTVTPEAK(6)-LYQQIKAGAYDFPSPEWDTVTPEAK(6)	kinase	kinase	226-226
56	LYQQIKAGAYDFPSPEWDTVTPEAK(6)-MLTINPSKR(8)	kinase	kinase	226-258
57	LYQQIKAGAYDFPSPEWDTVTPEAK(6)-NFSGGKSGGNK(6)	kinase	linker	226-317
58	MLTINPSKR(8)-DHQKLER(4)	kinase	kinase	258-56
59	MLTINPSKR(8)-IINTKK(5)	kinase	kinase	258-47
60	MLTINPSKR(8)-KQEIIK(1)	kinase	hub	258-347
61	MLTINPSKR(8)-KSDGVK(1)	kinase	linker	258-323
62	MLTINPSKR(8)-LKGAAVK(2)	kinase	kinase	258-148
63	MLTINPSKR(8)-MLTINPSKR(8)	kinase	kinase	258-258
64	MLTINPSKR(8)-NFSGGKSGGNK(6)	kinase	linker	258-317
65	NFSGGKSGGNK(6)-KQEIIK(1)	linker	hub	317-347
66	NFSGGKSGGNK(6)-KSDGVK(1)	linker	linker	317-323
67	NFSGGKSGGNK(6)-NFSGGKSGGNK(6)	linker	linker	317-317
68	QETVDCLKK(8)-LLKHPNIVR(3)	regulatory	kinase	291-68
69	RITAAEALKHPWISHR(9)-DLINKMLTINPSKR(13)	kinase	kinase	267-258
70	RITAAEALKHPWISHR(9)-DLINKMLTINPSKR(5)	kinase	kinase	267-250
71	SDGVKESSESTNTTIEDEDTK(5)-DGKWQIVHFHR(3)	linker	hub	328-461
72	SDGVKESSESTNTTIEDEDTK(5)-ESSESTNTTIEDEDTKVR(16)	linker	HUB	328-344

73	SDGVKESSESTNTTIEDEDTK(5)-KQEIIK(1)	linker	hub	328-347
74	SDGVKESSESTNTTIEDEDTK(5)-KSDGVK(1)	linker	linker	328-323
75	SDGVKESSESTNTTIEDEDTK(5)-LKGAAVK(2)	linker	kinase	328-148
76	SDGVKESSESTNTTIEDEDTK(5)-LLKHPNIVR(3)	linker	kinase	328-68
77	SDGVKESSESTNTTIEDEDTK(5)-MLTINPSKR(8)	linker	kinase	328-258
78	SDGVKESSESTNTTIEDEDTK(5)-NFSGGKSGGNK(6)	linker	linker	328-317
79	SDGVKESSESTNTTIEDEDTK(5)-NFSGGKSGGNKK(6)	linker	linker	328-317
80	SDGVKESSESTNTTIEDEDTK(5)-SDGVKESSESTNTTIEDEDTK(5)	linker	linker	328-328
81	SDGVKESSESTNTTIEDEDTKVR(21)-KQEIIK(1)	hub	hub	344-347
82	VLAGQEYAAKIINTK(10)-DHQKLER(4)	kinase	kinase	42-56
83	VLAGQEYAAKIINTK(10)-LLKHPNIVR(3)	kinase	kinase	42-68
84	VLAGQEYAAKIINTK(10)-MLTINPSKR(8)	kinase	kinase	42-258
85	VLAGQEYAAKIINTK(10)-VLAGQEYAAKIINTK(10)	kinase	kinase	42-42

Homotypic crosslinks (activated, 150 min)

#	Peptide	domain_a	domain_b	crosslinked lysines
1	AGAYDFPSPEWDTVTPEAKDLINK(19)-DGKWQIVHFHR(3)	kinase	hub	245-461
2	AGAYDFPSPEWDTVTPEAKDLINK(19)-ITAAEALKHPWISHR(8)	kinase	kinase	245-267
3	AGAYDFPSPEWDTVTPEAKDLINK(19)-MLTINPSKR(8)	kinase	kinase	245-258
4	AGAYDFPSPEWDTVTPEAKDLINK(19)-NFSGGKSGGNK(6)	kinase	linker	245-317
5	CVKVLAGQEYAAK(3)-IINTKK(5)	kinase	kinase	32-47
6	DGKWQIVHFHR(3)-KQEIIK(1)	hub	hub	461-347
7	DGKWQIVHFHR(3)-KSDGVK(1)	hub	linker	461-323
8	DGKWQIVHFHR(3)-NFSGGKSGGNK(6)	hub	linker	461-317
9	DHQKLER(4)-DHQKLER(4)	kinase	kinase	56-56
10	DHQKLER(4)-IINTKK(5)	kinase	kinase	56-47
11	DLINKMLTINPSK(5)-DHQKLER(4)	kinase	kinase	250-56
12	DLINKMLTINPSK(5)-DLINKMLTINPSK(5)	kinase	kinase	250-250
13	DLINKMLTINPSK(5)-NFSGGKSGGNK(6)	kinase	linker	250-317
14	DLINKMLTINPSKR(5)-DLINKMLTINPSKR(13)	kinase	kinase	250-258
15	DLKPENLLLASK(3)-DLKPENLLLASK(3)	kinase	kinase	137-137
16	DLKPENLLLASK(3)-LKGAAVK(2)	kinase	kinase	137-148
17	DLKPENLLLASK(3)-NFSGGKSGGNK(6)	kinase	linker	137-317
18	DLKPENLLLASKLK(12)-DHQKLER(4)	kinase	kinase	146-56
19	DLKPENLLLASKLK(12)-DLKPENLLLASKLK(12)	kinase	kinase	146-146
20	ESSESTNTTIEDEDTKVR(16)-DGKWQIVHFHR(3)	hub	hub	344-461
21	ESSESTNTTIEDEDTKVR(16)-ESSESTNTTIEDEDTKVR(16)	hub	hub	344-344
22	ESSESTNTTIEDEDTKVR(16)-ITAAEALKHPWISHR(8)	hub	kinase	344-267
23	ESSESTNTTIEDEDTKVR(16)-KQEIIK(1)	hub	hub	344-347
24	ESSESTNTTIEDEDTKVR(16)-KSDGVK(1)	hub	linker	344-323
25	ESSESTNTTIEDEDTKVR(16)-LLKHPNIVR(3)	hub	kinase	344-68
26	ESSESTNTTIEDEDTKVR(16)-MLTINPSKR(8)	hub	kinase	344-258
27	ESSESTNTTIEDEDTKVR(16)-NFSGGKSGGNK(6)	hub	linker	344-317
28	ESSESTNTTIEDEDTKVRK(16)-KQEIIK(1)	hub	hub	344-347
29	IINTKK(5)-IINTKK(5)	kinase	kinase	47-47
30	IINTKK(5)-LKGAAVK(2)	kinase	kinase	47-148
31	ITAAEALKHPWISHR(8)-DHQKLER(4)	kinase	kinase	267-56
32	ITAAEALKHPWISHR(8)-DLINKMLTINPSK(5)	kinase	kinase	267-250
33	ITAAEALKHPWISHR(8)-DLKPENLLLASK(3)	kinase	kinase	267-137
34	ITAAEALKHPWISHR(8)-KQEIIK(1)	kinase	hub	267-347
35	ITAAEALKHPWISHR(8)-KSDGVK(1)	kinase	linker	267-323

36	ITAAEALKHPWISHR(8)-MLTINPSKR(8)	kinase	kinase	267-258
37	ITAAEALKHPWISHR(8)-NFSGGKSGGNK(6)	kinase	linker	267-317
38	KQEIIK(1)-KQEIIK(1)	hub	hub	347-347
39	KQEIIK(1)-KSDGVK(1)	hub	linker	347-323
40	KQEIIK(1)-LKGAAVK(2)	hub	kinase	347-148
41	KQEIIKVTEQLIEAISNGGFESYTK(1)-RDGKWQIVHFHR(4)	hub	hub	347-461
42	KQEIIKVTEQLIEAISNGGFESYTK(6)-ESSESTNTTIEDEDTKVR(16)	hub	hub	352-344
43	KQEIIKVTEQLIEAISNGGFESYTK(6)-KSDGVK(1)	hub	linker	352-323
44	KSDGVKESSESTNTTIEDEDTK(6)-NFSGGKSGGNK(6)	linker	linker	328-317
45	LKGAILTTMLATR(2)-KQEIIK(1)	regulatory	hub	300-347
46	LKGAILTTMLATR(2)-KSDGVK(1)	regulatory	linker	300-323
47	LKGAILTTMLATR(2)-NFSGGKSGGNK(6)	regulatory	linker	300-317
48	LKGAILTTMLATR(2)-QETVDCLKK(8)	regulatory	regulatory	300-291
49	LLKHPNIVR(3)-DHQKLER(4)	kinase	kinase	68-56
50	LLKHPNIVR(3)-NFSGGKSGGNK(6)	kinase	linker	68-317
51	LYQQIKAGAYDFPSPEWDTVTPEAK(6)-DGKWQIVHFHR(3)	kinase	hub	226-461
52	LYQQIKAGAYDFPSPEWDTVTPEAK(6)-DHQKLER(4)	kinase	kinase	226-56
53	LYQQIKAGAYDFPSPEWDTVTPEAK(6)-DLINKMLTINPSK(5)	kinase	kinase	226-250
54	LYQQIKAGAYDFPSPEWDTVTPEAK(6)-ITAAEALKHPWISHR(8)	kinase	kinase	226-267
55	LYQQIKAGAYDFPSPEWDTVTPEAK(6)-LYQQIKAGAYDFPSPEWDTVTPEAK(6)	kinase	kinase	226-226
56	LYQQIKAGAYDFPSPEWDTVTPEAK(6)-MLTINPSKR(8)	kinase	kinase	226-258
57	LYQQIKAGAYDFPSPEWDTVTPEAK(6)-NFSGGKSGGNK(6)	kinase	linker	226-317
58	MLTINPSKR(8)-DHQKLER(4)	kinase	kinase	258-56
59	MLTINPSKR(8)-KQEIIK(1)	kinase	hub	258-347
60	MLTINPSKR(8)-KSDGVK(1)	kinase	linker	258-323
61	MLTINPSKR(8)-LKGAAVK(2)	kinase	kinase	258-148
62	MLTINPSKR(8)-MLTINPSKR(8)	kinase	kinase	258-258
63	MLTINPSKR(8)-NFSGGKSGGNK(6)	kinase	linker	258-317
64	NFSGGKSGGNK(6)-KQEIIK(1)	linker	hub	317-347
65	NFSGGKSGGNK(6)-KSDGVK(1)	linker	linker	317-323
66	NFSGGKSGGNK(6)-LKGAAVK(2)	linker	kinase	317-148
67	NFSGGKSGGNK(6)-NFSGGKSGGNK(6)	linker	linker	317-317
68	QEIIKVTEQLIEAISNGGFESYTK(5)-DGKWQIVHFHR(3)	hub	hub	352-461
69	RITAAEALKHPWISHR(9)-DLINKMLTINPSKR(13)	kinase	kinase	267-258
70	RITAAEALKHPWISHR(9)-DLINKMLTINPSKR(5)	kinase	kinase	267-250
71	SDGVKESSESTNTTIEDEDTK(5)-DGKWQIVHFHR(3)	linker	hub	328-461
72	SDGVKESSESTNTTIEDEDTK(5)-DHQKLER(4)	linker	kinase	328-56

73	SDGVKESSESTNTTIEDEDTK(5)-DLINKMLTINPSK(5)	linker	kinase	328-250
74	SDGVKESSESTNTTIEDEDTK(5)-ESSESTNTTIEDEDTKVR(16)	linker	hub	328-344
75	SDGVKESSESTNTTIEDEDTK(5)-ITAAEALKHPWISHR(8)	linker	kinase	328-267
76	SDGVKESSESTNTTIEDEDTK(5)-KQEIIK(1)	linker	hub	328-347
77	SDGVKESSESTNTTIEDEDTK(5)-KSDGVK(1)	linker	linker	328-323
78	SDGVKESSESTNTTIEDEDTK(5)-LKGAILTTMLATR(2)	linker	regulatory	328-300
79	SDGVKESSESTNTTIEDEDTK(5)-LLKHPNIVR(3)	linker	kinase	328-68
80	SDGVKESSESTNTTIEDEDTK(5)-MLTINPSKR(8)	linker	kinase	328-258
81	SDGVKESSESTNTTIEDEDTK(5)-NFSGGKSGGNK(6)	linker	linker	328-317
82	SDGVKESSESTNTTIEDEDTK(5)-NFSGGKSGGNKK(6)	linker	linker	328-317
83	SDGVKESSESTNTTIEDEDTK(5)-RKLKGAILTTMLATR(2)	linker	regulatory	328-298
84	SDGVKESSESTNTTIEDEDTK(5)-SDGVKESSESTNTTIEDEDTK(5)	linker	linker	328-328
85	VLAGQEYAAKIINTK(10)-DHQKLER(4)	kinase	kinase	42-56
86	VLAGQEYAAKIINTK(10)-VLAGQEYAAKIINTK(10)	kinase	kinase	42-42

Supplementary File 1G

Heterotypic crosslinks (activated, 30 min)

#	Peptides	domain_a	domain_b	crosslinked lysines	R sample 1	R sample 2
1	DLKPENLLLASK(3)-IINTKK(5)	kinase	kinase	137-47	1,63	0,22
2	FTEEYQLFEELGKGAFSVVR(13)-MLTINPSKR(8)	kinase	kinase	21-258	0,55	0,15
3	VLAGQEYAAKIINTK(10)-DLINKMLTINPSK(5)	kinase	kinase	42-250	0,35	0,34
4	DLKPENLLLASKLK(12)-DLKPENLLLASK(3)	kinase	kinase	146-137	0,34	0,22
5	ESSESTNTTIEDEDTKVR(16)-DLKPENLLLASK(3)	hub	kinase	344-137	0,33	0,19
6	ITAAEALKHPWISHR(8)-VLAGQEYAAKIINTK(10)	kinase	kinase	267-42	0,29	0,24
7	NFSGGKSGGNK(6)-DHQKLER(4)	linker	kinase	317-56	0,27	0,21
8	LKGAILTTMLATR(2)-DLKPENLLLASK(3)	regulatory	kinase	300-137	0,26	0,12
9	MLTINPSKR(8)-LLKHPNIVR(3)	kinase	kinase	258-68	0,25	0,19
10	LYQQIKAGAYDFPSPEWDTVTPEAK(6)-VLAGQEYAAKIINTK(10)	kinase	kinase	226-42	0,24	0,15
11	FTEEYQLFEELGKGAFSVVR(13)-LLKHPNIVR(3)	kinase	kinase	21-68	0,23	0,17
12	FTEEYQLFEELGKGAFSVVR(13)-LKGAAVK(2)	kinase	kinase	21-148	0,23	0,20
13	ITAAEALKHPWISHR(8)-IINTKK(5)	kinase	kinase	267-47	0,22	0,18
14	DLKPENLLLASKLK(12)-LKGAAVK(2)	kinase	kinase	146-148	0,22	0,27
15	LLKHPNIVR(3)-IINTKK(5)	kinase	kinase	68-47	0,21	0,22
16	AGAYDFPSPEWDTVTPEAKDLINK(19)-LLKHPNIVR(3)	kinase	kinase	245-68	0,19	0,35
17	DHQKLER(4)-KSDGVK(1)	kinase	linker	56-323	0,16	0,17
18	FTEEYQLFEELGKGAFSVVR(13)-DHQKLER(4)	kinase	kinase	21-56	0,15	0,12
19	VLAGQEYAAKIINTK(10)-DLKPENLLLASK(3)	kinase	kinase	42-137	0,15	0,21
20	ESSESTNTTIEDEDTKVR(16)-ITAAEALKHPWISHR(8)	hub	kinase	344-267	0,05	0,12

Supplementary File 1H

Heterotypic crosslinks (activated, 150 min)						
#	Peptides	domain_a	domain_b	crosslinked lysines	R sample 1	R sample 2
1	VLAGQEYAAKIINTK(10)-DLKPENLLLASKLK(12)	kinase	kinase	42-146	0,56	0,40
2	FTEEYQLFEELGKGAFSVVR(13)-ITAAEALKHPWISHR(8)	kinase	kinase	21-267	0,48	0,45
3	DLKPENLLLASKLK(12)-DLINKMLTINPSK(5)	kinase	kinase	146-250	0,41	0,34
4	DLKPENLLLASK(3)-LLKHPNIVR(3)	kinase	kinase	137-68	0,37	0,30
5	DLKPENLLLASKLK(12)-DLKPENLLLASK(3)	kinase	kinase	146-137	0,37	0,29
6	FTEEYQLFEELGKGAFSVVR(13)-DLINKMLTINPSK(5)	kinase	kinase	21-250	0,36	0,24
7	FTEEYQLFEELGKGAFSVVR(13)-VLAGQEYAAKIINTK(10)	kinase	kinase	21-42	0,33	0,24
8	MLTINPSKR(8)-GSGSGMTR(1)	kinase	kinase	258-1	0,33	0,20
9	NFSGGKSGGNK(6)-DHQKLER(4)	linker	kinase	317-56	0,31	0,27
10	DLINKMLTINPSK(5)-IINTKK(5)	kinase	kinase	250-47	0,30	0,19
11	ITAAEALKHPWISHR(8)-VLAGQEYAAKIINTK(10)	kinase	kinase	267-42	0,30	0,25
12	FTEEYQLFEELGKGAFSVVR(13)-MLTINPSKR(8)	kinase	kinase	21-258	0,30	0,24
13	VLAGQEYAAKIINTK(10)-IINTKK(5)	kinase	kinase	42-47	0,29	0,23
14	FTEEYQLFEELGKGAFSVVR(13)-LKGAAVK(2)	kinase	kinase	21-148	0,29	0,21
15	DHQKLER(4)-KQEIIK(1)	kinase	hub	56-347	0,28	0,15
16	LLKHPNIVR(3)-KQEIIK(1)	kinase	hub	68-347	0,28	0,12
17	FTEEYQLFEELGKGAFSVVR(13)-DLKPENLLLASK(3)	kinase	kinase	21-137	0,27	0,23
18	AGAYDFPSPEWDTVTPEAKDLINK(19)-DHQKLER(4)	kinase	kinase	245-56	0,26	0,17
19	ESSESTNTTIEDEDTKVR(16)-DLKPENLLLASK(3)	hub	kinase	344-137	0,26	0,18
20	MLTINPSKR(8)-IINTKK(5)	kinase	kinase	258-47	0,25	0,18
21	FTEEYQLFEELGKGAFSVVR(13)-DLKPENLLLASKLK(12)	kinase	kinase	21-146	0,25	0,15
22	AGAYDFPSPEWDTVTPEAKDLINK(19)-GSGSGMTR(1)	kinase	kinase	245-1	0,25	0,26
23	LKGAILTTMLATR(2)-DHQKLER(4)	regulatory	kinase	300-56	0,25	0,16
24	VLAGQEYAAKIINTK(10)-MLTINPSKR(8)	kinase	kinase	42-258	0,24	0,20
25	LYQQIKAGAYDFPSPEWDTVTPEAK(6)-VLAGQEYAAKIINTK(10)	kinase	kinase	226-42	0,24	0,25
26	GSGSGMTR(1)-LKGAAVK(2)	kinase	kinase	1-148	0,24	0,25
27	AGAYDFPSPEWDTVTPEAKDLINK(19)-DLKPENLLLASK(3)	kinase	kinase	245-137	0,23	0,19
28	FTEEYQLFEELGKGAFSVVR(13)-KSDGVK(1)	kinase	linker	21-323	0,23	0,20
29	FTEEYQLFEELGKGAFSVVR(13)-LLKHPNIVR(3)	kinase	kinase	21-68	0,23	0,21
30	DLINKMLTINPSK(5)-LKGAAVK(2)	kinase	kinase	250-148	0,23	0,15
31	VLAGQEYAAKIINTK(10)-LKGAAVK(2)	kinase	kinase	42-148	0,23	0,18
32	VLAGQEYAAKIINTK(10)-NFSGGKSGGNK(6)	kinase	linker	42-317	0,23	0,14
33	VLAGQEYAAKIINTK(10)-DLKPENLLLASK(3)	kinase	kinase	42-137	0,23	0,22
34	ITAAEALKHPWISHR(8)-IINTKK(5)	kinase	kinase	267-47	0,22	0,23
35	LYQQIKAGAYDFPSPEWDTVTPEAK(6)-DLKPENLLLASK(3)	kinase	kinase	226-137	0,22	0,17
36	DLINKMLTINPSK(5)-DLKPENLLLASK(3)	kinase	kinase	250-137	0,21	0,25
37	LKGAILTTMLATR(2)-DLKPENLLLASK(3)	regulatory	kinase	300-137	0,21	0,19
38	AGAYDFPSPEWDTVTPEAKDLINK(19)-DLKPENLLLASKLK(12)	kinase	kinase	245-146	0,20	0,16
39	LYQQIKAGAYDFPSPEWDTVTPEAK(6)-DLKPENLLLASKLK(12)	kinase	kinase	226-146	0,19	0,15
40	LYQQIKAGAYDFPSPEWDTVTPEAK(6)-LKGAAVK(2)	kinase	kinase	226-148	0,19	0,12
41	LLKHPNIVR(3)-LKGAAVK(2)	kinase	kinase	68-148	0,18	0,20

42	VLAGQEYAAKIINTK(10)-LLKHPNIVR(3)	kinase	kinase	42-68	0,17	0,15
43	DLKPENLLLASKLK(12)-MLTINPSKR(8)	kinase	kinase	146-258	0,16	0,16
44	VLAGQEYAAKIINTK(10)-KQEIIK(1)	kinase	hub	42-347	0,16	0,15
45	DHQKLER(4)-KSDGVK(1)	kinase	linker	56-323	0,16	0,12
46	VLAGQEYAAKIINTK(10)-KSDGVK(1)	kinase	linker	42-323	0,14	0,15

Supplementary File 1I

pT286 Heterotypic peptides (30 min)					
**#**	**Peptides**	**domain_a**	**domain_b**	**link**	**R**
1	QETVDCLKK(8)-LLKHPNIVR(3)	regulatory	kinase	291-68	0,15
2	LYQQIKAGAYDFPSPEWDTVTPEAK(6)-QETVDCLKK(8)	kinase	regulatory	226-291	0,13
3	VLAGQEYAAKIINTK(10)-QETVDCLKK(8)	kinase	regulatory	42-291	0,10
4	DLKPENLLLASKLK(12)-QETVDCLKK(8)	kinase	regulatory	146-291	0,10
5	QETVDCLKK(8)-MLTINPSKR(8)	regulatory	kinase	291-258	0,07
6	DLINKMLTINPSK(5)-QETVDCLKK(8)	kinase	regulatory	250-291	0,05

Supplementary File 1J

pT286 Heterotypic peptides (150 min)					
**#**	**Peptides**	**domain_a**	**domain_b**	**crosslinked lysines**	**R**
1	VLAGQEYAAKIINTK(10)-QETVDCLKK(8)	kinase	regulatory	42-291	0,39
2	QETVDCLKK(8)-LLKHPNIVR(3)	regulatory	kinase	291-68	0,35
3	QETVDCLKK(8)-DHQKLER(4)	regulatory	kinase	291-56	0,26
4	ITAAEALKHPWISHR(8)-QETVDCLKK(8)	kinase	regulatory	267-291	0,18
5	DLINKMLTINPSK(5)-QETVDCLKK(8)	kinase	regulatory	250-291	0,15
6	QETVDCLKK(8)-LKGAAVK(2)	regulatory	kinase	291-148	0,11
